# The global, regional, and national impact of laryngeal cancer from 1990 to 2021, along with forecasts for 2050: a comprehensive analysis for the Global Burden of Disease 2021 research

**DOI:** 10.3389/fonc.2025.1627009

**Published:** 2025-08-22

**Authors:** Tingting Duan, Yuxia Zou, Xuan Liu, Yue Du, Ruihu Li, Jingxiang Cai, Jumei Li, Xiaofeng Wang, Xuejun Zhou, Shun Ding

**Affiliations:** Department of Otolaryngology, Head and Neck Surgery, The First Affiliated Hospital, Hainan Medical University, Haikou, China

**Keywords:** laryngeal cancer, disease burden, incidence, prevalence, deaths, disability-adjusted life years

## Abstract

**Background:**

Laryngeal cancer (LC), the second most common head and neck malignancy, with significant global geographical disparities in incidence and mortality, was analyzed using 2021 Global Burden of Disease (GBD) data to assess Socio-Demographic Index (SDI) correlations and project disease burden through 2050, aiming to reduce its global impact.

**Methods:**

Using data from the GBD 2021, we investigated the incidence, prevalence, deaths, and Disability-Adjusted Life Years (DALYs) of LC, as well as trends, age-sex-time patterns, driving factors, and projections up to 2050.

**Results:**

In 2021, LC increased with age and decreased after the age of 70. The incidence and mortality rates of male patients are much higher than those of female patients, and the gap between men and women gradually narrows after the age of 70. From 1990 to 2021, the overall disease burden of LC globally showed a downward trend and is expected to further decline by 2050. However, there are significant differences among different countries and regions. In high SDI regions, the disease burden gradually decreases after reaching its peak, while in low SDI regions, the reduction or increase in the disease burden is relatively small. Population growth and aging are the main factors leading to the increase in the LC burden, but epidemiological changes have alleviated this burden to a certain extent. Smoking and excessive alcohol consumption are the main risk factors for LC.

**Conclusions:**

While the global LC burden has moderated, significant disparities persist across SDI regions. Healthcare quality improvements and targeted prevention, with this analysis offering evidence-based guidance for public health strategy formulation.

## 1 Introduction

Laryngeal cancer (LC) ranks as the second most prevalent malignant neoplasm in the head and neck region, characterized by an alarming rise in incidence and death rate (1). This aggressive malignancy is particularly susceptible to local infiltration, metastasis to cervical lymph nodes, and resistance to chemotherapeutic agents, all of which significantly contribute to the dismal prognoses faced by affected individuals (2). While early intervention yields a favorable effect on LC, the outlook for advanced-stage treatment remains grim (3). Moreover, LC-related complications can inflict considerable pain and distress upon patients, manifesting as persistent coughing, tracheal disorders, difficulties in swallowing, and challenges in breathing (4).

The global burden of LC exhibits significant geographic heterogeneity. For instance, the death rate among males in Hungary is as high as 6 per 100,000, whereas in Nordic countries such as Sweden, it is less than 1 per 100,000 (5). In South America, Brazil and Uruguay emerge as high-incidence areas for LC within the Southern Hemisphere (6, 7). Surveillance data indicate that the incidence rates of LC have been steadily declining in developed countries (for example, in American males, the age-standardized incidence rate (ASIR) decreased from 5.98 per 100,000 in 2000 to 3.3 per 100,000 in 2019). However, an upward trend is observed among females and in some developing countries (such as Sri Lanka), which may be associated with variations in tobacco control policies and changes in HPV infection rates (8, 9). Long-term monitoring of these disparities can provide crucial evidence for formulating targeted prevention and control strategies for LC (10).

Despite the gradual improvement in global healthcare accessibility (as measured by the HAQ index), the disparity in HAQ scores among countries with different Socio-demographic Index (SDI) values remained over 60% in 2019. Developed countries generally have more comprehensive epidemiological studies on LC, whereas data from developing countries, especially in Africa and the Middle East, are notably scarce. For instance, an analysis of LC trends in Iraq showed that only cervical cancer and laryngeal cancer exhibited a declining trend; however, the reasons behind this decline remain unclear. Moreover, there is a limited amount of research predicting future disease burdens, particularly under the dual pressures of population aging and risk factor changes. It is imperative to address these gaps through multidimensional approaches, such as cohort modeling and big data analysis.

Despite the gradual improvement in the Healthcare Access and Quality (HAQ) Index, disparities in HAQ scores among countries with different Socio-demographic Index (SDI) values remained over 60% in 2019 (11). Developed countries have relatively comprehensive epidemiological studies on LC, whereas data from developing countries, especially in Africa and the Middle East, are notably lacking (11, 12). For example, trend analysis of LC in Iraq showed that only cervical cancer and laryngeal cancer exhibited a declining trend; however, the reasons for this decline remain unclear (13). Furthermore, there is limited research predicting future disease burdens, particularly in the context of the dual pressures of population aging and risk factor changes. It is imperative to address these gaps through multidimensional approaches, such as cohort modeling and big data analysis (14).

As one of the common malignant tumors in the head and neck region, the epidemiological characteristics and disease burden of LC have received extensive attention in recent years. Existing studies, such as those by Han et al., have conducted systematic analyses of laryngeal cancer mortality at the global, regional, and national levels from 1990 to 2021 based on the Global Burden of Diseases (GBD) study, revealing the spatiotemporal trends of the disease and its potential associations with sociodemographic factors (15). In addition, Jang et al. focused on the global burden of pharyngeal cancer and used GLOBOCAN 2022 data to predict the incidence trends from the present to 2050, providing a reference basis for the prevention and control strategies of related cancers (16). Sun et al. also used GBD data and the APC model to analyze laryngeal cancer, but their research subjects were mainly the elderly population (17). In contrast, in the study, by leveraging the most recent 2021 GBD data, our research not only undertakes descriptive analyses but also incorporates sophisticated approaches, including age-period-cohort analysis, Joinpoint regression analysis, and decomposition analysis, to meticulously explore trends in the disease burden alongside their underlying determinants. We conduct a thorough investigation into the trends across age, gender, and time concerning disease burden and their correlation with the SDI. Furthermore, for the first time, we project the anticipated future burden of LC. The findings from these analyses are intended to furnish solid scientific evidence that supports the judicious allocation of public health resources and the formulation of effective preventive measures.

## 2 Methods

### 2.1 Overview

The data for this study comes from the GBD 2021(https://vizhub.healthdata.org/gbd-results/) study, which provides a comprehensive assessment of population, incidence, and death rates for 371 injuries and diseases and 88 risk factors, forming a disease burden database covering 204 countries and regions (18). This database integrates monitoring and research results from multiple institutions, utilizing a variety of data sources including systematic reviews of literature, survey data, surveillance data, and clinical data from inpatient and outpatient visits, among others. During its construction, significant attention was paid to the quality and completeness of the data. Advanced statistical models were employed to adjust and impute data, thereby enhancing the accuracy and representativeness of the dataset.

### 2.2 Data acquisition and preliminary analysis

Estimates regarding the incidence, prevalence, deaths, and disability adjusted life years (DALYs) of LC, along with their 95% uncertainty intervals (UIs), were sourced from the GBD 2021 database.

Descriptive analyses were conducted on the global LC prevalence, incidence, deaths, and DALYs stratified by gender and age. Additionally, these disease burden indicators were analyzed across income levels and geographic regions. To assess temporal trends from 1990 to 2021, we calculated the estimated annual percentage change (EAPC) for age-standardized incidence, prevalence, deaths, and DALYs. The EAPC, derived from a regression model fitted with natural logarithms [ In(rate)=α+β(calendar year)+ϵ ], represents the annual trend magnitude, calculated as 100*(exp(β)−1) (19).

An upward trend was defined when both the EAPC estimate and the lower bound of its 95% confidence interval (CI) exceeded 0, whereas a downward trend was identified when both the EAPC estimate and the upper 95% CI bound were below 0. This metric quantifies the pace and fundamental characteristics of cancer progression, enabling predictions of future trajectories and evidence-based guidance for public health interventions.

### 2.3 Joinpoint regression analysis

Temporal trends in incidence, prevalence, deaths, and DALYs were analyzed using the Joinpoint Regression software (version 4.7.0.0), which identifies statistically significant inflection points (“joinpoints”) by fitting segmented linear models on a logarithmic scale. Joinpoints represent optimal inflection points where significant trend changes occurred, determined through segmented regression analysis (20). The natural logarithm of the observed rate was fitted to the regression line using the calendar year as the regression variable. The Joinpoint regression model formula is: $E[y/x]=\beta_{0}+\beta_{1}x+\delta_{1}{\left(x-\tau_{1}\right)}^{+}+\ldots +\delta_{k}{\left(x-\tau_{k}\right)}^{+}$ , where y represents age-standardized incidence, prevalence, deaths, or DALY rates; x denotes calendar year; β is the intercept; δ_i_ (i=1, 2,…, k) indicates regression coefficients for segmented intervals; τ_i_ (i=1, 2,…, k) marks joinpoint years; and k signifies the number of inflection points.

The model calculates annual percent change (APC), average annual percent change (AAPC), and corresponding 95% CI to characterize trends across identified segments. An upward trend was defined when either APC or AAPC > 0 with the 95% CI excluding 0, while a downward trend was identified when APC or AAPC < 0 with the 95% CI excluding 0. Age-standardized rates were considered temporally stable if the 95% CI included 0.

### 2.4 Age-period-cohort model

The age-period-cohort (APC) model based on the Poisson distribution can simultaneously estimate the net effect of age, period, and cohort on the burden of disease (21). The expression of the APC model is usually written as follows: Y=log(M)=μ+α×age1+β×period1+γ×cohort1+∈ , here M denotes the rate of the corresponding age group, μ denotes the intercept term, and α, β and γ denote the age, period and cohort effects, respectively, and ϵ is the random error. Rate, μ denotes the intercept term, α, β, and γ denote the age, period, and cohort effects, respectively, and ϵ is the random error.

The APC model requires equal time intervals for age, period and cohort. In the GBD 2021 database, the incidence and deaths rates of LC were not recorded for population aged 0–19 years, so the age analysis started from 20 years. At the same time, as the age group 95 years and above did not meet the data format requirements of the APC model, this age group was excluded from the analysis. As a result, the age groups were categorized into 15 groups (starting at age 20, with age groups every 5 years). To match the age classification, the data were further collapsed into 6 periods (1992-1996, 1997-2001, 2002-2006, 2007-2011, 2012-2016, and 2017-2021) and 20 equally spaced birth cohorts (starting in 1902, with one birth cohort every 5 years).

### 2.5 Bayesian age-period-cohort model

Based on trends in the disease burden of LC from 1990 to 2021, we utilized the BAPC model to forecast the incidence, prevalence, deaths, and DALYs of LC from 2022 to 2050 (22). The BAPC model was executed using the INLA package in R software. The model was constructed based on the number of cases per age group, period, and gender, assuming that these follow a Poisson process, with means equal to the product of the population at risk and the estimated incidence rate. The logarithm of the incidence rates was estimated as a linear combination of gender-specific intercepts, overdispersion effects, age effects, period effects, and cohort effects. For age, period, and cohort effects, a second-order random walk prior was adopted, with their sum constrained to zero; log-gamma priors were applied to the precision parameters, where the scale and shape parameters for age, period, and cohort effects were set to 1 and 0.00005 respectively, whereas those for the overdispersion effect were set to 1 and 0.005. An average-zero Gaussian prior distribution was assumed for the overdispersion parameter.

### 2.6 Decomposition analysis

The study analyzed the data using a robust decomposition method that attributed the differences in incidence, prevalence, deaths, and DALYs between the two time points to changes in three independent factors: Aging (A), Population (P), and Epidemiological change (M).

The basic equation is as follows: A=Ma+Iam+Ipa+Ipam , where A denotes the main effect of population ageing, Ma is the effect of age-specific death rates, Iam is the interaction effect of ageing and age-specific rates, Ipa is the interaction effect of ageing and population growth, and Ipam is the joint interaction effect of the three. p=Mp+Ipm+Ipa+Ipam , where P denotes the main effect of population growth, Mp is the effect of population growth, Ipm is the interaction effect of population with age-specific rates, Ipa is the interaction effect of population with ageing, and Ipam is the joint interaction effect of all three. M=Mm+Ipm+Iam+Ipam , where M denotes the main effect of age-specific rate change, Mm is the effect of age-specific rates, Ipm is the age-specific rate interaction effect with population, Iam is the interaction effect of age-specific rates with ageing, and Ipam is the joint interaction effect of all three.

This method decomposes disease-related data changes into factors caused by A, P, and M. The results of the decomposition include the effect of each factor on the disease. The decomposition results include the absolute and relative contribution of each factor to disease (23).

### 2.7 Statistical analysis

All statistical evaluations and data representations were conducted utilizing R (version 4.4.2) and JD_GBDR (V2.45.1, Jingding Medical Technology Co., Ltd.). Descriptive analytics were compiled for all principal variables, with findings expressed as means accompanied by 95% UIs or 95% CIs. For the analysis of trends, a p-value threshold of less than 0.05 was deemed statistically significant.

## 3 Results

### 3.1 The global scope of LC

In 2021, there were a total of 200,883 newly identified cases of LC worldwide (95% UI: 186,941–216,097), with 171,788 cases in males (95% UI: 159,470–186,042) and 29,094 cases in females (95% UI: 24,974–33,679). The cumulative number of existing LC cases globally reached 1,103,683 cases (95% UI: 1,033,145–1,186,559), which includes 939,924 cases in males (95% UI: 876,345–1,011,203) and 163,759 cases in females (95% UI: 144,050–186,894). Deaths attributed to LC numbered 117,251 cases (95% UI: 109,354–125,952), including 100,392 male fatalities (95% UI: 93,350–108,829) and 16,858 female fatalities (95% UI: 14,208–19,875). Moreover, LC was responsible for a considerable total of 3,143,308 DALYs (95% UI: 2,922,791–3,383,513), with males accounting for 2,694,178 years (95% UI: 2,491,889–2,926,037) and females for 449,130 years (95% UI: 377,884–540,000). In the year 2021, the worldwide age-standardized incidence of LC was recorded at 2.29 per 100,000 individuals (95% UI: 2.13-2.47), while the age-standardized prevalence stood at 12.56 per 100,000 individuals (95% UI: 11.76-13.49). Additionally, the age-standardized death rate was noted at 1.35 per 100,000 individuals (95% UI: 1.26-1.45), and the age-standardized DALYs were calculated at 35.80 per 100,000 person-years (95% UI: 33.29-38.54). From 1990 to 2021, the EAPC and its confidence intervals for all metrics related to LC were negative, signifying a notable global reduction in the burden of LC (**Table 1**).

**Table 1:** Age-standardized results for the burden of LC across the global populace, five SDI regions, and 21 GBD regions.

Location	Age-standardized incidence			Age-standardized prevalence			Age-standardized deaths			Age-standardized DALYs		
	1990 (per 100,000 population, 95% UI)	2021 (per 100,000 population, 95% UI)	EAPCs (95% CI)	1990 (per 100,000 population, 95% UI)	2021 (per 100,000 population, 95% UI)	EAPCs (95% CI)	1990 (per 100,000 population, 95% UI)	2021 (per 100,000 population, 95% UI)	EAPCs (95% CI)	1990 (per 100,000 population, 95% UI)	2021 (per 100,000 population, 95% UI)	EAPCs (95% CI)
Global	3.07 (2.92,3.23)	2.29 (2.13,2.47)	-1.09 (-1.17,-1.01)	15.27 (14.54,16.06)	12.56 (11.76,13.49)	-0.76 (-0.83,-0.70)	2.15 (2.01,2.28)	1.35 (1.26,1.45)	-1.66 (-1.74,-1.58)	59.30 (55.47,63.10)	35.80 (33.29,38.54)	-1.82 (-1.90,-1.73)
High SDI	3.64 (3.53,3.75)	2.36 (2.23,2.46)	-1.52 (-1.60,-1.45)	21.48 (20.76,22.22)	15.56 (14.82,16.16)	-1.15 (-1.25,-1.06)	1.61 (1.55,1.66)	0.75 (0.71,0.79)	-2.59 (-2.68,-2.50)	44.84 (43.45,46.30)	19.52 (18.47,20.42)	-2.81 (-2.89,-2.74)
High-middle SDI	3.84 (3.66,4.02)	2.53 (2.28,2.79)	-1.57 (-1.67,-1.47)	19.05 (18.22,19.93)	14.60 (13.23,16.03)	-1.03 (-1.13,-0.94)	2.70 (2.57,2.84)	1.29 (1.18,1.42)	-2.67 (-2.77,-2.57)	76.97 (73.37,80.79)	33.81 (30.72,37.12)	-2.99 (-3.11,-2.88)
Middle SDI	2.22 (2.04,2.39)	2.03 (1.81,2.27)	-0.40 (-0.49,-0.31)	9.55 (8.77,10.29)	10.32 (9.23,11.56)	0.16 (0.07,0.26)	1.96 (1.80,2.10)	1.34 (1.20,1.49)	-1.35 (-1.41,-1.30)	51.12 (46.83,55.19)	34.12 (30.71,37.96)	-1.45 (-1.51,-1.39)
Low-middle SDI	2.72 (2.33,3.17)	2.49 (2.23,2.79)	-0.32 (-0.41,-0.24)	11.08 (9.65,12.80)	11.12 (10.03,12.38)	-0.04 (-0.14,0.06)	2.56 (2.19,2.99)	2.11 (1.90,2.37)	-0.66 (-0.72,-0.60)	69.50 (59.39,81.33)	56.07 (50.13,63.17)	-0.73 (-0.78,-0.67)
Low SDI	2.33 (1.83,2.86)	1.99 (1.72,2.29)	-0.64 (-0.74,-0.54)	9.12 (7.35,11.09)	8.36 (7.24,9.61)	-0.42 (-0.52,-0.31)	2.25 (1.78,2.76)	1.82 (1.56,2.10)	-0.79 (-0.87,-0.70)	60.89 (47.73,75.04)	47.26 (40.50,54.80)	-0.96 (-1.05,-0.88)
Andean Latin America	1.26 (1.10,1.45)	0.81 (0.63,1.02)	-1.66 (-1.95,-1.36)	4.96 (4.33,5.66)	3.73 (2.93,4.74)	-1.16 (-1.46,-0.87)	1.21 (1.05,1.39)	0.63 (0.49,0.79)	-2.27 (-2.53,-2.02)	28.77 (25.02,32.95)	14.65 (11.31,18.37)	-2.42 (-2.69,-2.15)
Australasia	2.08 (1.91,2.28)	1.15 (1.02,1.28)	-1.91 (-2.00,-1.82)	11.48 (10.57,12.55)	7.40 (6.58,8.18)	-1.39 (-1.49,-1.29)	1.18 (1.09,1.30)	0.49 (0.43,0.54)	-2.95 (-3.05,-2.85)	30.91 (28.09,34.02)	11.53 (10.23,12.91)	-3.23 (-3.32,-3.13)
Caribbean	3.72 (3.42,4.05)	4.07 (3.51,4.75)	0.43 (0.32,0.54)	16.81 (15.56,18.29)	20.49 (17.72,23.95)	0.80 (0.67,0.93)	2.93 (2.70,3.18)	2.69 (2.33,3.13)	-0.15 (-0.25,-0.05)	72.57 (66.93,78.93)	67.98 (58.33,79.96)	-0.04 (-0.15,0.06)
Central Asia	3.38 (3.23,3.54)	1.52 (1.36,1.70)	-2.62 (-2.81,-2.43)	15.26 (14.59,16.00)	7.57 (6.84,8.37)	-2.32 (-2.47,-2.17)	2.81 (2.69,2.94)	1.18 (1.05,1.31)	-2.86 (-3.10,-2.62)	83.67 (80.18,87.67)	32.31 (28.65,36.25)	-3.20 (-3.43,-2.96)
Central Europe	4.92 (4.67,5.20)	4.46 (4.07,4.87)	-0.44 (-0.57,-0.30)	24.04 (22.74,25.50)	25.25 (23.18,27.54)	0.07 (-0.08,0.22)	3.70 (3.51,3.90)	2.50 (2.29,2.71)	-1.43 (-1.53,-1.32)	111.11 (105.61,117.71)	69.93 (63.58,76.15)	-1.69 (-1.81,-1.56)
Central Latin America	2.31 (2.22,2.39)	1.22 (1.07,1.40)	-2.51 (-2.66,-2.36)	9.37 (9.05,9.66)	5.69 (5.02,6.49)	-2.08 (-2.25,-1.92)	2.11 (2.03,2.18)	0.94 (0.83,1.07)	-3.02 (-3.15,-2.89)	49.13 (47.41,50.69)	21.88 (19.22,25.07)	-3.06 (-3.19,-2.92)
Central Sub-Saharan Africa	1.70 (1.25,2.22)	1.47 (1.10,1.88)	-0.47 (-0.64,-0.29)	6.32 (4.72,8.21)	5.92 (4.52,7.52)	-0.20 (-0.39,-0.01)	1.68 (1.26,2.18)	1.38 (1.04,1.76)	-0.63 (-0.77,-0.48)	43.75 (31.29,57.68)	36.27 (26.70,46.84)	-0.60 (-0.74,-0.46)
East Asia	1.81 (1.51,2.12)	1.78 (1.41,2.24)	0.02 (-0.12,0.17)	7.85 (6.53,9.12)	9.83 (7.85,12.23)	0.86 (0.71,1.02)	1.57 (1.31,1.83)	0.93 (0.74,1.16)	-1.74 (-1.82,-1.65)	39.94 (33.02,46.95)	22.59 (17.73,28.27)	-1.90 (-1.99,-1.80)
Eastern Europe	4.64 (4.46,4.81)	2.96 (2.61,3.33)	-2.06 (-2.30,-1.82)	22.48 (21.59,23.44)	16.07 (14.30,17.85)	-1.60 (-1.85,-1.35)	3.36 (3.25,3.48)	1.73 (1.54,1.95)	-2.81 (-3.05,-2.58)	106.81 (103.18,110.57)	51.29 (45.47,57.66)	-3.09 (-3.33,-2.84)
Eastern Sub-Saharan Africa	1.79 (1.41,2.17)	1.38 (1.10,1.76)	-1.05 (-1.14,-0.97)	6.83 (5.45,8.11)	5.79 (4.65,7.35)	-0.71 (-0.79,-0.64)	1.73 (1.36,2.10)	1.27 (1.01,1.60)	-1.20 (-1.28,-1.13)	47.38 (36.92,57.79)	34.49 (27.03,44.42)	-1.24 (-1.31,-1.16)
High-income Asia Pacific	2.21 (2.01,2.41)	1.35 (1.17,1.51)	-1.87 (-2.08,-1.65)	13.52 (12.32,14.66)	9.33 (8.21,10.38)	-1.41 (-1.64,-1.18)	0.84 (0.74,0.92)	0.31 (0.27,0.35)	-3.61 (-3.78,-3.44)	20.57 (17.86,23.02)	7.10 (6.22,7.97)	-3.83 (-3.99,-3.67)
High-income North America	4.06 (3.94,4.16)	2.76 (2.62,2.86)	-1.55 (-1.68,-1.43)	25.28 (24.42,26.03)	18.32 (17.51,19.00)	-1.34 (-1.48,-1.20)	1.36 (1.31,1.40)	0.76 (0.72,0.80)	-2.14 (-2.21,-2.07)	37.33 (36.09,38.39)	19.77 (18.88,20.65)	-2.31 (-2.39,-2.24)
North Africa and Middle East	2.81 (2.34,3.30)	2.61 (2.30,2.98)	-0.27 (-0.34,-0.19)	12.35 (10.46,14.23)	13.76 (12.22,15.55)	0.33 (0.27,0.39)	2.41 (1.99,2.83)	1.61 (1.42,1.84)	-1.34 (-1.40,-1.28)	61.60 (51.28,71.34)	39.45 (34.38,45.16)	-1.51 (-1.57,-1.45)
Oceania	0.64 (0.48,0.83)	0.56 (0.44,0.74)	-0.45 (-0.50,-0.40)	2.57 (2.00,3.27)	2.35 (1.84,3.01)	-0.36 (-0.42,-0.31)	0.59 (0.45,0.77)	0.51 (0.40,0.68)	-0.48 (-0.52,-0.44)	14.13 (10.55,18.58)	12.02 (9.21,15.94)	-0.56 (-0.60,-0.52)
South Asia	3.44 (2.90,4.02)	2.99 (2.61,3.42)	-0.61 (-0.75,-0.47)	14.03 (12.06,16.26)	13.54 (11.90,15.43)	-0.28 (-0.43,-0.13)	3.25 (2.73,3.80)	2.51 (2.20,2.87)	-0.98 (-1.08,-0.87)	88.93 (75.45,103.97)	67.29 (58.66,77.40)	-1.05 (-1.15,-0.95)
Southeast Asia	1.52 (1.31,1.71)	1.58 (1.36,1.86)	0.08 (0.03,0.13)	6.58 (5.78,7.39)	7.83 (6.79,9.12)	0.54 (0.48,0.59)	1.31 (1.13,1.48)	1.09 (0.95,1.28)	-0.66 (-0.69,-0.64)	33.90 (29.46,38.31)	27.61 (23.90,32.69)	-0.72 (-0.74,-0.70)
Southern Latin America	4.09 (3.79,4.44)	2.15 (1.95,2.36)	-2.14 (-2.30,-1.98)	20.14 (18.59,21.84)	12.16 (11.14,13.28)	-1.76 (-1.94,-1.58)	3.07 (2.86,3.33)	1.34 (1.23,1.46)	-2.64 (-2.78,-2.50)	85.38 (79.24,92.86)	34.00 (30.86,37.32)	-3.02 (-3.16,-2.87)
Southern Sub-Saharan Africa	2.23 (1.87,2.92)	2.07 (1.83,2.35)	-0.46 (-0.73,-0.20)	9.99 (8.35,12.78)	9.26 (8.20,10.45)	-0.44 (-0.59,-0.29)	1.95 (1.64,2.53)	1.71 (1.51,1.93)	-0.63 (-0.95,-0.32)	55.59 (46.65,71.65)	48.13 (41.99,55.22)	-0.69 (-1.01,-0.37)
Tropical Latin America	3.29 (3.15,3.43)	2.99 (2.80,3.17)	-0.32 (-0.41,-0.23)	14.24 (13.65,14.80)	14.78 (13.86,15.65)	0.07 (-0.03,0.17)	2.80 (2.68,2.92)	2.15 (2.01,2.27)	-0.82 (-0.91,-0.72)	77.36 (74.31,80.40)	58.65 (55.12,62.14)	-0.92 (-1.02,-0.81)
Western Europe	4.98 (4.78,5.18)	2.99 (2.80,3.18)	-1.63 (-1.71,-1.55)	28.94 (27.70,30.16)	20.17 (19.04,21.36)	-1.17 (-1.26,-1.07)	2.38 (2.28,2.46)	0.99 (0.92,1.05)	-2.84 (-2.96,-2.72)	67.26 (64.52,70.00)	25.72 (24.02,27.36)	-3.14 (-3.24,-3.03)
Western Sub-Saharan Africa	1.21 (0.97,1.49)	1.19 (0.97,1.43)	0.07 (-0.02,0.15)	4.81 (3.91,5.87)	4.90 (3.96,5.89)	0.17 (0.10,0.23)	1.17 (0.94,1.44)	1.11 (0.92,1.33)	-0.02 (-0.12,0.09)	31.11 (24.58,38.66)	27.96 (22.29,33.92)	-0.25 (-0.34,-0.15)

### 3.2 Regional burden of LC

In high SDI regions, the age - age-standardized incidence rate of LC dropped from 3.64 per 100,000 population in 1990 (95% Ul: 3.53 - 3.75) to 2.36 per 100,000 population in 2021 (95% Ul: 2.23 - 2.46), with an EAPCs of - 1.52%. Similarly, the age - age-standardized prevalence, deaths, and DALYs in this region also exhibited a significant downward trend, decreasing by - 1.15%, - 2.59%, and - 2.81% respectively. As the SDI level declined, relevant indicators in the high-middle SDI region and the middle SDI region also showed downward trends to varying degrees. However, compared with the high SDI region, the decline was slightly smaller. It is especially worth noting that in the middle SDI region, although the incidence rate decreased, the prevalence rate increased slightly. This may indicate the existence of treatment effects or extended survival periods. For the low-middle SDI and low SDI regions, although the age - age-standardized incidence rate, deaths, and DALYs of LC in these regions also decreased, the decline was relatively slow. Moreover, the prevalence in these two regions remained almost unchanged or decreased only slightly. This may reflect the challenges of LC treatment in lower SDI regions and areas that need more attention (**Table 1** and **Supplementary Figure S1**).

In 2021, health indicators in different regions around the world presented significant differences. The Central European region ranked first in both the age - standardized incidence rate (4.46 per 100,000 population, 95% UI: 4.07 - 4.87) and the prevalence rate (25.25 per 100,000 population, 95% UI: 23.18 - 27.54). Moreover, it had the highest DALYs (69.93 per 100,000 population, 95% UI: 63.58 - 76.15). Oceania had the lowest incidence rate (0.56 per 100,000 population, 95% UI: 0.44 - 0.74) and prevalence rate (2.35 per 100,000 population, 95% UI:1.84 - 3.01). The Caribbean region recorded the highest age - standardized deaths rate (2.69 per 100,000 population, 95% UI:2.33 - 3.03). In contrast, the high - income Asia Pacific region was at the lowest level not only in terms of the deaths rate (0.31 per 100,000 population, 95% UI:0.27 - 0.35) but also in terms of the DALYs burden (7.10 per 100,000 population, 95% UI: 6.22 - 7.97). According to the time trend, the incidence in 4 regions and the prevalence in 7 regions either have no significant change or show an upward trend. However, all the other regions show a downward trend. Among these regions, Central Asia has the largest decline in the incidence and prevalence, and High - income Asia Pacific has the largest decline in deaths and DALYs (**Table 1** and **Supplementary Figure S1**).

### 3.3 Country burden of LC

**Figure 1:**
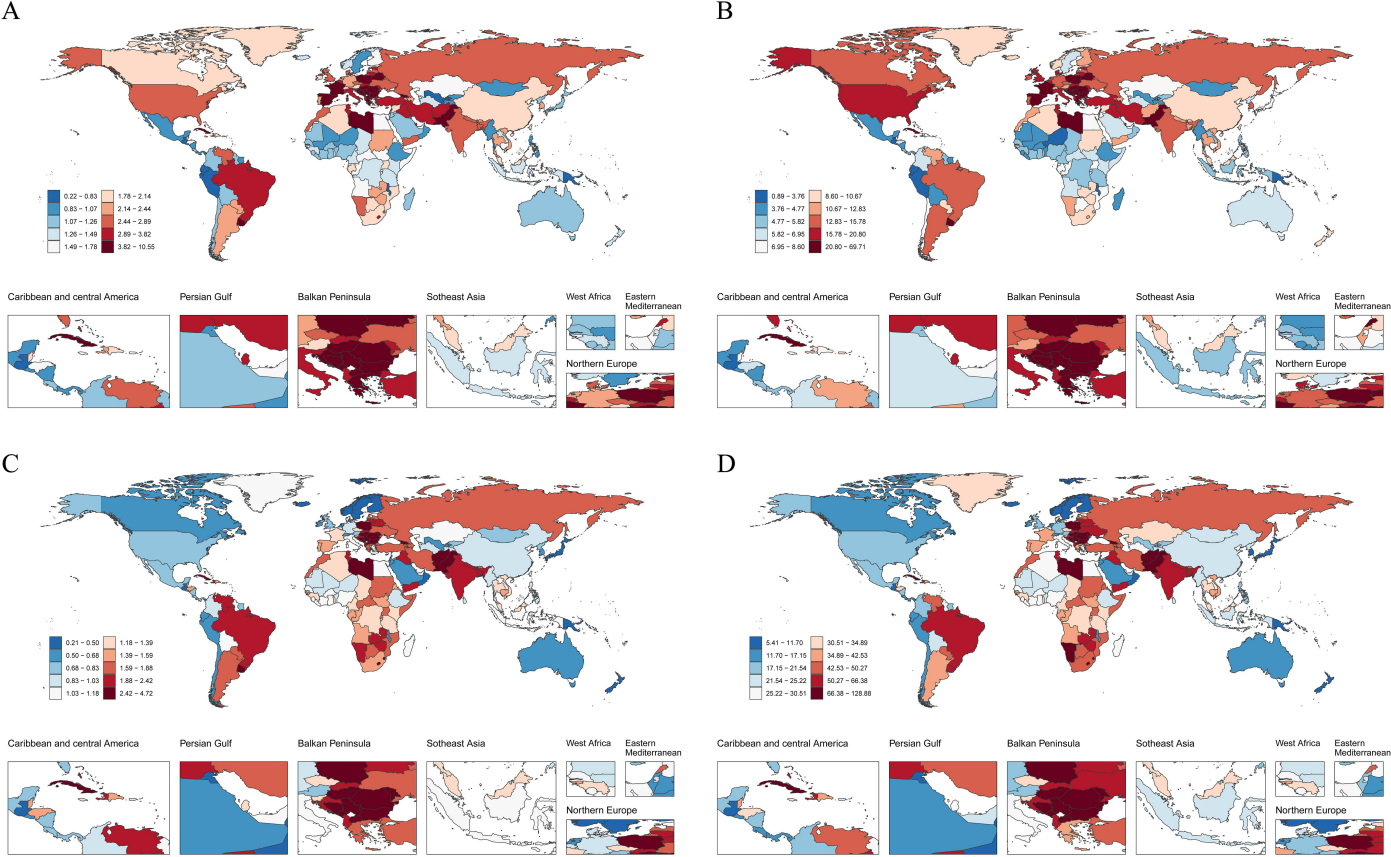
The global impact of LC across 204 countries and territories in 2021. **(A)** Age - standardized incidence. **(B)** Age - standardized prevalence. **(C)** Age - standardized deaths. **(D)** Age - standardized DALYs.

In 2021, there were differences in the age - standardized indicators of LC among 204 countries. Based on the cross - sectional results at that time, the Principality of Monaco had the highest incidence and prevalence, with 10.44 per 100,000 people (95% UI: 7.80 - 14.22) and 69.02 per 100,000 people (95% UI: 52.52 - 93.39) respectively. Montenegro had the highest deaths and DALYs, specifically 4.67 per 100,000 people (95% UI: 3.61 - 6.10) and 127.61 years per 100,000 people (95% UI: 97.86 - 170.11). In contrast, the Republic of Kiribati had the lowest values for all of the above - mentioned indicators (**Figure 1**, **Supplementary Table S1**).

According to the analysis of the change trend of EAPCs from 1990 to 2021, in terms of incidence, 149 countries show a downward trend, while 55 countries show an upward trend. The Republic of Guatemala has the most significant decline in incidence (EAPC = - 3.7, 95% CI: - 3.92 - - 3.49). Regarding prevalence, 124 countries have had a significant decline, and 80 countries have no change or an increase. Among them, the Republic of Uzbekistan has the largest downward trend in prevalence (EAPC = - 3.13, 95% CI: - 3.67 - - 2.59). In terms of deaths, the number of deaths in 168 countries shows a downward trend, and in 36 countries shows an upward trend. The Republic of Korea has the largest downward trend in deaths(EAPC = - 5.65, 95% CI: - 6.01 - - 5.28). Regarding DALYs, 170 countries show a downward trend, and 34 countries show an upward trend. Among them, the Republic of Korea has the largest decline in DALYs (EAPC = - 5.95, 95% CI: - 6.29 - - 5.60)(**Supplementary Figure S2**, **Supplementary Table S1**).

### 3.4 Age-sex-time trends in the burden of LC

**Figure 2:**
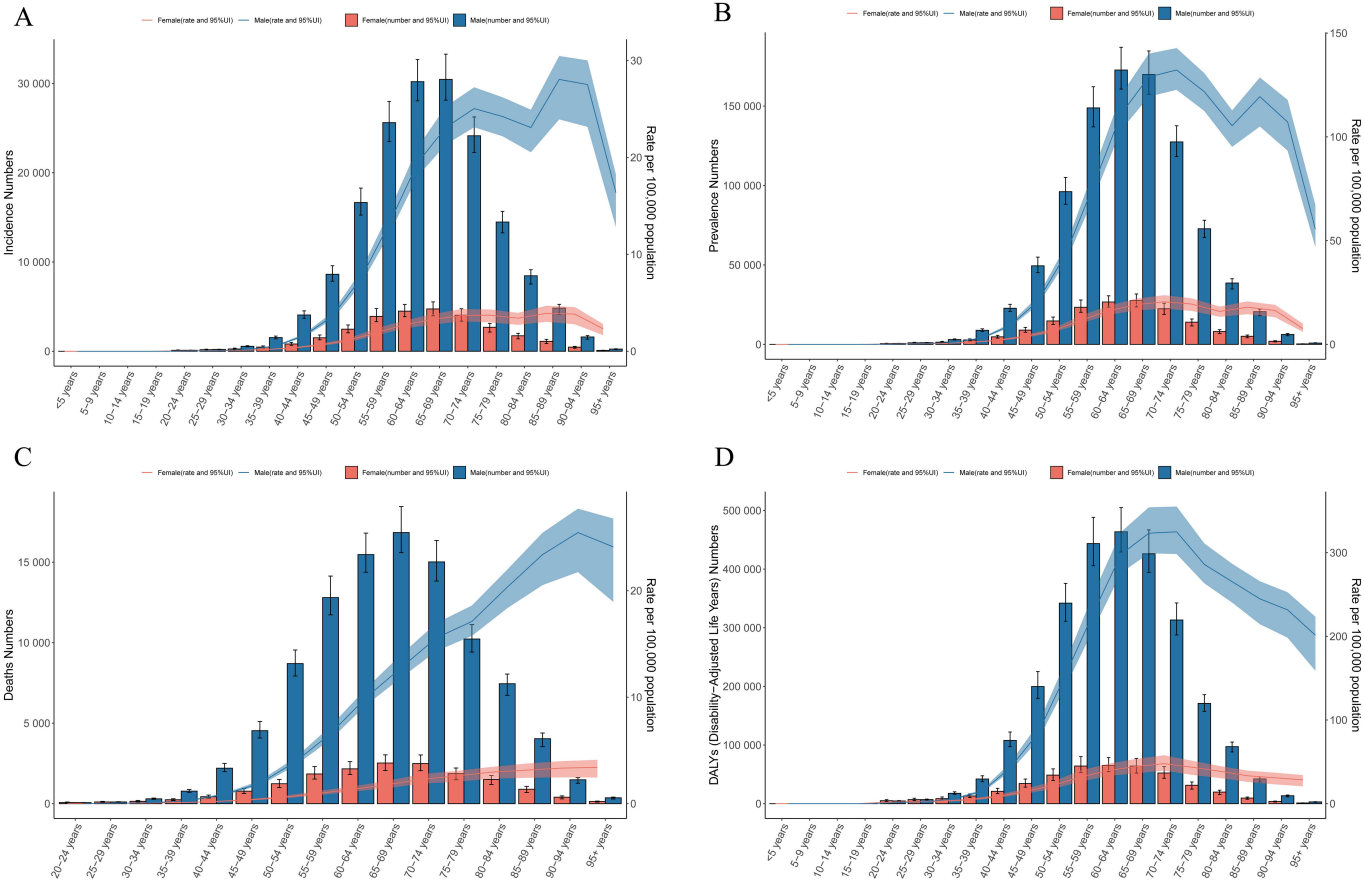
Age - sex trends in the burden of LC. **(A)** incidence. **(B)** prevalence. **(C)** deaths. **(D)** DALYs.

Age-sex correlation analyses in 2021 showed an overall increasing trend in the incidence, prevalence and DALYs of LC globally, and an overall decreasing trend after the age of 70 years, with males far outnumbering females. However, the difference between males and females initially became progressively larger with age, and this gap gradually narrowed after age 70 years (**Figures 2A, B, D**). In terms of deaths, both males and females show an upward trend, with males rising significantly faster than females; in terms of the number, the number of male deaths is higher than the number of female deaths (**Figure 2C**).

**Figure 3:**
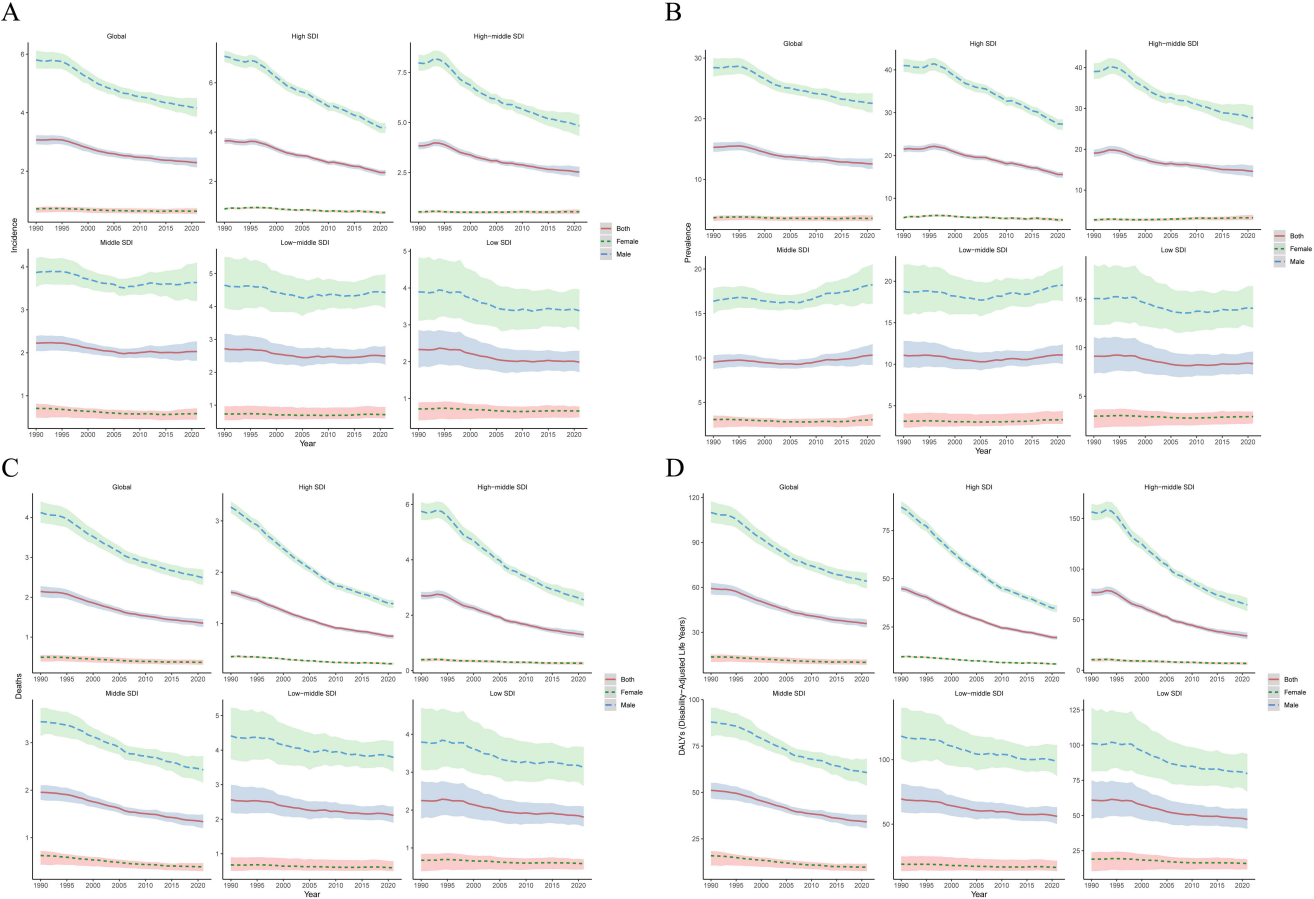
Analysis of the disease burden of LC by sex - time from 1990 to 2021. **(A)** incidence. **(B)** prevalence. **(C)** deaths. **(D)** DALYs.

The analysis of sex - time trend reveals that globally, the burden of LC has been decreasing over time for both genders. Nevertheless, in the Middle SDI, Low - middle SDI, and Low SDI regions, the incidence and prevalence have fluctuated slightly or increased. In contrast, both High SDI and High - middle SDI regions have shown a downward trend in disease burden indicators (**Figure 3**).

Age - time correlation analysis reveals that the disease burden of LC patients across all age groups globally has been on a downward trend over time. Nevertheless, since 2005, among patients of all age groups in the Middle SDI, Low - middle SDI, and Low SDI regions, there has been no difference or an increase in the variation range of the incidence and prevalence. In contrast, a downward trend has been presented in the High SDI and High - middle SDI regions. Regarding deaths and DALYs, patients of all age groups in the five SDI regions have all exhibited a downward trend over time (**Supplementary Figure S3**).

### 3.5 Results of the joinpoint regression analysis for LC

Joinpoint regression analysis showed that during the period from 1990 to 2021, the incidence, prevalence, deaths and DALYs generally showed a downward trend. For incidence, there were three year - nodes, namely 1995, 2004 and 2014. It showed an upward trend during 1990 - 1995, but it was not statistically significant; since 1995, it has shown a downward trend and was statistically significant. Prevalence also had three year - nodes, which were 1996, 2002 and 2017 respectively. It showed an upward trend from 1990–1996 and a downward trend after 1996. Deaths and DALYs also had three year - nodes respectively, namely (1994, 2007, 2014) and (1994, 2006, 2014), and all showed a downward trend in all time periods (**Supplementary Figure S4**).

### 3.6 The correlation between LC burden and SDI

**Figure 4:**
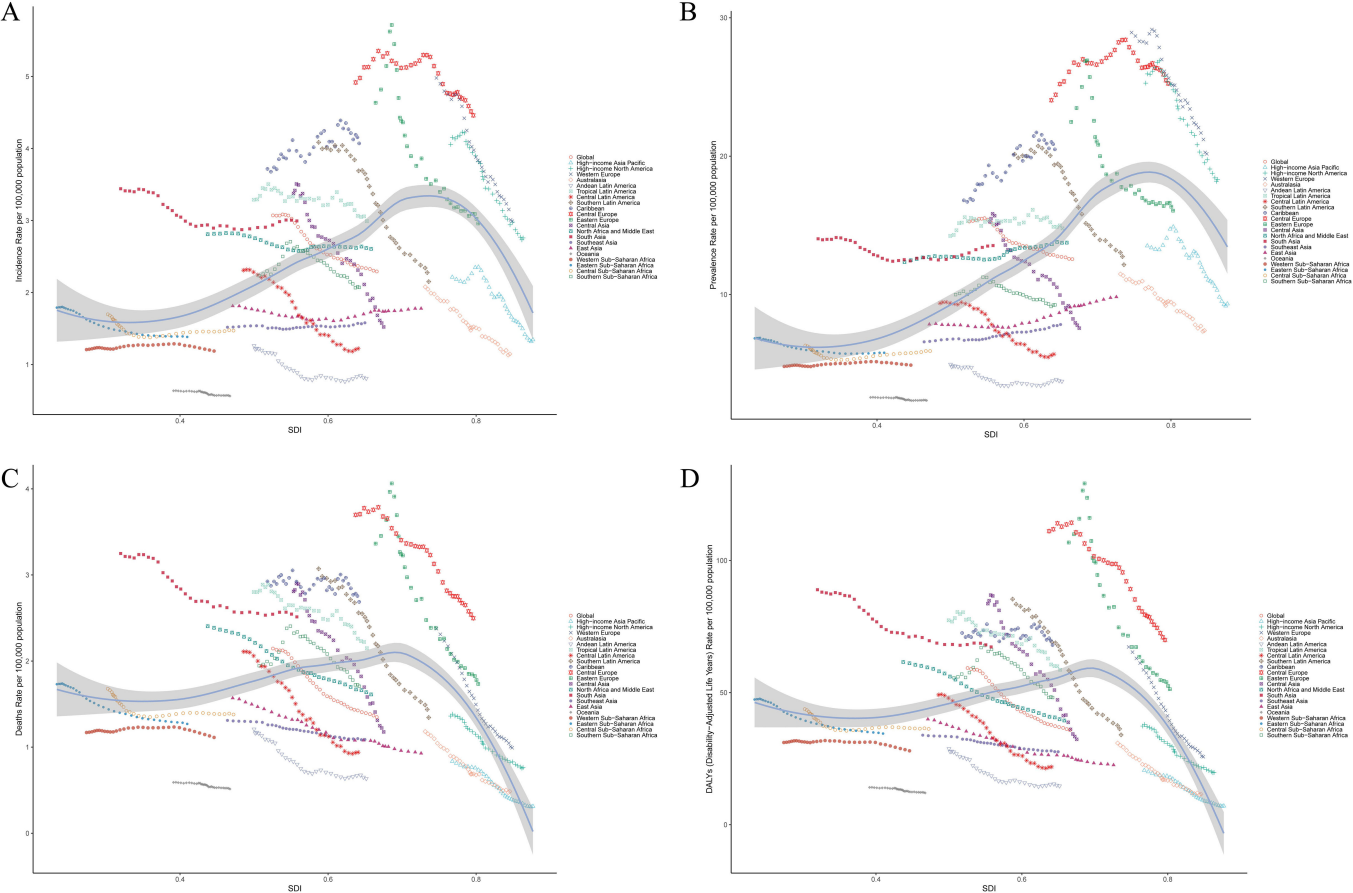
Correlation between the disease burden of LC and SDI in 21 regions. Age - standardized incidence **(A)**, prevalence **(B)**, deaths **(C)** and DALYs **(D)** (per 100,000 population) of LC in 21 regions from 1990 - 2021. Among them, the black line and gray band represent the SDI of all locations, the expected values of the incidence of each disease and their corresponding 95% CI.

**Figure 5:**
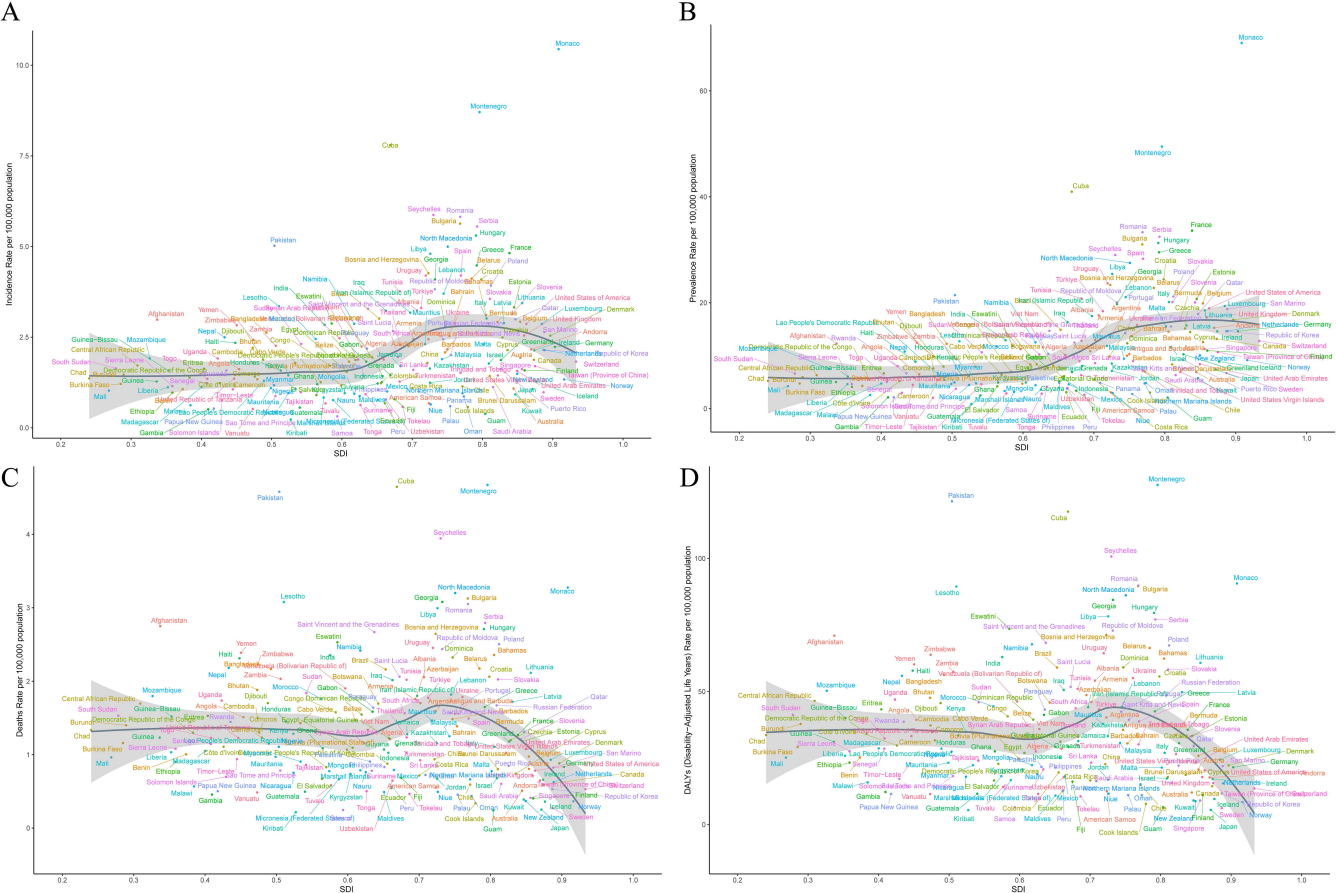
Correlation between LC and SDI disease burden in 204 countries. In 2021, the age - standardized incidence rate **(A)**, prevalence **(B)**, deaths **(C)** and DALYs **(D)** (per 100,000 people) of LC in 204 countries. Among them, the black line and gray band represent the SDI of all locations, the expected values of the incidence of each disease and their corresponding 95% CI.

Next, we analyzed the relationship between the disease burden of LC and SDI. In 21 regions, the incidence, prevalence, deaths, and DALYs had a non - linear relationship with SDI. When SDI was less than 0.7, an increase in SDI would result in an increase in the disease burden; when SDI was greater than 0.7 or less than 0.3, an increase in SDI would lead to a decrease in the disease burden. Among these regions, in High - income Asia Pacific, High - income North America, Western Europe, Eastern Europe, and Southern Latin America, the disease burden decreased the most. Most of the other regions showed a downward trend, and only a few regions showed an upward trend (**Figure 4**). In 2021, the incidence, prevalence, number of deaths, and DALYs of LC in 204 countries also exhibited a non - linear relationship with the SDI. In comparison to countries having a low SDI, those with an SDI greater than 0.7 had a lower disease burden (**Figure 5**).

### 3.7 The effects of age-period-cohort on LC

**Figure 6:**
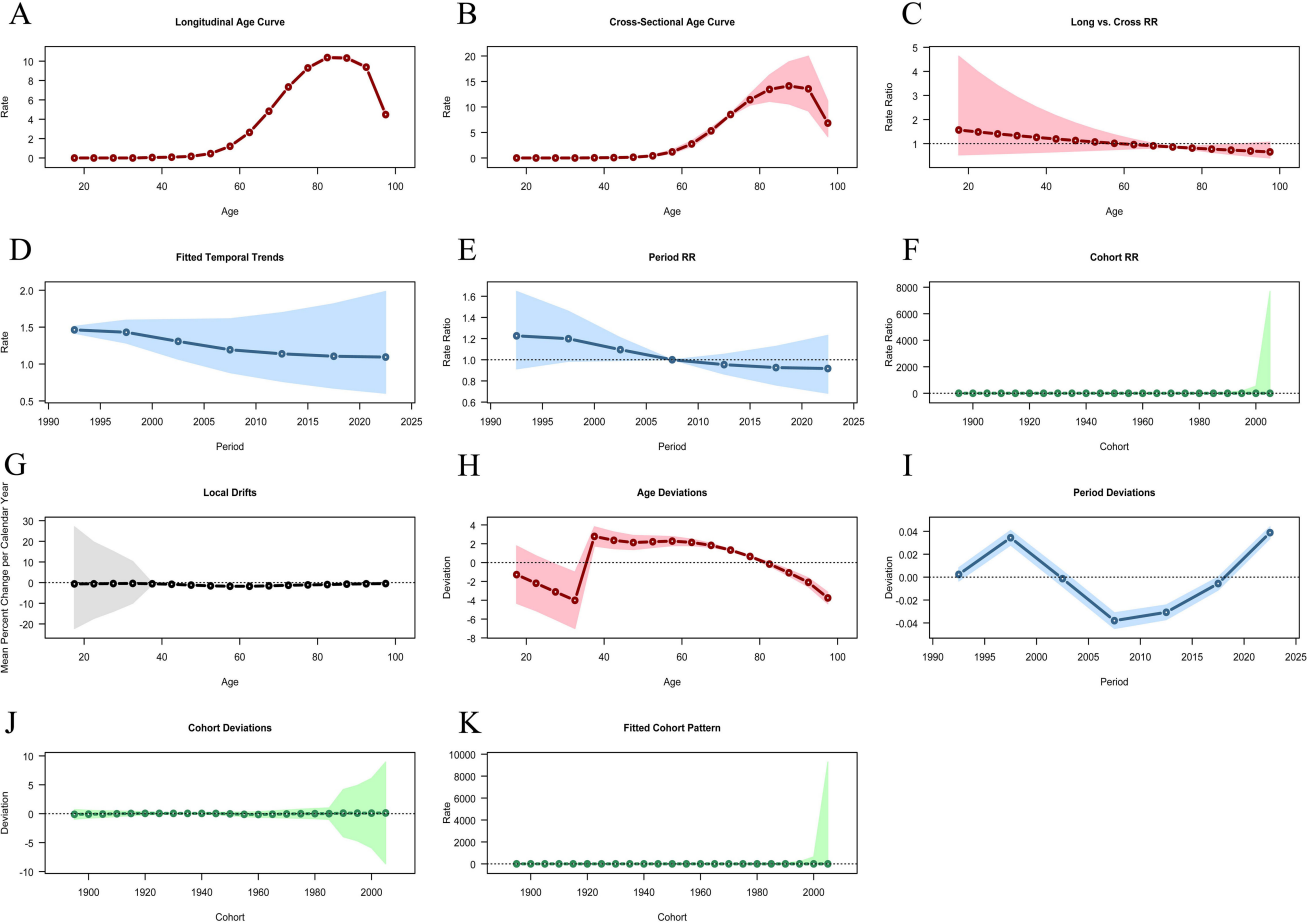
Results of age - period - cohort analysis. **(A)** Longitudinal Age Curve. **(B)** Cross-Sectional Age Curve. **(C)** Long vs. Cross RR. **(D)** Fitted Temporal Trends. **(E)** Period RR. **(F)** Cohort RR. **(G)** Local Drifts. **(H)** Age Deviations. **(I)** Period Deviations. **(J)** Cohort Deviations. **(K)** Fitted Cohort Pattern.

The age - effect curve reveals that, in terms of the longitudinal age curve (**Figure 6A**), the incidence of LC rises significantly as age increases, peaking in the 80–90 year old age group. The cross - sectional age curve (**Figure 6B**) further validates this trend, demonstrating a sharp increase in the incidence rate after 60–70 years old and remaining high in the advanced - age segment. Moreover, the long - term and cross - sectional relative risk ratios (**Figure 6C**), along with the local drift (**Figure 6G**) and age - deviation (**Figure 6H**) graphs, also show that the relative risk of LC in the elderly population is significantly higher than that in other age groups.

The period - effect analysis results reveal that the fitted temporal trends (**Figure 6D**) and the period RR (**Figure 6E**) display the LC incidence changes from 1990 to 2021. Overall, the data shows that the LC incidence has a downward trend, which may reflect the influence of factors like the progress of preventive measures and treatment methods or lifestyle changes. Moreover, the period deviation (**Figure 6I**) further verifies that there are differences in the LC incidence across different periods, indicating that environmental factors and social changes have a significant impact on the disease incidence.

Cohort effect analysis indicates that the cohort RR (**Figure 6F**) and cohort deviation (**Figure 6J**) show differences in the risk of LC incidence among different birth cohorts. The data suggest that, among those born between 1900 and 2000, the relative risk of laryngeal cancer is basically stable. However, individuals born in specific years may have different exposure risks. The fitted cohort pattern (**Figure 6K**) further demonstrates the relationship between birth cohorts and the risk of LC incidence. It shows that the incidence in some birth cohorts is significantly higher than that in others, which implies the potential influence of early - life environment and behavioral habits on the occurrence of the disease. These findings are highly significant for understanding the epidemiological characteristics of LC.

### 3.8 The decomposition analysis for LC

**Figure 7:**
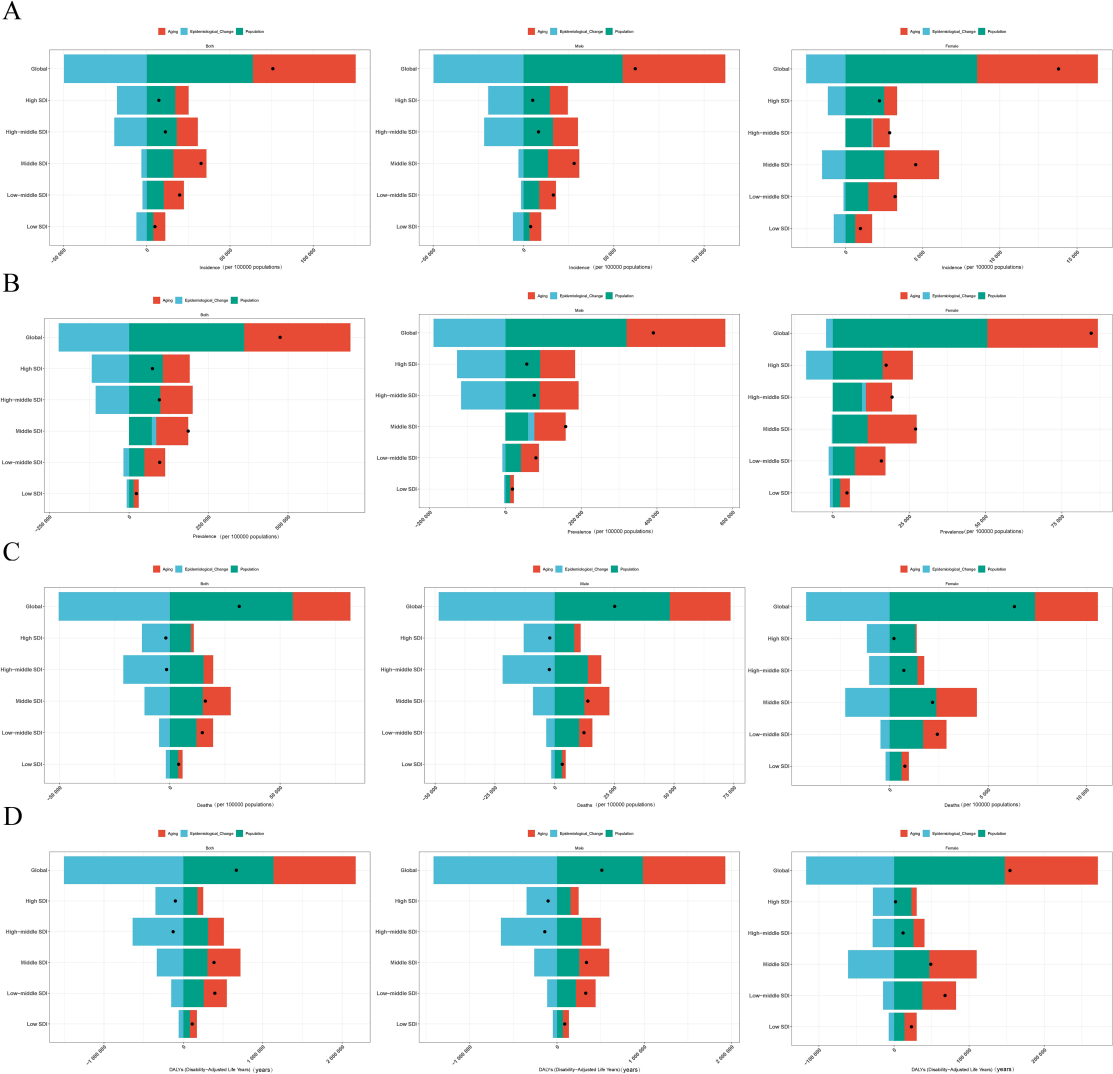
Results of decomposition analysis for LC. **(A)** incidence. **(B)** prevalence. **(C)** deaths. **(D)** DALYs.

Decomposition analysis shows that, globally and in the five SDI regions, the contribution patterns of population growth, aging, and epidemiological changes to the burden of LC are largely consistent. This is also similar among different gender groups. Overall, population growth and aging are the main factors leading to the increased burden of LC, while epidemiological changes help to reduce this burden. Specifically, globally, population growth and aging have increased the incidence rates caused by LC by 81.6% and 84.12% respectively; in contrast, epidemiological changes have reduced the incidence rate by 65.71%. The impact of population growth is particularly significant in high SDI regions (235.22%) and middle-high SDI regions (161.2%). Similarly, aging has also significantly increased the incidence in these regions, with an increase of 113.41% in middle-high SDI regions and 111.11% in high SDI regions. Epidemiological changes have greatly reduced the disease burden in all regions, with the largest decreases in middle-high SDI (- 174.61%) and high SDI (- 246.32%) regions (**Figure 7A** and **Supplementary Table S2**). Decomposition analysis of the deaths and DALYs also shows a similar trend (**Figures 7C, D** and **Supplementary Table S2**). However, in terms of prevalence, the changes in SDI regional epidemiology have increased the disease burden of LC (7.36%). Male (10.27%) also show the same trend. In the high - middle SDI regions, the changes in epidemiology have increased the LC disease burden of female (6.33%) (**Figure 7B**, **Supplementary Table S2**).

### 3.9 Prediction analysis results regarding LC

**Figure 8:**
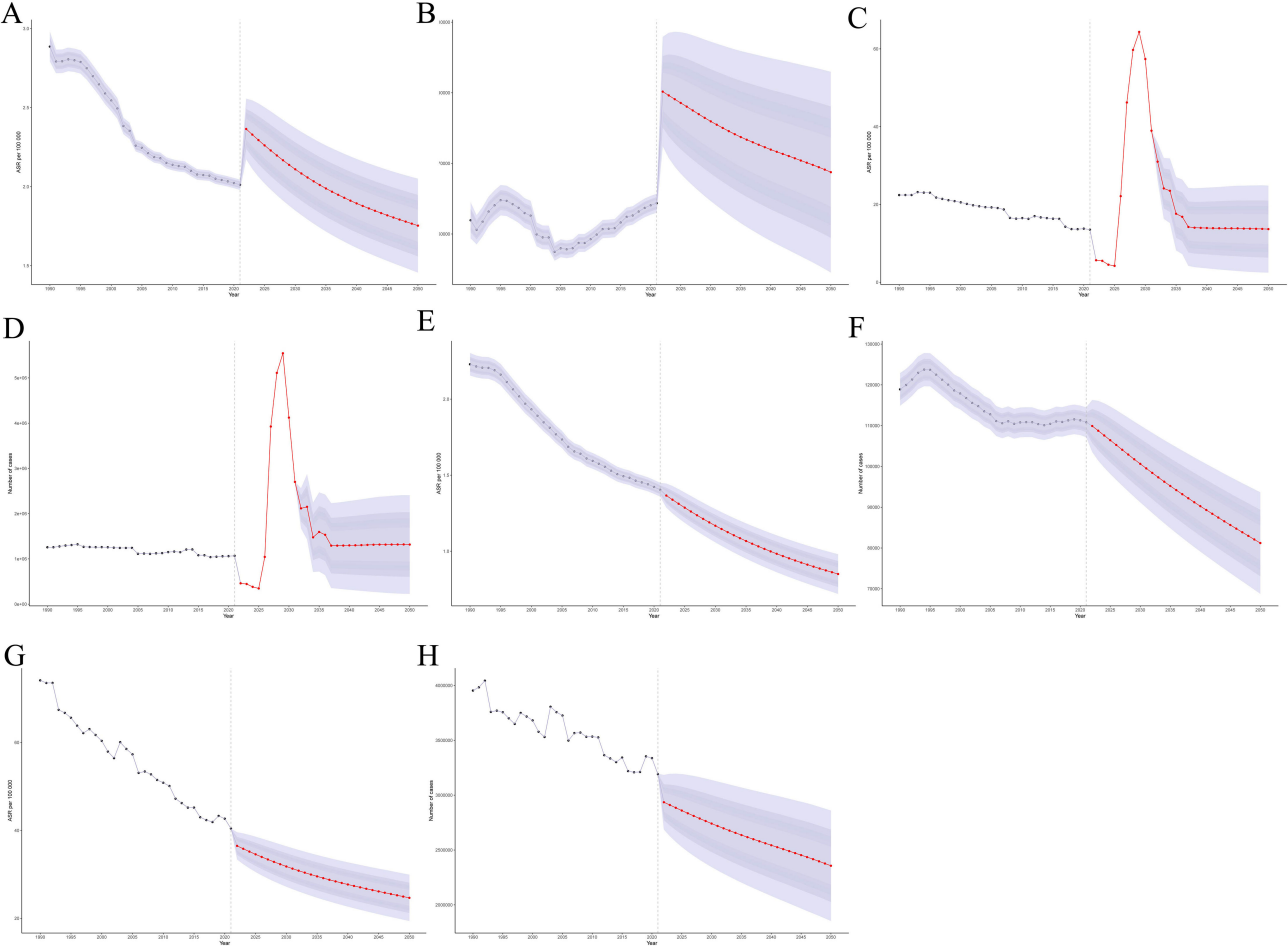
Predict the trend changes in the disease burden of LC by 2050. **(A)** Global incidence (ASR). **(B)** Global incidence (number). **(C)** Global prevalence (ASR). **(D)** Global prevalence (number). **(E)** Global deaths (ASR). **(F)** Global deaths(number). **(G)** Global DALYs (ASR). **(H)** Global DALYs (number).

Predictive analysis indicates that by 2050, the global age - standardized incidence, prevalence, number of deaths, and DALYs of LC will respectively decrease to 1.75 per 100,000 people (95% CI: 1.46 - 2.05), 13.67 per 100,000 people (95% CI: 2.5 - 24.84), 0.85 per 100,000 people (95% CI: 0.72 - 0.98), and 24.67 per 100,000 people (95% CI: 19.40 - 29.95) (**Figures 8A, C, E, G**). This implies that globally in 2050, there will be around 167,468 new LC cases (95% CI: 139,090 - 195,846), the number of patients will reach 1,300,968 (95% CI: 241,648 - 2,360,288), the number of deaths will be 81,213 (95% CI: 68,758 - 93,668), and the total number of DALYs will be 2,356,583 years (95% CI: 1,852,666 - 2,860,501) (**Figures 8B, D, F, H**). After 2021, in the short term, the incidence and prevalence will first rise and then promptly decline over time, while the deaths and DALYs generally present a downward trend (**Supplementary Tables S3, S4**). In addition, through predicting the changing trends of the global LC disease burden in different age groups, it was found that generally, its incidence, deaths and DALYs showed a gradually decreasing trend. Regarding prevalence, for the population aged 20 and above, it increased from 2030 - 2035, then decreased rapidly and tended to be stable, with a relatively small change range (**Supplementary Figures S5–8**).

### 3.10 Analysis results of LC etiology

**Figure 9:**
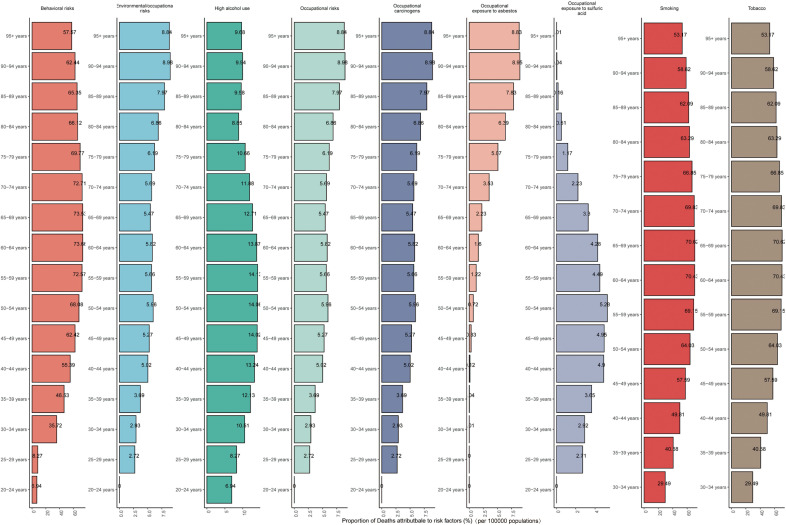
The proportional distributions of cause of LC among different age groups on a global scale(deaths).

**Figure 10:**
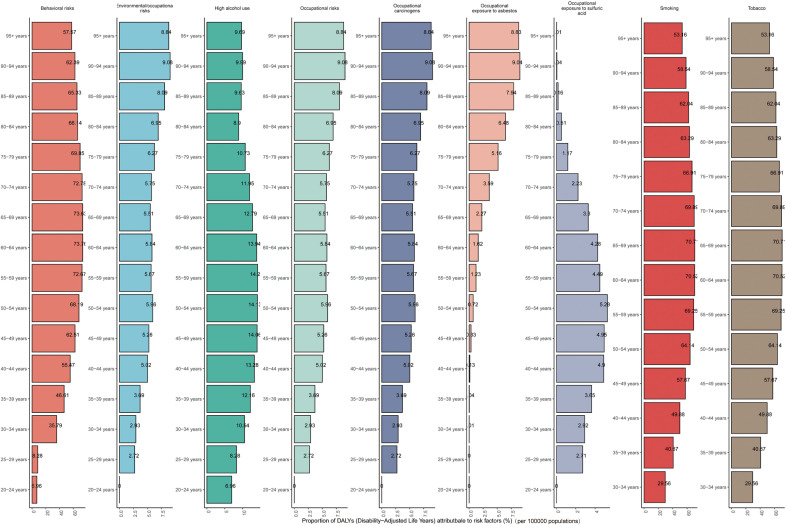
The proportional distributions of cause of LC among different age groups on a global scale (DALYs).

The purpose of risk factor analysis is to evaluate the impact of certain behavioral, environmental or metabolic factors on the overall burden of diseases. This burden is usually measured by the number of deaths caused by diseases or DALYs induced, rather than simply the incidence or prevalence of diseases. In 2021, there were nine different causes contributing to LC globally. Among them, Behavioral risks and Tobacco (Smoking) had the greatest impacts. The proportions of deaths caused by them were 69.58% and 66.23% respectively, and the proportions of DALYs were 66.87% and 65.28% respectively. Five SDI regions showed a similar distribution(**Supplementary Figure S9**, **Supplementary Table S5**). Among all age groups, behavioral risk factors (such as smoking and high alcohol consumption) account for a significant proportion of mortality factors. Among them, smoking is the main risk factor for death in all age groups, especially in the 60–69 age group, where the proportion of smoking exceeds 70%. High alcohol consumption also shows a relatively high proportion of risk factors in some age groups, reaching 14% in the 45–59 age group. Relatively speaking, the proportion of risk factors such as occupational exposure to carcinogens (such as asbestos and sulfuric acid) is lower. Overall, as age increases, the proportion of smoking as a risk factor gradually rises, while the influence of other factors is relatively stable or only has small fluctuations. The contribution of DALYs is similar to that of mortality risk factors (**Figures 9**, **10**, **Supplementary Table S6**).

## 4 Discussion

In 2021, approximately 200,883 new LC cases were reported globally, with more than 1,103,683 LC patients. The number of deaths reached 117,251, and the DALYs amounted to 3,143,308 years. The burden of LC is mainly concentrated among people aged 40 - 89, highlighting its significant impact on global public health. From 1990 to 2021, the estimated EAPC and the 95% CI of its LC incidence, prevalence, mortality, and DALYs were negative, indicating a gradually decreasing disease burden. After adjusting for age and cohort effects, the incidence of LC decreased significantly over time, which is consistent with the results of Joinpoint regression analysis, further corroborating the effectiveness of global public health interventions in reducing the burden of LC. However, despite the overall positive trend, the incidence and prevalence increased slightly from 1990–1995 and 1990–1996 respectively, and then continued to decline. The DALYs and deaths decreased significantly in 1994, 2006, 2007, and 2014, indicating that public health policies and clinical interventions in specific periods had a significant impact. It is worth noting that the significant reduction in LC burden is closely related to tobacco control, the improvement of medical services and the update of rehabilitation technologies (24, 25).To explore the factors influencing these changes, we conducted a decomposition analysis and found that population growth and aging are the main factors leading to the increase in the burden, while epidemiological changes have significantly reduced the burden. Therefore, future public health strategies should focus on addressing the increase in the spinal cord injury burden related to aging and population growth, while continuing to utilize the burden - reducing effect of epidemiological changes, especially in high SDI and high - middle SDI regions, to develop more effective prevention and intervention strategies.

According to the latest statistical data, the global incidence and deaths of LC have changed significantly over the past few decades. Specifically, since 1990, the number of new LC cases worldwide has increased by 58.7%, and the number of deaths has risen by 33.9% (26). Nevertheless, the age - standardized incidence and deaths have been decreasing year by year, with an annual decrease of 0.99% and 1.62% respectively (10, 27). This trend is common in most countries and regions. However, in some developing countries, especially in Southeast Asia and the Western Pacific region, the incidence of LC is increasing, showing significant regional differences (28).In addition, obvious inequalities exist in the LC burden in terms of gender and age. The incidence and deaths in men are considerably higher than those in women, and the burden is the most severe among the elderly (29, 30). Over the past few decades, the burden of in regions with a high SDI has changed significantly, mainly due to socioeconomic development and lifestyle changes. Research indicates that in economically developed regions, the incidence and mortality of have decreased in some respects. This is closely associated with improved living conditions, public health policies, and increased health awareness. For instance, as the smoking rate declines and drinking habits change, the incidence of has slowed down in high - SDI countries (29). In contrast, in regions with a low SDI, the decline in the burden is relatively small. This is mainly related to multiple factors. Firstly, medical resources in these areas are relatively scarce, and there are no effective screening and early diagnosis measures. Consequently, patients cannot seek medical treatment in a timely manner in the early stage of the disease and are often diagnosed at a late stage, which affects the treatment effect (26). Secondly, public health policies and cancer prevention and control measures in low - SDI regions are relatively backward, and there is a lack of specific intervention strategies for, making it difficult to significantly reduce the incidence and deaths of LC.

The burden of LC has been profoundly influenced by social development levels, exhibiting a non-linear correlation both globally and across 21 GBD regions. When the SDI falls below 0.7, an elevation in SDI correlates with a considerable rise in the burden of LC. This pattern suggests that during the nascent phases of social development, factors such as rapid urbanization, air pollution, inadequate medical resources, and erratic dietary practices have collectively exacerbated the burden of LC (31). However, once the SDI surpasses 0.7, a notable decline in this burden is observed. This reduction is especially marked in high-income regions of the Asia-Pacific, North America, Western Europe, Eastern Europe, and parts of Latin America in South America. Such a decline is intricately linked to the presence of sophisticated healthcare systems, extensive preventive strategies, and effective rehabilitation services in these areas (10). Based on the data analysis of 204 countries, the burden of LC in countries with a SDI higher than 0.7 has been alleviated, yet the overall level remains relatively high. Historically, due to economic development and industrialization, these countries once had a relatively high laryngeal cancer incidence. The high SDI regions start to decline more rapidly from a relatively high baseline level, while the low SDI regions have a smaller decline during the forecast period from 2022 to 2050. This implies that the low SDI regions may face a growth trend similar to that of the current high SDI regions in the future. Thus, it is recommended that the low SDI regions learn from the high SDI regions’ experience and take preemptive measures to reduce the future risk of LC. Overall, formulating specific intervention strategies according to the socioeconomic backgrounds of different regions is crucial for reducing the global burden of LC (32).

The incidence of LC increases significantly with age, reaching a peak at the age of 80–90 years old. It rises sharply after the age of 60–70 years old and remains at a high level. The relative risk in the elderly population is significantly higher than that in other age groups. From the perspective of period effect, the overall incidence of LC showed a downward trend from 1990 to 2021. This may be related to the progress of preventive measures, the improvement of treatment methods and the changes in lifestyle, and is also affected by environmental and social changes. The birth cohort analysis shows that the relative risk of LC in the population born between 1900 and 2000 is basically stable, but those born in specific years are exposed to different risk factors, showing the potential impact of the early living environment and behavior habits on the occurrence of the disease. In addition, it is also crucial to further explore the differences in the incidence of LC between different genders. Studies have found that the incidence of LC in men is much higher than that in women. This may be related to men being more exposed to high - risk factors such as smoking and excessive drinking. Smoking is an important risk factor for the onset of LC. A variety of harmful substances produced by tobacco combustion will directly stimulate the laryngeal mucosa, leading to the canceration of mucosal cells under long - term action. And the proportion of male smokers is significantly higher than that of female smokers, which largely explains the manifestation of gender differences in the incidence of LC (33). At the same time, occupational exposure is an important factor that cannot be ignored. For instance, those engaged in occupations like chemical engineering and asbestos processing are at relatively high risk of LC due to long - term exposure to harmful chemicals or dust (34). Moreover, there are more male workers in these occupations. Given these differences, in the prevention of LC, targeted health education should be carried out. We should advocate that men cut down on bad living habits such as smoking and excessive drinking, and strengthen the protective measures for high - risk occupational groups simultaneously, so as to lower the overall incidence rate of LC.

This study represents the inaugural attempt at a predictive analysis of the global LC burden from 2022 to 2050. The findings yield crucial insights for policymaking, long-term healthcare investments, strategic planning, and prioritization. The results indicate a general trend of gradual decline in incidence, prevalence, deaths, and DALYs associated with LC across various age demographics. This suggests that the adverse impact of global lung cancer on public health may diminish over time. However, a temporary uptick in prevalence among individuals over 20 years old is anticipated between 2030 and 2035, followed by a rapid decrease and eventual stabilization, with the magnitude of change being relatively limited. In a broader context, although certain age groups may experience a short-term increase in burden, the long-term outlook for the global LC disease burden is expected to trend downward. This reflects the potential positive impact of advancements in preventive measures and treatment modalities on future lung cancer control, providing a foundation for the development of public health strategies while underscoring the necessity for ongoing monitoring and intervention. Moreover, under current circumstances, the burden of LC in low SDI regions is projected to experience minimal change, illuminating the distinct challenges these regions and aging populations face in the prevention and treatment of lung cancer. Despite the potential for medical advancements and optimized public health strategies to alleviate the overall LC burden, the scarcity of medical resources in underdeveloped areas and the intricate health needs of the elderly may undermine the efficacy of existing preventive initiatives. Hence, public health interventions must be tailored to address specific risk factors prevalent in various age groups and regions, fostering the development of targeted prevention and response strategies to effectively mitigate disease burden and enhance long-term health outcomes for affected populations. This holistic risk management approach is instrumental in achieving more equitable health outcomes on a global scale, particularly for the most vulnerable segments of society.

## 5 Conclusions

This research establishes a comprehensive understanding of LC burdens utilizing data from the GBD 2021 database. By employing advanced statistical models including Joinpoint regression and Bayesian age-period-cohort analysis, it effectively elucidates the trends and dynamics of LC incidence, prevalence, mortality, and disability-adjusted life years across different demographics and regions from 1990 to 2021. The findings underscore the significance of age, period, and cohort effects in shaping disease patterns, while providing evidence for public health interventions. Overall, the study contributes a critical framework for monitoring and addressing the evolving challenges posed by laryngeal cancer globally.

## Data Availability

The original contributions presented in the study are included in the article/**Supplementary Material**. Further inquiries can be directed to the corresponding authors.
