# Supplementary material for: The global, regional, and national impact of laryngeal cancer from 1990 to 2021, along with forecasts for 2050: a comprehensive analysis for the Global Burden of Disease 2021 research
Source: Front Oncol. 2025 Aug 22;15:1627009. doi: 10.3389/fonc.2025.1627009 (PMC12411215; doi:10.3389/fonc.2025.1627009)
Supplement: Supplementary file 1 [file DataSheet1.docx]

**The global, regional, and national impact of the laryngeal cancer from 1990 to 2021, along with forecasts for 2050: A comprehensive analysis for the Global Burden of Disease 2021 research**

**Table S1: Age-standardized LC burden across 204 countries and regions.**

| **Location** | **Age-standardized incidence** | | | **Age-standardized prevalence** | | | **Age-standardized deaths** | | | **Age-standardized DALYs** | | |
| --- | --- | --- | --- | --- | --- | --- | --- | --- | --- | --- | --- | --- |
|  | **1990 (per 100,000 population, 95 % UI)** | **2021 (per 100,000 population, 95 % UI)** | **EAPCs (95 % CI)** | **1990 (per 100,000 population, 95 % UI)** | **2021 (per 100,000 population, 95 % UI)** | **EAPCs (95 % CI)** | **1990 (per 100,000 population, 95 % UI)** | **2021 (per 100,000 population, 95 % UI)** | **EAPCs (95 % CI)** | **1990 (per 100,000 population, 95 % UI)** | **2021 (per 100,000 population, 95 % UI)** | **EAPCs (95 % CI)** |
| **American Samoa** | 1.32(1.01,1.69) | 0.68(0.53,0.87) | -2.83(-3.15,-2.50) | 5.49(4.26,6.88) | 3.08(2.49,3.86) | -2.46(-2.75,-2.17) | 1.14(0.87,1.45) | 0.55(0.43,0.70) | -2.93(-3.25,-2.62) | 26.37(19.86,33.50) | 12.57(10.00,15.91) | -3.01(-3.33,-2.69) |
| **Antigua and Barbuda** | 2.19(1.99,2.39) | 2.17(1.94,2.44) | -0.02(-0.35,0.30) | 9.83(8.94,10.68) | 10.31(9.24,11.61) | 0.22(-0.06,0.50) | 1.76(1.60,1.90) | 1.56(1.39,1.74) | -0.36(-0.70,-0.02) | 44.91(40.91,48.58) | 37.46(33.55,42.31) | -0.53(-0.83,-0.22) |
| **Arab Republic of Egypt** | 1.28(1.09,1.64) | 1.54(1.21,1.95) | 0.86(0.66,1.06) | 5.74(4.92,7.13) | 7.67(6.12,9.54) | 1.10(0.95,1.25) | 1.16(0.98,1.44) | 1.09(0.86,1.37) | 0.19(-0.04,0.42) | 29.76(25.15,37.62) | 26.93(20.83,34.42) | -0.02(-0.24,0.20) |
| **Argentine Republic** | 4.44(4.07,4.86) | 2.44(2.19,2.69) | -2.04(-2.25,-1.82) | 21.78(19.88,23.76) | 13.54(12.30,14.89) | -1.70(-1.94,-1.46) | 3.37(3.11,3.68) | 1.59(1.44,1.74) | -2.41(-2.59,-2.23) | 94.91(87.11,103.87) | 40.28(36.34,44.57) | -2.84(-3.03,-2.64) |
| **Australia** | 1.96(1.77,2.19) | 1.08(0.96,1.22) | -1.89(-2.02,-1.76) | 10.52(9.45,11.68) | 6.90(6.09,7.64) | -1.31(-1.47,-1.16) | 1.24(1.13,1.37) | 0.51(0.45,0.57) | -2.97(-3.07,-2.86) | 32.55(29.35,36.14) | 12.05(10.65,13.56) | -3.25(-3.36,-3.15) |
| **Barbados** | 1.80(1.66,1.97) | 2.08(1.63,2.65) | 0.45(0.27,0.63) | 8.27(7.64,8.96) | 10.01(7.84,12.71) | 0.67(0.50,0.84) | 1.43(1.31,1.56) | 1.48(1.17,1.86) | 0.10(-0.09,0.29) | 36.04(33.15,39.18) | 35.58(27.68,45.31) | -0.00(-0.18,0.17) |
| **Belize** | 1.44(1.33,1.54) | 1.82(1.58,2.14) | 0.94(0.41,1.48) | 6.36(5.91,6.85) | 8.35(7.28,9.76) | 1.12(0.67,1.57) | 1.24(1.15,1.33) | 1.47(1.27,1.70) | 0.66(0.08,1.24) | 32.01(29.68,34.43) | 37.28(31.92,43.41) | 0.67(0.10,1.24) |
| **Bermuda** | 3.35(3.05,3.70) | 2.92(2.40,3.64) | -0.11(-0.31,0.10) | 15.54(14.07,17.13) | 16.69(13.70,20.73) | 0.51(0.30,0.72) | 2.36(2.16,2.58) | 1.36(1.12,1.69) | -1.44(-1.65,-1.23) | 59.14(53.82,65.31) | 33.63(27.19,41.97) | -1.54(-1.75,-1.33) |
| **Bolivarian Republic of Venezuela** | 3.07(2.89,3.24) | 2.47(1.88,3.20) | -1.25(-1.45,-1.05) | 12.66(11.99,13.30) | 11.25(8.60,14.57) | -0.89(-1.10,-0.68) | 2.72(2.57,2.87) | 1.90(1.45,2.45) | -1.72(-1.93,-1.51) | 66.30(62.80,69.66) | 46.26(34.75,59.96) | -1.78(-2.01,-1.56) |
| **Bosnia and Herzegovina** | 4.95(4.23,5.75) | 4.27(3.04,5.52) | -0.46(-0.57,-0.36) | 23.69(20.38,27.33) | 23.35(17.06,29.75) | 0.09(-0.01,0.19) | 3.95(3.38,4.58) | 2.64(1.88,3.42) | -1.44(-1.59,-1.30) | 112.29(96.14,130.71) | 71.25(50.45,92.44) | -1.63(-1.78,-1.48) |
| **Brunei Darussalam** | 2.92(2.28,3.67) | 1.43(1.12,1.83) | -2.34(-2.49,-2.19) | 14.17(11.51,17.37) | 8.37(6.72,10.48) | -1.84(-1.96,-1.73) | 2.08(1.62,2.62) | 0.80(0.63,1.02) | -2.99(-3.19,-2.80) | 47.15(36.77,59.40) | 17.89(13.98,22.99) | -3.14(-3.33,-2.95) |
| **Burkina Faso** | 0.99(0.74,1.26) | 1.21(0.86,1.63) | 1.04(0.88,1.20) | 3.84(2.93,4.90) | 4.75(3.39,6.32) | 1.04(0.88,1.21) | 0.96(0.73,1.23) | 1.16(0.83,1.56) | 1.00(0.84,1.16) | 25.03(18.53,32.65) | 29.16(20.20,39.87) | 0.86(0.70,1.02) |
| **Canada** | 3.92(3.57,4.27) | 1.88(1.67,2.10) | -2.24(-2.42,-2.06) | 24.25(22.08,26.41) | 13.16(11.87,14.62) | -1.88(-2.08,-1.68) | 1.50(1.38,1.62) | 0.59(0.53,0.65) | -2.88(-3.00,-2.77) | 39.92(36.62,43.24) | 14.45(12.90,16.16) | -3.17(-3.28,-3.06) |
| **Central African Republic** | 2.18(1.38,2.94) | 1.72(1.09,2.44) | -0.81(-0.87,-0.76) | 7.64(4.85,10.31) | 6.32(4.08,8.88) | -0.65(-0.72,-0.59) | 2.16(1.40,2.89) | 1.70(1.10,2.38) | -0.82(-0.87,-0.77) | 59.15(35.87,81.56) | 46.50(28.43,67.22) | -0.84(-0.90,-0.79) |
| **Commonwealth of Dominica** | 2.48(2.09,2.96) | 2.80(2.13,3.62) | 0.47(0.41,0.54) | 10.32(8.79,12.19) | 12.35(9.50,15.97) | 0.67(0.62,0.72) | 2.19(1.83,2.62) | 2.32(1.75,3.00) | 0.26(0.19,0.34) | 53.57(45.17,63.88) | 59.07(44.46,76.51) | 0.46(0.38,0.53) |
| **Commonwealth of the Bahamas** | 3.16(2.86,3.47) | 3.11(2.48,3.86) | 0.22(0.08,0.36) | 14.01(12.71,15.35) | 14.55(11.60,18.10) | 0.43(0.29,0.57) | 2.55(2.32,2.78) | 2.36(1.89,2.92) | -0.05(-0.19,0.09) | 71.04(64.15,78.30) | 62.37(49.65,77.98) | -0.20(-0.35,-0.05) |
| **Cook Islands** | 0.70(0.55,0.85) | 0.57(0.44,0.78) | -0.80(-1.22,-0.38) | 3.07(2.42,3.77) | 3.05(2.38,4.11) | -0.17(-0.55,0.22) | 0.56(0.44,0.68) | 0.34(0.26,0.46) | -1.79(-2.20,-1.36) | 12.61(9.81,15.60) | 7.80(5.98,10.75) | -1.67(-2.05,-1.28) |
| **Czech Republic** | 3.68(3.18,4.23) | 2.49(1.98,3.10) | -1.14(-1.31,-0.97) | 18.45(16.03,21.16) | 15.14(12.26,18.50) | -0.57(-0.78,-0.36) | 2.60(2.27,2.98) | 1.20(0.95,1.49) | -2.34(-2.45,-2.23) | 77.46(67.37,88.74) | 32.96(26.05,41.13) | -2.62(-2.73,-2.51) |
| **Democratic People's Republic of Korea** | 1.23(0.89,1.62) | 1.26(0.89,1.67) | 0.16(0.09,0.23) | 5.60(4.11,7.40) | 6.40(4.54,8.44) | 0.60(0.49,0.72) | 0.98(0.71,1.29) | 0.83(0.60,1.09) | -0.51(-0.57,-0.45) | 26.80(19.10,36.06) | 22.83(15.75,30.45) | -0.53(-0.59,-0.46) |
| **Democratic Republic of Sao Tome and Principe** | 0.69(0.54,0.87) | 0.88(0.70,1.13) | 0.94(0.85,1.03) | 2.94(2.35,3.60) | 3.77(3.09,4.75) | 0.89(0.82,0.95) | 0.67(0.52,0.85) | 0.78(0.62,1.00) | 0.74(0.61,0.87) | 16.16(12.42,20.29) | 18.22(14.46,23.62) | 0.53(0.42,0.63) |
| **Democratic Republic of the Congo** | 1.55(1.11,2.05) | 1.38(0.97,1.85) | -0.36(-0.56,-0.17) | 5.83(4.20,7.69) | 5.58(3.99,7.43) | -0.13(-0.34,0.09) | 1.54(1.11,2.01) | 1.30(0.91,1.72) | -0.52(-0.69,-0.35) | 39.38(27.77,52.82) | 34.23(23.70,45.80) | -0.43(-0.58,-0.27) |
| **Democratic Republic of Timor-Leste** | 1.10(0.77,1.54) | 1.06(0.75,1.51) | 0.07(-0.18,0.32) | 4.42(3.11,6.11) | 4.51(3.21,6.28) | 0.27(0.02,0.52) | 1.05(0.75,1.47) | 0.94(0.67,1.31) | -0.26(-0.48,-0.03) | 26.54(18.33,37.84) | 23.00(15.98,32.85) | -0.35(-0.61,-0.09) |
| **Democratic Socialist Republic of Sri Lanka** | 1.11(0.90,1.40) | 1.60(0.94,2.41) | 2.35(1.88,2.83) | 5.02(4.08,6.25) | 8.60(5.22,12.95) | 2.88(2.43,3.33) | 0.93(0.75,1.16) | 0.92(0.56,1.38) | 1.12(0.65,1.59) | 22.85(18.48,28.95) | 23.29(13.70,35.50) | 1.10(0.65,1.55) |
| **Dominican Republic** | 1.77(1.41,2.23) | 1.87(1.35,2.51) | 0.47(0.33,0.61) | 7.42(5.98,9.37) | 8.37(6.08,11.18) | 0.59(0.47,0.72) | 1.65(1.31,2.07) | 1.55(1.12,2.07) | 0.14(0.00,0.28) | 40.62(32.35,51.59) | 39.42(27.94,53.40) | 0.24(0.12,0.36) |
| **Eastern Republic of Uruguay** | 6.86(6.32,7.46) | 4.20(3.77,4.70) | -1.67(-1.80,-1.55) | 34.08(31.38,37.14) | 23.65(21.38,26.26) | -1.31(-1.42,-1.19) | 4.82(4.47,5.21) | 2.45(2.21,2.74) | -2.22(-2.34,-2.09) | 132.95(122.51,144.48) | 64.39(58.36,72.19) | -2.40(-2.54,-2.26) |
| **Federal Democratic Republic of Ethiopia** | 1.59(0.91,2.29) | 0.97(0.69,1.31) | -1.91(-2.10,-1.73) | 5.74(3.44,8.10) | 4.12(3.04,5.47) | -1.38(-1.54,-1.22) | 1.58(0.91,2.27) | 0.90(0.65,1.20) | -2.11(-2.26,-1.95) | 43.39(24.01,62.62) | 23.31(16.51,31.76) | -2.33(-2.51,-2.16) |
| **Federal Democratic Republic of Nepal** | 3.06(2.11,4.26) | 2.49(1.82,3.46) | -0.63(-0.92,-0.34) | 12.02(8.46,16.52) | 10.79(8.03,14.70) | -0.37(-0.62,-0.11) | 2.96(2.05,4.11) | 2.18(1.60,3.05) | -0.93(-1.21,-0.66) | 79.08(54.02,110.21) | 55.92(40.68,78.51) | -1.09(-1.36,-0.81) |
| **Federal Republic of Germany** | 3.06(2.80,3.35) | 2.23(1.99,2.48) | -1.29(-1.44,-1.13) | 17.49(15.99,19.17) | 14.64(13.31,16.14) | -0.87(-1.09,-0.66) | 1.62(1.50,1.75) | 0.83(0.75,0.92) | -2.22(-2.33,-2.11) | 48.35(44.36,52.50) | 21.54(19.38,23.80) | -2.73(-2.83,-2.64) |
| **Federal Republic of Nigeria** | 1.41(0.99,1.90) | 1.18(0.83,1.59) | -0.58(-0.72,-0.44) | 5.66(4.10,7.51) | 4.94(3.51,6.63) | -0.42(-0.54,-0.31) | 1.37(0.97,1.83) | 1.11(0.79,1.47) | -0.65(-0.81,-0.48) | 36.96(25.43,50.22) | 27.43(18.89,37.47) | -0.99(-1.15,-0.83) |
| **Federal Republic of Somalia** | 2.19(1.43,3.12) | 1.65(1.03,2.34) | -0.98(-1.04,-0.93) | 8.16(5.50,11.48) | 6.49(4.23,9.12) | -0.80(-0.85,-0.75) | 2.13(1.39,3.04) | 1.60(1.01,2.27) | -0.99(-1.04,-0.94) | 60.17(38.98,87.39) | 44.96(27.96,66.07) | -1.03(-1.10,-0.97) |
| **Federated States of Micronesia** | 0.87(0.65,1.16) | 0.76(0.56,1.05) | -0.53(-0.59,-0.47) | 3.31(2.54,4.38) | 3.17(2.35,4.28) | -0.22(-0.26,-0.17) | 0.82(0.62,1.10) | 0.67(0.49,0.91) | -0.83(-0.91,-0.76) | 20.67(15.47,27.95) | 16.72(12.11,23.00) | -0.82(-0.88,-0.76) |
| **Federative Republic of Brazil** | 3.33(3.19,3.47) | 3.01(2.81,3.19) | -0.34(-0.44,-0.25) | 14.42(13.81,14.99) | 14.89(13.96,15.77) | 0.05(-0.05,0.15) | 2.84(2.72,2.96) | 2.16(2.02,2.29) | -0.84(-0.94,-0.75) | 78.39(75.26,81.46) | 59.02(55.45,62.39) | -0.94(-1.05,-0.83) |
| **French Republic** | 8.79(7.98,9.66) | 4.82(4.22,5.44) | -1.61(-1.97,-1.26) | 51.53(46.72,56.62) | 33.62(29.92,37.59) | -1.07(-1.34,-0.80) | 3.91(3.61,4.26) | 1.27(1.12,1.42) | -3.49(-3.88,-3.09) | 116.39(107.34,127.45) | 35.58(31.53,39.79) | -3.68(-4.06,-3.30) |
| **Gabonese Republic** | 2.11(1.56,2.76) | 1.82(1.32,2.44) | -0.53(-0.58,-0.48) | 8.09(6.09,10.55) | 7.70(5.60,10.14) | -0.21(-0.26,-0.15) | 2.01(1.49,2.63) | 1.59(1.15,2.10) | -0.82(-0.87,-0.77) | 53.08(38.99,70.33) | 41.24(29.28,54.99) | -0.87(-0.93,-0.81) |
| **Georgia** | 5.12(4.70,5.56) | 4.09(3.52,4.70) | -0.34(-0.65,-0.03) | 23.72(21.80,25.71) | 19.77(17.25,22.60) | -0.34(-0.61,-0.06) | 3.96(3.65,4.29) | 3.08(2.66,3.51) | -0.31(-0.63,0.00) | 118.00(108.32,128.20) | 84.43(73.11,96.63) | -0.63(-0.93,-0.34) |
| **Grand Duchy of Luxembourg** | 5.30(4.84,5.77) | 2.86(2.53,3.23) | -1.88(-2.02,-1.73) | 30.15(27.56,32.94) | 19.38(17.24,21.64) | -1.34(-1.50,-1.19) | 2.60(2.40,2.80) | 0.93(0.83,1.04) | -3.25(-3.44,-3.05) | 73.11(67.39,78.76) | 24.37(21.74,27.18) | -3.49(-3.66,-3.32) |
| **Greenland** | 3.33(2.68,4.05) | 1.87(1.46,2.43) | -1.41(-1.66,-1.15) | 15.62(12.72,18.73) | 10.26(8.29,12.89) | -0.95(-1.16,-0.75) | 2.51(2.02,3.07) | 1.18(0.92,1.54) | -2.06(-2.28,-1.83) | 71.12(57.35,86.59) | 30.62(23.97,39.71) | -2.35(-2.56,-2.13) |
| **Grenada** | 1.74(1.55,1.97) | 1.45(1.24,1.69) | -0.21(-0.77,0.36) | 7.73(6.86,8.69) | 6.93(5.96,8.01) | 0.03(-0.40,0.47) | 1.52(1.35,1.72) | 1.13(0.96,1.31) | -0.56(-1.17,0.06) | 40.20(35.30,45.60) | 29.27(24.86,34.46) | -0.49(-1.00,0.03) |
| **Guam** | 0.77(0.64,0.89) | 0.39(0.33,0.45) | -2.24(-2.65,-1.83) | 3.62(3.07,4.16) | 2.09(1.81,2.42) | -1.73(-2.16,-1.29) | 0.56(0.46,0.65) | 0.24(0.21,0.28) | -2.75(-3.07,-2.43) | 12.91(10.83,14.81) | 6.50(5.57,7.57) | -2.30(-2.61,-2.00) |
| **Hashemite Kingdom of Jordan** | 1.93(1.45,2.48) | 1.25(0.88,1.74) | -1.86(-2.14,-1.58) | 9.08(6.95,11.47) | 7.14(5.18,9.77) | -1.12(-1.39,-0.84) | 1.51(1.14,1.95) | 0.67(0.48,0.93) | -3.25(-3.58,-2.91) | 39.14(29.70,50.35) | 16.90(11.99,23.55) | -3.36(-3.71,-3.01) |
| **Hellenic Republic** | 6.12(5.71,6.52) | 4.48(4.05,4.87) | -0.97(-1.07,-0.87) | 35.92(33.50,38.39) | 29.53(26.91,31.90) | -0.62(-0.74,-0.50) | 2.74(2.57,2.88) | 1.65(1.51,1.78) | -1.56(-1.65,-1.47) | 67.62(63.90,71.32) | 42.53(39.05,46.14) | -1.42(-1.50,-1.33) |
| **Hungary** | 6.13(5.48,6.86) | 5.30(4.36,6.44) | -0.95(-1.17,-0.73) | 30.11(26.97,33.34) | 31.28(26.02,37.43) | -0.36(-0.61,-0.10) | 4.39(3.92,4.87) | 2.71(2.25,3.23) | -1.94(-2.09,-1.79) | 136.85(121.68,151.96) | 79.54(65.41,96.01) | -2.24(-2.44,-2.04) |
| **Independent State of Papua New Guinea** | 0.58(0.38,0.83) | 0.53(0.37,0.77) | -0.32(-0.38,-0.26) | 2.27(1.55,3.17) | 2.15(1.54,3.04) | -0.26(-0.33,-0.18) | 0.55(0.37,0.80) | 0.50(0.35,0.74) | -0.32(-0.36,-0.27) | 13.07(8.61,18.69) | 11.53(7.97,16.78) | -0.45(-0.51,-0.39) |
| **Independent State of Samoa** | 0.33(0.26,0.43) | 0.32(0.25,0.42) | -0.26(-0.36,-0.17) | 1.46(1.14,1.89) | 1.52(1.19,2.00) | 0.00(-0.09,0.09) | 0.28(0.22,0.36) | 0.25(0.19,0.33) | -0.55(-0.63,-0.46) | 7.49(5.73,9.74) | 6.52(5.03,8.82) | -0.52(-0.62,-0.41) |
| **Ireland** | 3.65(3.31,4.05) | 2.14(1.85,2.45) | -1.19(-1.37,-1.00) | 20.88(18.89,23.18) | 14.61(12.76,16.69) | -0.62(-0.80,-0.44) | 1.80(1.65,1.97) | 0.66(0.58,0.74) | -2.94(-3.10,-2.78) | 47.44(43.29,52.17) | 17.15(15.09,19.54) | -2.95(-3.11,-2.80) |
| **Islamic Republic of Afghanistan** | 3.35(1.84,5.10) | 2.98(1.69,4.34) | -0.48(-0.57,-0.39) | 12.72(7.20,19.37) | 12.27(7.29,17.88) | -0.23(-0.29,-0.17) | 3.29(1.85,4.93) | 2.75(1.58,3.94) | -0.67(-0.79,-0.55) | 85.58(45.62,135.35) | 70.97(38.82,108.03) | -0.74(-0.86,-0.61) |
| **Islamic Republic of Iran** | 3.29(2.75,3.75) | 3.18(2.82,3.58) | -0.04(-0.16,0.09) | 14.91(12.65,16.93) | 17.48(15.72,19.78) | 0.54(0.41,0.68) | 2.70(2.24,3.10) | 1.80(1.59,2.00) | -1.17(-1.24,-1.09) | 68.26(56.86,77.64) | 44.15(39.28,49.52) | -1.25(-1.34,-1.17) |
| **Islamic Republic of Mauritania** | 0.95(0.74,1.19) | 1.09(0.77,1.55) | 0.63(0.39,0.88) | 3.79(3.02,4.76) | 4.68(3.30,6.59) | 0.81(0.58,1.04) | 0.92(0.72,1.15) | 0.97(0.68,1.35) | 0.42(0.19,0.65) | 24.01(18.80,30.31) | 23.24(16.11,33.01) | 0.07(-0.15,0.30) |
| **Islamic Republic of Pakistan** | 5.25(4.23,6.49) | 5.02(3.67,6.65) | -0.45(-0.67,-0.23) | 21.28(17.44,25.87) | 21.43(16.16,27.97) | -0.25(-0.43,-0.08) | 5.04(4.05,6.25) | 4.58(3.35,6.04) | -0.61(-0.86,-0.36) | 132.92(107.23,163.79) | 121.47(88.73,161.68) | -0.61(-0.87,-0.35) |
| **Jamaica** | 1.45(1.29,1.62) | 1.70(1.25,2.31) | 0.59(0.13,1.05) | 6.63(5.92,7.40) | 8.07(5.94,10.84) | 0.65(0.22,1.09) | 1.17(1.04,1.30) | 1.28(0.93,1.70) | 0.40(-0.04,0.84) | 29.29(26.09,32.69) | 31.40(22.62,41.93) | 0.25(-0.23,0.74) |
| **Japan** | 1.87(1.77,1.96) | 1.07(0.99,1.13) | -1.86(-2.11,-1.61) | 11.92(11.28,12.63) | 7.42(6.90,7.85) | -1.55(-1.81,-1.29) | 0.58(0.55,0.60) | 0.25(0.23,0.27) | -2.92(-3.06,-2.77) | 13.51(12.95,14.04) | 5.51(5.13,5.87) | -3.05(-3.24,-2.87) |
| **Kingdom of Bahrain** | 3.80(3.07,4.83) | 2.53(1.85,3.51) | -2.01(-2.27,-1.76) | 16.17(13.33,20.40) | 13.73(10.29,19.01) | -1.09(-1.29,-0.89) | 3.09(2.49,3.89) | 1.39(1.02,1.91) | -3.35(-3.64,-3.06) | 70.98(57.68,89.71) | 30.51(22.02,43.15) | -3.51(-3.79,-3.22) |
| **Kingdom of Belgium** | 6.09(5.56,6.63) | 2.92(2.62,3.26) | -2.46(-2.60,-2.32) | 35.43(32.28,38.62) | 19.87(17.97,22.01) | -2.00(-2.13,-1.86) | 2.74(2.54,2.96) | 0.97(0.87,1.08) | -3.37(-3.49,-3.25) | 76.97(71.24,83.03) | 25.88(23.47,28.47) | -3.65(-3.76,-3.53) |
| **Kingdom of Bhutan** | 2.59(1.69,3.74) | 2.21(1.52,3.05) | -0.53(-0.67,-0.40) | 10.48(7.08,14.86) | 9.90(7.01,13.53) | -0.22(-0.36,-0.08) | 2.48(1.64,3.60) | 1.88(1.31,2.60) | -0.91(-1.01,-0.81) | 67.45(43.78,97.39) | 47.62(32.68,66.34) | -1.16(-1.27,-1.06) |
| **Kingdom of Cambodia** | 1.89(1.43,2.51) | 1.78(1.24,2.66) | -0.26(-0.44,-0.08) | 7.22(5.51,9.54) | 7.61(5.42,11.28) | 0.10(-0.08,0.29) | 1.81(1.38,2.40) | 1.50(1.06,2.21) | -0.69(-0.83,-0.55) | 46.81(34.75,62.47) | 37.26(25.97,56.20) | -0.86(-1.01,-0.71) |
| **Kingdom of Denmark** | 4.16(3.79,4.55) | 2.54(2.29,2.80) | -1.61(-1.73,-1.48) | 24.22(22.02,26.34) | 16.79(15.22,18.34) | -1.15(-1.27,-1.04) | 1.95(1.79,2.11) | 0.88(0.80,0.98) | -2.79(-2.94,-2.65) | 53.50(48.81,58.31) | 22.26(20.21,24.45) | -3.02(-3.15,-2.89) |
| **Kingdom of Eswatini** | 3.04(1.97,4.12) | 2.88(1.74,4.15) | 0.06(-0.35,0.47) | 12.23(8.15,16.45) | 11.84(7.42,16.93) | 0.03(-0.20,0.25) | 2.79(1.79,3.76) | 2.53(1.53,3.64) | -0.04(-0.48,0.41) | 78.12(49.86,108.09) | 73.54(43.25,107.77) | 0.06(-0.43,0.54) |
| **Kingdom of Lesotho** | 2.15(1.65,2.80) | 3.35(2.29,4.55) | 1.88(1.56,2.19) | 8.70(6.72,11.03) | 12.73(8.80,17.13) | 1.59(1.38,1.79) | 2.02(1.53,2.60) | 3.08(2.11,4.15) | 1.84(1.51,2.17) | 55.01(41.84,71.08) | 89.44(59.67,123.47) | 2.04(1.66,2.43) |
| **Kingdom of Morocco** | 2.24(1.72,2.78) | 2.45(1.71,3.24) | 0.39(0.18,0.61) | 9.69(7.58,11.89) | 11.79(8.34,15.47) | 0.71(0.50,0.92) | 1.98(1.55,2.47) | 1.76(1.27,2.27) | -0.26(-0.43,-0.09) | 51.70(39.63,64.80) | 44.41(30.83,58.43) | -0.37(-0.53,-0.21) |
| **Kingdom of Norway** | 1.82(1.68,1.97) | 1.33(1.19,1.48) | -1.17(-1.47,-0.86) | 11.57(10.62,12.61) | 9.17(8.36,10.12) | -0.85(-1.15,-0.55) | 0.69(0.65,0.73) | 0.37(0.33,0.40) | -2.31(-2.57,-2.06) | 17.55(16.51,18.60) | 8.47(7.73,9.24) | -2.56(-2.80,-2.32) |
| **Kingdom of Saudi Arabia** | 1.07(0.79,1.40) | 1.12(0.87,1.45) | -0.04(-0.12,0.05) | 4.96(3.75,6.37) | 6.34(5.03,8.14) | 0.70(0.62,0.78) | 0.90(0.68,1.17) | 0.60(0.47,0.78) | -1.62(-1.76,-1.48) | 23.33(17.24,30.53) | 15.64(12.13,20.73) | -1.54(-1.66,-1.41) |
| **Kingdom of Spain** | 7.95(6.97,8.91) | 4.20(3.61,4.97) | -2.27(-2.43,-2.11) | 44.79(39.29,50.33) | 28.31(24.56,32.92) | -1.69(-1.91,-1.48) | 4.16(3.66,4.65) | 1.49(1.31,1.71) | -3.52(-3.64,-3.40) | 118.91(104.73,134.34) | 38.77(33.58,44.95) | -3.89(-4.04,-3.75) |
| **Kingdom of Sweden** | 1.33(1.20,1.48) | 0.98(0.84,1.14) | -0.63(-0.88,-0.38) | 8.61(7.73,9.58) | 6.73(5.85,7.69) | -0.41(-0.64,-0.18) | 0.48(0.44,0.52) | 0.27(0.23,0.31) | -1.60(-1.85,-1.36) | 11.63(10.59,12.75) | 6.21(5.30,7.20) | -1.65(-1.90,-1.40) |
| **Kingdom of Thailand** | 2.58(2.09,3.07) | 2.29(1.69,3.00) | -0.70(-0.83,-0.57) | 11.71(9.66,13.98) | 12.83(9.73,16.65) | 0.04(-0.06,0.15) | 2.02(1.64,2.38) | 1.23(0.91,1.60) | -1.98(-2.11,-1.84) | 51.35(41.52,61.54) | 33.00(24.19,43.18) | -1.81(-1.95,-1.67) |
| **Kingdom of the Netherlands** | 3.69(3.39,4.04) | 2.14(1.91,2.37) | -1.81(-1.90,-1.71) | 22.81(20.94,24.82) | 14.64(13.31,16.06) | -1.45(-1.57,-1.34) | 1.36(1.27,1.47) | 0.63(0.56,0.68) | -2.71(-2.85,-2.57) | 36.84(34.16,39.80) | 15.49(14.09,17.01) | -2.94(-3.08,-2.80) |
| **Kingdom of Tonga** | 0.65(0.48,0.91) | 0.63(0.44,0.87) | -0.22(-0.43,-0.00) | 2.76(2.04,3.77) | 2.83(2.04,3.87) | -0.04(-0.19,0.11) | 0.57(0.41,0.80) | 0.50(0.36,0.68) | -0.47(-0.70,-0.23) | 12.96(9.46,18.08) | 11.55(8.19,16.16) | -0.43(-0.65,-0.22) |
| **Kyrgyz Republic** | 2.30(1.93,2.72) | 0.95(0.72,1.22) | -2.83(-3.02,-2.65) | 10.36(8.80,12.18) | 4.94(3.86,6.15) | -2.42(-2.58,-2.26) | 1.97(1.66,2.33) | 0.74(0.55,0.93) | -3.16(-3.35,-2.96) | 59.83(49.89,71.41) | 20.78(15.43,26.43) | -3.47(-3.67,-3.27) |
| **Lao People's Democratic Republic** | 1.81(1.28,2.49) | 1.30(0.91,1.90) | -1.12(-1.25,-0.98) | 6.73(4.77,9.25) | 5.45(3.84,7.84) | -0.69(-0.83,-0.56) | 1.76(1.25,2.40) | 1.15(0.80,1.68) | -1.42(-1.52,-1.32) | 46.51(32.32,64.77) | 28.53(19.44,41.98) | -1.63(-1.73,-1.52) |
| **Lebanese Republic** | 3.91(2.72,5.29) | 3.70(2.95,4.65) | 0.26(0.05,0.47) | 18.07(12.57,24.37) | 21.06(16.78,26.42) | 0.89(0.68,1.09) | 2.96(2.10,3.89) | 1.82(1.47,2.26) | -1.23(-1.42,-1.03) | 76.26(51.72,103.82) | 44.20(35.52,56.25) | -1.46(-1.63,-1.29) |
| **Malaysia** | 1.90(1.43,2.33) | 1.89(1.48,2.27) | -0.17(-0.39,0.06) | 8.26(6.37,9.94) | 9.43(7.44,11.13) | 0.35(0.13,0.56) | 1.58(1.20,1.97) | 1.25(0.98,1.50) | -0.99(-1.19,-0.79) | 39.54(30.27,48.36) | 30.68(24.25,36.80) | -1.00(-1.25,-0.75) |
| **Mongolia** | 1.53(1.11,1.98) | 0.94(0.70,1.27) | -1.86(-2.09,-1.63) | 6.35(4.75,8.10) | 4.44(3.35,6.00) | -1.33(-1.48,-1.19) | 1.46(1.07,1.88) | 0.79(0.58,1.06) | -2.34(-2.61,-2.06) | 41.58(29.70,54.62) | 22.36(16.27,30.75) | -2.35(-2.62,-2.08) |
| **Montenegro** | 8.41(6.66,10.63) | 8.71(6.69,11.62) | 0.23(0.05,0.40) | 45.52(36.74,56.77) | 49.48(38.74,64.64) | 0.47(0.32,0.62) | 5.12(4.08,6.43) | 4.67(3.61,6.10) | -0.40(-0.65,-0.16) | 149.64(118.22,188.06) | 127.61(97.86,170.11) | -0.61(-0.89,-0.32) |
| **New Zealand** | 2.67(2.38,2.97) | 1.49(1.28,1.71) | -1.93(-2.20,-1.66) | 16.25(14.55,18.04) | 10.07(8.80,11.43) | -1.61(-1.82,-1.39) | 0.90(0.81,0.98) | 0.37(0.32,0.42) | -2.84(-3.16,-2.51) | 22.51(20.46,24.71) | 8.81(7.83,9.97) | -3.03(-3.29,-2.76) |
| **North Macedonia** | 5.07(4.14,6.32) | 5.00(3.74,6.51) | -0.03(-0.34,0.28) | 25.33(21.19,31.05) | 27.52(21.18,35.35) | 0.41(0.16,0.67) | 4.15(3.39,5.16) | 3.20(2.45,4.13) | -0.97(-1.24,-0.69) | 115.86(95.01,144.76) | 86.15(64.65,113.76) | -1.09(-1.36,-0.81) |
| **Northern Mariana Islands** | 1.52(1.13,1.95) | 0.96(0.78,1.14) | -2.24(-2.65,-1.82) | 7.04(5.25,9.03) | 4.78(3.99,5.66) | -1.99(-2.40,-1.57) | 1.14(0.84,1.45) | 0.66(0.54,0.80) | -2.36(-2.73,-1.99) | 26.37(19.07,34.24) | 15.14(12.33,17.99) | -2.39(-2.73,-2.05) |
| **Palestine** | 1.70(1.26,2.23) | 1.42(1.16,1.76) | -0.87(-1.08,-0.65) | 7.57(5.79,9.70) | 7.43(6.07,9.08) | -0.34(-0.47,-0.22) | 1.43(1.06,1.88) | 0.91(0.75,1.11) | -1.75(-2.03,-1.47) | 33.72(25.04,44.50) | 21.68(17.32,26.91) | -1.68(-1.94,-1.43) |
| **People's Democratic Republic of Algeria** | 2.23(1.71,2.91) | 1.88(1.40,2.53) | -0.51(-0.70,-0.32) | 10.04(7.95,12.81) | 10.15(7.79,13.22) | 0.01(-0.16,0.19) | 1.96(1.51,2.54) | 1.21(0.90,1.59) | -1.46(-1.64,-1.28) | 46.02(35.36,60.91) | 28.50(21.05,37.79) | -1.56(-1.70,-1.41) |
| **People's Republic of Bangladesh** | 4.03(3.14,5.08) | 2.63(1.88,3.56) | -1.45(-1.57,-1.33) | 16.06(12.68,19.96) | 12.25(8.99,16.36) | -0.93(-1.02,-0.85) | 3.86(3.00,4.82) | 2.17(1.57,2.94) | -1.95(-2.10,-1.80) | 105.03(81.68,131.70) | 56.28(39.91,77.04) | -2.05(-2.15,-1.94) |
| **People's Republic of China** | 1.82(1.50,2.13) | 1.79(1.40,2.26) | 0.04(-0.11,0.20) | 7.83(6.47,9.13) | 9.86(7.81,12.35) | 0.90(0.73,1.07) | 1.59(1.32,1.86) | 0.94(0.74,1.17) | -1.74(-1.83,-1.65) | 40.37(33.13,47.60) | 22.73(17.67,28.65) | -1.91(-2.01,-1.81) |
| **Plurinational State of Bolivia** | 1.62(1.17,2.12) | 1.17(0.82,1.65) | -1.04(-1.16,-0.92) | 5.95(4.35,7.75) | 4.73(3.33,6.64) | -0.75(-0.86,-0.63) | 1.62(1.16,2.11) | 1.07(0.75,1.50) | -1.31(-1.41,-1.20) | 39.56(28.43,51.83) | 24.76(16.99,35.11) | -1.54(-1.66,-1.42) |
| **Portuguese Republic** | 4.11(3.74,4.53) | 2.44(2.18,2.76) | -1.68(-1.88,-1.47) | 18.95(17.32,20.77) | 14.26(12.87,15.89) | -0.87(-1.11,-0.63) | 3.46(3.15,3.82) | 1.57(1.39,1.78) | -2.62(-2.78,-2.47) | 95.91(87.33,105.78) | 42.42(37.83,47.86) | -2.65(-2.90,-2.41) |
| **Principality of Andorra** | 3.37(2.26,5.24) | 2.16(1.29,3.19) | -1.29(-1.55,-1.03) | 21.04(14.69,31.57) | 15.26(9.97,21.58) | -0.90(-1.15,-0.65) | 1.36(0.93,2.05) | 0.65(0.41,0.97) | -2.23(-2.48,-1.99) | 37.57(25.58,58.16) | 17.70(10.73,26.84) | -2.25(-2.46,-2.04) |
| **Principality of Monaco** | 14.17(10.15,19.05) | 10.44(7.80,14.22) | -1.03(-1.13,-0.93) | 84.77(61.56,112.94) | 69.02(52.52,93.39) | -0.72(-0.80,-0.64) | 5.44(3.90,7.29) | 3.28(2.50,4.39) | -1.71(-1.81,-1.61) | 151.86(107.39,205.16) | 90.59(68.72,125.08) | -1.74(-1.83,-1.65) |
| **Puerto Rico** | 3.15(2.81,3.49) | 1.62(1.28,2.00) | -1.88(-2.21,-1.55) | 14.74(13.09,16.37) | 9.23(7.35,11.37) | -1.23(-1.57,-0.88) | 2.31(2.06,2.55) | 0.85(0.68,1.04) | -3.03(-3.33,-2.73) | 56.80(50.39,63.16) | 22.13(17.46,27.39) | -2.87(-3.17,-2.56) |
| **Republic of Albania** | 3.69(2.96,4.53) | 3.24(2.29,4.54) | 0.01(-0.18,0.20) | 17.23(14.24,20.81) | 17.62(13.01,24.32) | 0.55(0.38,0.71) | 3.22(2.57,3.95) | 2.05(1.44,2.93) | -1.18(-1.40,-0.96) | 84.41(67.52,104.11) | 54.10(38.65,77.26) | -1.14(-1.36,-0.93) |
| **Republic of Angola** | 1.96(1.36,2.70) | 1.62(1.24,2.09) | -0.59(-0.74,-0.43) | 7.16(5.02,9.75) | 6.49(5.05,8.28) | -0.28(-0.44,-0.12) | 1.93(1.35,2.67) | 1.51(1.16,1.97) | -0.75(-0.88,-0.63) | 51.67(34.96,72.56) | 39.35(29.69,51.05) | -0.85(-0.98,-0.71) |
| **Republic of Armenia** | 4.98(4.74,5.20) | 2.44(2.16,2.74) | -2.30(-2.57,-2.03) | 22.83(21.74,23.83) | 12.98(11.61,14.48) | -1.83(-2.07,-1.59) | 4.04(3.85,4.21) | 1.70(1.52,1.91) | -2.83(-3.10,-2.55) | 116.23(110.20,121.07) | 46.08(41.17,51.83) | -3.06(-3.33,-2.79) |
| **Republic of Austria** | 3.35(3.03,3.70) | 1.93(1.71,2.15) | -1.73(-1.88,-1.57) | 18.67(16.88,20.59) | 12.57(11.20,13.90) | -1.26(-1.41,-1.10) | 1.87(1.72,2.04) | 0.81(0.71,0.89) | -2.63(-2.84,-2.42) | 53.67(48.59,59.45) | 20.69(18.32,23.00) | -3.03(-3.19,-2.86) |
| **Republic of Azerbaijan** | 3.40(2.94,3.96) | 2.25(1.66,3.16) | -1.37(-1.51,-1.23) | 15.05(13.09,17.43) | 10.85(8.24,14.94) | -1.11(-1.23,-0.98) | 2.96(2.55,3.44) | 1.76(1.30,2.46) | -1.72(-1.86,-1.58) | 87.95(76.03,101.65) | 48.69(35.87,69.88) | -2.05(-2.19,-1.91) |
| **Republic of Belarus** | 4.77(4.18,5.45) | 4.11(3.10,5.30) | -1.63(-2.02,-1.24) | 23.34(20.49,26.60) | 22.80(17.45,29.25) | -1.09(-1.44,-0.74) | 3.45(3.03,3.95) | 2.17(1.64,2.76) | -2.69(-3.12,-2.27) | 107.93(94.32,123.91) | 66.38(49.64,84.75) | -2.85(-3.29,-2.41) |
| **Republic of Benin** | 0.98(0.78,1.21) | 1.07(0.79,1.38) | 0.53(0.41,0.65) | 3.83(3.06,4.67) | 4.31(3.18,5.58) | 0.60(0.48,0.73) | 0.96(0.77,1.17) | 1.00(0.75,1.30) | 0.42(0.30,0.54) | 24.62(19.40,30.36) | 25.22(18.30,33.52) | 0.33(0.22,0.44) |
| **Republic of Botswana** | 2.71(1.98,3.41) | 1.94(1.38,2.93) | -1.46(-1.76,-1.16) | 10.85(8.12,13.60) | 8.11(5.87,12.14) | -1.14(-1.28,-1.00) | 2.50(1.85,3.14) | 1.69(1.21,2.54) | -1.63(-1.97,-1.29) | 69.18(50.38,89.45) | 45.85(32.14,72.01) | -1.76(-2.14,-1.38) |
| **Republic of Bulgaria** | 4.41(3.77,5.16) | 5.63(4.47,7.02) | 1.01(0.78,1.25) | 22.71(19.48,26.34) | 31.01(25.08,38.32) | 1.22(1.00,1.44) | 3.07(2.61,3.54) | 3.13(2.49,3.85) | 0.33(0.09,0.57) | 91.61(78.35,106.50) | 89.58(71.08,110.54) | 0.08(-0.19,0.34) |
| **Republic of Burundi** | 2.31(1.48,3.19) | 1.47(1.03,2.06) | -1.84(-2.09,-1.59) | 8.50(5.57,11.68) | 5.95(4.29,8.24) | -1.38(-1.59,-1.18) | 2.25(1.45,3.11) | 1.40(0.98,1.99) | -1.89(-2.13,-1.65) | 63.16(40.41,88.82) | 37.83(26.29,54.50) | -2.05(-2.30,-1.79) |
| **Republic of Cabo Verde** | 1.66(1.29,2.08) | 1.79(1.23,2.30) | 0.02(-0.51,0.55) | 6.97(5.51,8.62) | 7.90(5.50,10.23) | 0.28(-0.20,0.77) | 1.54(1.21,1.91) | 1.43(0.98,1.80) | -0.52(-1.04,-0.00) | 36.70(29.06,46.34) | 34.19(23.56,44.44) | -0.47(-0.95,0.01) |
| **Republic of Cameroon** | 1.07(0.84,1.38) | 1.34(0.89,1.89) | 0.97(0.83,1.11) | 4.26(3.38,5.43) | 5.45(3.70,7.62) | 1.01(0.90,1.13) | 1.03(0.81,1.32) | 1.23(0.82,1.75) | 0.85(0.69,1.02) | 26.71(20.83,34.71) | 31.66(20.77,45.67) | 0.79(0.61,0.96) |
| **Republic of Chad** | 0.81(0.62,1.05) | 1.36(0.95,1.81) | 1.98(1.81,2.16) | 3.19(2.45,3.99) | 5.26(3.76,6.93) | 1.92(1.79,2.05) | 0.80(0.62,1.03) | 1.34(0.95,1.78) | 1.96(1.77,2.14) | 20.60(15.83,26.34) | 33.63(23.50,45.20) | 1.87(1.67,2.07) |
| **Republic of Chile** | 1.95(1.76,2.16) | 1.14(1.02,1.27) | -1.58(-1.71,-1.45) | 9.98(9.12,11.03) | 7.03(6.27,7.77) | -1.00(-1.11,-0.89) | 1.47(1.33,1.63) | 0.59(0.52,0.66) | -2.79(-2.92,-2.66) | 38.85(35.22,42.96) | 14.95(13.45,16.66) | -2.94(-3.06,-2.83) |
| **Republic of Colombia** | 2.79(2.57,3.01) | 1.23(0.99,1.50) | -3.31(-3.52,-3.10) | 11.55(10.63,12.46) | 6.01(4.86,7.37) | -2.73(-2.94,-2.52) | 2.48(2.29,2.67) | 0.88(0.71,1.06) | -3.99(-4.19,-3.79) | 59.91(54.87,64.33) | 19.59(15.76,24.24) | -4.22(-4.41,-4.03) |
| **Republic of Costa Rica** | 2.27(2.05,2.50) | 1.09(0.94,1.28) | -2.45(-2.66,-2.25) | 10.42(9.47,11.47) | 5.55(4.79,6.52) | -2.16(-2.36,-1.96) | 1.74(1.57,1.90) | 0.72(0.62,0.84) | -2.88(-3.10,-2.66) | 41.55(37.42,45.67) | 16.59(14.26,19.34) | -3.13(-3.35,-2.92) |
| **Republic of Côte d'Ivoire** | 1.34(1.07,1.69) | 1.23(0.86,1.82) | -0.53(-0.69,-0.37) | 5.26(4.22,6.60) | 5.20(3.73,7.72) | -0.22(-0.32,-0.12) | 1.28(1.01,1.62) | 1.11(0.80,1.66) | -0.69(-0.88,-0.50) | 34.34(26.95,44.03) | 29.74(20.60,45.91) | -0.73(-0.93,-0.53) |
| **Republic of Croatia** | 6.61(5.98,7.32) | 4.09(3.38,4.82) | -1.55(-1.72,-1.37) | 33.14(30.01,36.53) | 24.41(20.54,28.42) | -1.00(-1.19,-0.81) | 4.49(4.06,4.95) | 2.06(1.74,2.41) | -2.53(-2.68,-2.38) | 127.95(115.47,141.20) | 55.67(46.73,64.51) | -2.76(-2.94,-2.57) |
| **Republic of Cuba** | 5.77(5.20,6.42) | 7.80(6.47,9.43) | 1.10(0.96,1.25) | 27.20(24.75,30.05) | 40.96(34.04,49.37) | 1.49(1.33,1.65) | 4.24(3.83,4.71) | 4.65(3.93,5.61) | 0.40(0.26,0.54) | 104.64(94.65,116.01) | 117.58(97.77,142.57) | 0.56(0.41,0.71) |
| **Republic of Cyprus** | 2.47(1.94,3.05) | 2.14(1.67,2.74) | 0.02(-0.18,0.21) | 13.37(11.00,16.23) | 14.49(11.69,18.02) | 0.78(0.56,1.00) | 1.59(1.26,1.97) | 0.74(0.59,0.94) | -2.40(-2.46,-2.33) | 35.64(28.62,44.07) | 18.70(14.69,24.18) | -1.91(-1.98,-1.83) |
| **Republic of Djibouti** | 2.10(1.45,3.09) | 1.88(1.28,2.63) | -0.50(-0.58,-0.41) | 8.43(5.93,12.32) | 7.94(5.50,11.07) | -0.31(-0.37,-0.25) | 1.98(1.39,2.92) | 1.70(1.17,2.35) | -0.62(-0.73,-0.50) | 54.56(37.66,82.46) | 45.66(30.90,64.09) | -0.72(-0.85,-0.58) |
| **Republic of Ecuador** | 1.11(1.03,1.21) | 0.68(0.52,0.87) | -1.62(-1.90,-1.35) | 4.48(4.14,4.86) | 3.12(2.40,4.00) | -1.30(-1.57,-1.03) | 1.06(0.97,1.15) | 0.54(0.43,0.69) | -2.08(-2.35,-1.80) | 24.94(23.05,27.08) | 12.55(9.62,16.23) | -2.25(-2.53,-1.98) |
| **Republic of El Salvador** | 1.03(0.88,1.20) | 0.90(0.70,1.16) | -0.78(-0.99,-0.57) | 4.16(3.59,4.83) | 4.23(3.27,5.45) | -0.28(-0.48,-0.07) | 0.96(0.82,1.13) | 0.67(0.52,0.86) | -1.52(-1.73,-1.31) | 23.58(20.22,27.49) | 16.59(12.76,21.40) | -1.50(-1.73,-1.27) |
| **Republic of Equatorial Guinea** | 1.95(1.22,2.77) | 1.43(0.86,2.10) | -1.10(-1.48,-0.71) | 7.01(4.37,9.95) | 6.12(3.77,8.89) | -0.48(-0.81,-0.16) | 1.92(1.23,2.72) | 1.22(0.73,1.78) | -1.59(-1.94,-1.23) | 52.08(31.45,75.23) | 31.17(18.43,45.99) | -1.85(-2.24,-1.45) |
| **Republic of Estonia** | 4.36(3.84,4.95) | 3.32(2.69,4.15) | -1.30(-1.60,-0.99) | 22.98(20.14,26.04) | 20.73(17.04,25.64) | -0.66(-0.92,-0.39) | 2.50(2.23,2.81) | 1.18(0.96,1.43) | -3.07(-3.40,-2.73) | 75.35(66.71,85.12) | 33.37(26.91,41.01) | -3.36(-3.70,-3.02) |
| **Republic of Fiji** | 0.81(0.64,1.03) | 0.79(0.57,1.04) | -0.01(-0.15,0.12) | 3.52(2.85,4.35) | 3.41(2.59,4.42) | -0.02(-0.16,0.12) | 0.72(0.56,0.93) | 0.68(0.50,0.89) | -0.09(-0.23,0.04) | 17.71(13.78,22.68) | 16.14(11.70,21.12) | -0.18(-0.32,-0.03) |
| **Republic of Finland** | 2.09(1.91,2.31) | 1.59(1.41,1.79) | -0.88(-1.08,-0.68) | 12.87(11.74,14.09) | 10.97(9.84,12.28) | -0.49(-0.66,-0.33) | 0.80(0.73,0.88) | 0.40(0.36,0.45) | -2.25(-2.54,-1.97) | 22.72(20.70,25.13) | 11.05(9.93,12.30) | -2.37(-2.62,-2.12) |
| **Republic of Ghana** | 0.84(0.66,1.10) | 1.20(0.85,1.55) | 1.77(1.52,2.01) | 3.43(2.71,4.40) | 4.93(3.59,6.31) | 1.70(1.50,1.90) | 0.80(0.64,1.05) | 1.09(0.78,1.41) | 1.63(1.36,1.89) | 20.79(16.02,27.37) | 27.53(19.56,36.04) | 1.55(1.28,1.81) |
| **Republic of Guatemala** | 1.65(1.57,1.74) | 0.56(0.48,0.65) | -3.70(-3.92,-3.49) | 5.97(5.69,6.26) | 2.50(2.15,2.93) | -2.98(-3.19,-2.78) | 1.68(1.59,1.76) | 0.50(0.42,0.58) | -4.10(-4.32,-3.89) | 38.17(36.42,40.23) | 11.61(9.87,13.61) | -4.02(-4.24,-3.80) |
| **Republic of Guinea** | 0.84(0.64,1.09) | 1.28(0.94,1.65) | 1.87(1.68,2.05) | 3.35(2.58,4.25) | 5.03(3.77,6.46) | 1.77(1.61,1.93) | 0.84(0.64,1.07) | 1.23(0.91,1.58) | 1.80(1.60,1.99) | 21.38(16.13,27.58) | 31.12(22.54,40.88) | 1.74(1.54,1.94) |
| **Republic of Guinea-Bissau** | 1.42(0.92,1.97) | 1.58(1.08,2.13) | 0.79(0.63,0.96) | 5.14(3.32,7.08) | 6.04(4.19,8.20) | 0.92(0.77,1.07) | 1.40(0.91,1.94) | 1.52(1.04,2.02) | 0.71(0.55,0.87) | 37.32(23.55,52.98) | 40.06(26.73,54.84) | 0.66(0.51,0.81) |
| **Republic of Guyana** | 1.27(1.10,1.48) | 1.22(0.91,1.61) | 0.41(0.18,0.64) | 4.86(4.23,5.67) | 5.13(3.87,6.73) | 0.63(0.41,0.85) | 1.20(1.04,1.40) | 1.07(0.80,1.40) | 0.22(-0.01,0.46) | 31.53(27.06,36.80) | 29.22(21.71,38.59) | 0.34(0.10,0.58) |
| **Republic of Haiti** | 2.83(1.95,3.72) | 2.34(1.50,3.28) | -0.44(-0.53,-0.36) | 10.24(7.00,13.46) | 8.89(5.82,12.34) | -0.33(-0.49,-0.18) | 2.83(1.96,3.71) | 2.31(1.49,3.24) | -0.49(-0.58,-0.41) | 74.01(50.03,97.86) | 57.84(37.34,81.36) | -0.63(-0.74,-0.53) |
| **Republic of Honduras** | 1.21(0.98,1.51) | 1.58(1.18,2.04) | 1.09(0.96,1.22) | 4.82(3.95,6.00) | 6.32(4.81,8.13) | 1.05(0.95,1.15) | 1.18(0.97,1.46) | 1.45(1.07,1.87) | 0.94(0.79,1.08) | 28.82(23.64,35.87) | 33.52(25.27,43.43) | 0.70(0.58,0.82) |
| **Republic of Iceland** | 2.04(1.82,2.29) | 1.27(1.11,1.46) | -1.73(-1.89,-1.57) | 12.90(11.54,14.49) | 8.89(7.76,10.13) | -1.41(-1.55,-1.27) | 0.81(0.73,0.89) | 0.36(0.31,0.41) | -2.72(-2.90,-2.54) | 21.52(19.34,23.88) | 9.63(8.38,10.97) | -2.78(-2.98,-2.57) |
| **Republic of India** | 3.15(2.56,3.77) | 2.84(2.48,3.29) | -0.47(-0.66,-0.28) | 12.95(10.93,15.35) | 12.92(11.32,14.80) | -0.16(-0.36,0.04) | 2.95(2.38,3.53) | 2.35(2.05,2.72) | -0.84(-0.99,-0.69) | 82.09(66.95,98.23) | 62.95(54.37,72.82) | -0.98(-1.12,-0.84) |
| **Republic of Indonesia** | 1.19(0.88,1.44) | 1.26(0.89,1.66) | 0.18(0.15,0.22) | 5.08(3.93,6.09) | 5.66(4.13,7.36) | 0.32(0.27,0.37) | 1.08(0.79,1.32) | 1.03(0.72,1.34) | -0.15(-0.20,-0.11) | 28.01(21.10,34.11) | 25.21(18.01,33.31) | -0.33(-0.37,-0.29) |
| **Republic of Iraq** | 3.02(2.32,3.84) | 3.23(2.28,4.28) | -0.06(-0.19,0.07) | 13.66(10.73,17.19) | 16.35(11.75,21.41) | 0.38(0.25,0.52) | 2.46(1.88,3.13) | 2.02(1.45,2.67) | -0.99(-1.10,-0.88) | 66.88(51.49,85.60) | 50.56(35.99,67.05) | -1.25(-1.35,-1.14) |
| **Republic of Italy** | 5.97(5.59,6.34) | 3.02(2.77,3.29) | -2.21(-2.31,-2.10) | 34.32(32.11,36.49) | 20.14(18.72,21.72) | -1.78(-1.92,-1.64) | 2.93(2.79,3.06) | 1.11(1.02,1.19) | -3.13(-3.25,-3.01) | 78.40(75.14,82.00) | 26.96(25.19,29.05) | -3.42(-3.54,-3.30) |
| **Republic of Kazakhstan** | 3.86(3.53,4.17) | 1.63(1.41,1.88) | -3.32(-3.54,-3.10) | 17.26(15.99,18.62) | 8.52(7.43,9.62) | -2.75(-2.91,-2.58) | 3.19(2.95,3.45) | 1.15(1.00,1.32) | -3.89(-4.21,-3.57) | 96.40(88.91,103.84) | 33.24(28.57,38.05) | -4.14(-4.47,-3.81) |
| **Republic of Kenya** | 1.24(0.90,1.69) | 1.45(1.07,1.86) | 0.54(0.36,0.73) | 5.29(3.94,7.03) | 6.19(4.70,7.91) | 0.58(0.47,0.70) | 1.15(0.84,1.57) | 1.29(0.95,1.64) | 0.46(0.25,0.67) | 30.94(22.44,42.19) | 34.89(25.69,45.22) | 0.50(0.24,0.75) |
| **Republic of Kiribati** | 0.21(0.16,0.27) | 0.23(0.16,0.32) | 0.17(0.06,0.27) | 0.82(0.65,1.07) | 0.89(0.66,1.26) | 0.23(0.15,0.31) | 0.21(0.16,0.27) | 0.22(0.16,0.30) | 0.09(-0.01,0.19) | 5.33(4.16,6.98) | 5.47(3.91,7.84) | 0.06(-0.05,0.17) |
| **Republic of Korea** | 4.04(2.84,5.14) | 2.15(1.59,2.80) | -2.74(-3.03,-2.45) | 21.32(15.28,26.84) | 14.62(11.15,18.56) | -1.85(-2.17,-1.54) | 2.30(1.63,2.91) | 0.53(0.39,0.68) | -5.65(-6.01,-5.28) | 57.12(40.47,72.48) | 11.70(8.70,15.01) | -5.95(-6.29,-5.60) |
| **Republic of Latvia** | 4.18(3.74,4.70) | 2.89(2.37,3.43) | -1.51(-1.74,-1.28) | 19.99(17.78,22.44) | 15.71(13.12,18.60) | -1.08(-1.28,-0.88) | 3.07(2.77,3.45) | 1.72(1.42,2.07) | -2.11(-2.31,-1.91) | 92.71(82.68,105.17) | 49.73(40.99,60.21) | -2.37(-2.60,-2.15) |
| **Republic of Liberia** | 0.93(0.67,1.36) | 1.12(0.77,1.57) | 0.80(0.64,0.97) | 3.61(2.67,5.14) | 4.63(3.25,6.49) | 1.00(0.82,1.17) | 0.92(0.67,1.33) | 1.03(0.72,1.45) | 0.57(0.41,0.73) | 23.28(16.66,34.10) | 25.86(17.33,36.85) | 0.53(0.36,0.69) |
| **Republic of Lithuania** | 4.61(4.02,5.31) | 3.44(2.73,4.25) | -1.21(-1.45,-0.96) | 22.30(19.54,25.57) | 18.55(14.83,22.62) | -0.89(-1.09,-0.68) | 3.38(2.95,3.87) | 2.11(1.67,2.60) | -1.67(-1.92,-1.43) | 102.48(89.45,117.70) | 60.78(47.67,74.35) | -1.94(-2.21,-1.67) |
| **Republic of Madagascar** | 1.66(1.23,2.09) | 1.13(0.79,1.52) | -1.31(-1.45,-1.17) | 6.62(5.01,8.21) | 4.75(3.49,6.30) | -1.13(-1.27,-0.99) | 1.59(1.18,2.00) | 1.05(0.75,1.40) | -1.39(-1.52,-1.25) | 43.90(32.30,55.00) | 28.66(20.24,38.43) | -1.42(-1.55,-1.29) |
| **Republic of Malawi** | 0.60(0.46,0.79) | 0.62(0.47,0.86) | -0.11(-0.30,0.08) | 2.35(1.86,3.09) | 2.59(1.98,3.56) | 0.18(0.07,0.28) | 0.58(0.44,0.77) | 0.57(0.43,0.78) | -0.28(-0.48,-0.08) | 15.80(12.26,21.13) | 15.87(11.85,22.32) | -0.24(-0.46,-0.02) |
| **Republic of Maldives** | 1.37(0.99,1.72) | 0.77(0.57,0.99) | -2.12(-2.25,-1.99) | 5.57(4.13,7.00) | 3.95(3.00,5.08) | -1.31(-1.44,-1.18) | 1.26(0.92,1.58) | 0.49(0.37,0.63) | -3.38(-3.53,-3.23) | 29.57(21.07,38.03) | 10.92(8.04,14.24) | -3.60(-3.79,-3.41) |
| **Republic of Mali** | 1.09(0.92,1.31) | 1.03(0.77,1.39) | 0.08(-0.08,0.24) | 4.29(3.60,5.14) | 4.25(3.22,5.72) | 0.18(0.04,0.33) | 1.06(0.90,1.27) | 0.96(0.72,1.31) | -0.02(-0.17,0.14) | 28.28(23.49,34.40) | 25.15(18.49,34.83) | -0.10(-0.26,0.06) |
| **Republic of Malta** | 3.86(3.35,4.40) | 2.31(1.93,2.76) | -1.71(-1.80,-1.63) | 21.89(19.03,24.96) | 15.53(13.14,18.43) | -1.16(-1.25,-1.07) | 1.98(1.73,2.24) | 0.78(0.66,0.90) | -3.12(-3.21,-3.03) | 51.64(44.80,58.73) | 21.42(18.01,25.46) | -2.88(-2.96,-2.79) |
| **Republic of Mauritius** | 2.73(2.55,2.96) | 2.31(2.11,2.49) | -0.34(-1.10,0.42) | 12.25(11.45,13.28) | 11.85(10.82,12.76) | 0.03(-0.64,0.72) | 2.04(1.92,2.21) | 1.46(1.33,1.58) | -0.83(-1.65,0.00) | 53.80(50.40,58.18) | 37.82(34.29,40.60) | -0.81(-1.63,0.01) |
| **Republic of Moldova** | 4.33(4.07,4.60) | 3.82(3.35,4.35) | -0.47(-0.82,-0.12) | 19.92(18.72,21.11) | 20.07(17.86,22.73) | -0.06(-0.38,0.27) | 3.48(3.27,3.68) | 2.44(2.16,2.77) | -1.19(-1.48,-0.89) | 104.34(98.44,110.65) | 72.89(64.66,82.56) | -1.22(-1.51,-0.93) |
| **Republic of Mozambique** | 1.90(1.39,2.52) | 1.92(1.40,2.48) | 0.39(0.25,0.52) | 7.33(5.43,9.61) | 7.54(5.60,9.68) | 0.38(0.28,0.49) | 1.86(1.38,2.44) | 1.80(1.32,2.32) | 0.28(0.13,0.42) | 50.31(36.68,67.47) | 50.27(36.61,66.06) | 0.41(0.25,0.57) |
| **Republic of Namibia** | 2.68(2.18,3.29) | 2.87(2.09,3.83) | 0.05(-0.21,0.32) | 10.97(9.02,13.35) | 12.53(9.19,16.81) | 0.31(0.14,0.48) | 2.48(2.01,3.03) | 2.42(1.80,3.20) | -0.24(-0.53,0.06) | 68.66(55.01,85.29) | 67.74(48.71,91.69) | -0.23(-0.55,0.10) |
| **Republic of Nauru** | 1.15(0.77,1.54) | 0.87(0.59,1.20) | -1.06(-1.24,-0.87) | 4.37(2.95,5.78) | 3.60(2.43,4.93) | -0.77(-0.88,-0.65) | 1.09(0.74,1.44) | 0.76(0.52,1.04) | -1.28(-1.51,-1.04) | 25.67(16.59,34.53) | 18.90(12.27,26.60) | -1.13(-1.40,-0.87) |
| **Republic of Nicaragua** | 1.33(1.07,1.61) | 0.96(0.74,1.25) | -1.09(-1.25,-0.93) | 5.49(4.45,6.65) | 4.48(3.49,5.78) | -0.68(-0.82,-0.55) | 1.23(0.99,1.49) | 0.75(0.59,0.98) | -1.64(-1.83,-1.45) | 28.99(23.49,35.29) | 18.00(13.88,23.52) | -1.61(-1.77,-1.45) |
| **Republic of Niue** | 0.63(0.46,0.83) | 0.65(0.49,0.86) | -0.16(-0.32,-0.00) | 2.73(2.01,3.55) | 2.95(2.24,3.86) | 0.02(-0.10,0.15) | 0.53(0.39,0.69) | 0.49(0.37,0.64) | -0.48(-0.65,-0.30) | 13.17(9.46,17.31) | 11.62(8.56,15.53) | -0.67(-0.83,-0.51) |
| **Republic of Palau** | 0.69(0.51,0.91) | 0.60(0.43,0.78) | -0.62(-0.74,-0.50) | 2.92(2.16,3.80) | 2.77(2.04,3.56) | -0.30(-0.42,-0.17) | 0.58(0.43,0.76) | 0.45(0.33,0.61) | -0.90(-1.01,-0.79) | 14.10(10.07,18.67) | 11.08(7.94,14.63) | -0.87(-0.98,-0.76) |
| **Republic of Panama** | 1.88(1.74,2.00) | 1.00(0.78,1.22) | -2.36(-2.56,-2.16) | 8.01(7.44,8.51) | 4.88(3.80,5.93) | -1.97(-2.17,-1.77) | 1.63(1.51,1.73) | 0.72(0.57,0.88) | -2.84(-3.04,-2.63) | 36.83(34.30,39.08) | 16.36(12.62,20.09) | -2.86(-3.08,-2.64) |
| **Republic of Paraguay** | 1.54(1.19,1.96) | 2.18(1.52,3.00) | 1.27(1.18,1.35) | 6.84(5.33,8.75) | 10.36(7.24,14.19) | 1.43(1.35,1.51) | 1.31(1.02,1.68) | 1.64(1.14,2.24) | 0.91(0.81,1.00) | 34.83(26.88,44.97) | 43.21(29.54,59.86) | 0.82(0.72,0.92) |
| **Republic of Peru** | 1.23(0.99,1.50) | 0.78(0.55,1.09) | -1.91(-2.33,-1.48) | 4.89(3.95,5.93) | 3.76(2.71,5.24) | -1.25(-1.67,-0.83) | 1.17(0.94,1.42) | 0.57(0.40,0.78) | -2.77(-3.15,-2.39) | 27.54(22.11,33.40) | 12.89(9.11,17.92) | -2.92(-3.32,-2.51) |
| **Republic of Poland** | 5.18(4.98,5.41) | 3.92(3.51,4.32) | -1.13(-1.34,-0.92) | 24.07(23.02,25.22) | 21.17(19.10,23.17) | -0.62(-0.86,-0.37) | 4.20(4.05,4.37) | 2.50(2.25,2.75) | -1.90(-2.06,-1.73) | 126.53(121.93,131.06) | 67.32(60.35,74.18) | -2.25(-2.43,-2.07) |
| **Republic of Rwanda** | 2.69(1.92,3.49) | 1.58(1.09,2.29) | -2.61(-2.96,-2.25) | 9.83(7.19,12.60) | 6.68(4.65,9.53) | -1.81(-2.16,-1.47) | 2.62(1.88,3.41) | 1.45(0.99,2.11) | -2.81(-3.16,-2.47) | 73.47(52.15,94.96) | 38.65(26.17,57.17) | -3.04(-3.41,-2.66) |
| **Republic of San Marino** | 5.86(4.47,7.39) | 2.64(1.54,3.99) | -1.85(-2.19,-1.50) | 36.05(28.06,45.19) | 18.79(12.08,27.12) | -1.51(-1.83,-1.18) | 2.20(1.71,2.74) | 0.82(0.50,1.21) | -2.35(-2.68,-2.01) | 58.64(44.79,73.71) | 21.94(12.84,32.87) | -2.43(-2.73,-2.13) |
| **Republic of Senegal** | 1.05(0.83,1.28) | 1.23(0.90,1.60) | 0.85(0.65,1.05) | 4.17(3.35,5.01) | 5.05(3.73,6.54) | 0.90(0.71,1.09) | 1.01(0.80,1.23) | 1.15(0.83,1.49) | 0.78(0.57,0.98) | 26.27(20.73,32.01) | 28.56(20.82,37.65) | 0.61(0.41,0.80) |
| **Republic of Serbia** | 5.59(3.98,7.85) | 5.55(3.82,7.62) | -0.10(-0.33,0.13) | 27.78(20.55,38.53) | 32.47(22.97,43.81) | 0.49(0.27,0.71) | 4.13(2.98,5.76) | 2.79(1.93,3.86) | -1.43(-1.64,-1.23) | 114.44(81.84,162.55) | 77.06(52.51,107.73) | -1.52(-1.77,-1.27) |
| **Republic of Seychelles** | 6.49(5.50,7.68) | 5.87(4.78,7.08) | 0.34(0.04,0.63) | 28.09(23.86,33.07) | 29.01(23.82,34.94) | 0.76(0.50,1.01) | 5.19(4.43,6.12) | 3.94(3.22,4.73) | -0.23(-0.54,0.07) | 137.92(116.90,162.74) | 100.72(81.77,121.39) | -0.36(-0.64,-0.08) |
| **Republic of Sierra Leone** | 1.01(0.76,1.30) | 1.15(0.83,1.58) | 0.88(0.68,1.08) | 3.91(3.00,5.06) | 4.65(3.36,6.44) | 0.96(0.76,1.16) | 0.98(0.74,1.27) | 1.08(0.78,1.50) | 0.78(0.59,0.98) | 25.34(18.90,33.35) | 27.58(19.45,39.26) | 0.75(0.56,0.95) |
| **Republic of Singapore** | 3.42(3.05,3.78) | 1.62(1.40,1.86) | -2.86(-3.22,-2.49) | 19.68(17.69,21.90) | 11.26(9.84,12.87) | -2.19(-2.54,-1.84) | 1.55(1.40,1.70) | 0.41(0.36,0.47) | -4.90(-5.32,-4.48) | 38.10(34.30,41.90) | 9.07(7.91,10.24) | -5.15(-5.49,-4.80) |
| **Republic of Slovenia** | 4.32(3.89,4.85) | 3.14(2.60,3.77) | -1.03(-1.22,-0.84) | 23.03(20.89,25.62) | 20.05(16.86,23.74) | -0.39(-0.56,-0.21) | 2.67(2.42,2.99) | 1.21(1.01,1.42) | -2.78(-2.99,-2.57) | 78.25(70.59,87.14) | 33.10(27.51,39.67) | -3.01(-3.23,-2.79) |
| **Republic of South Africa** | 2.19(1.77,3.00) | 1.96(1.72,2.19) | -0.66(-0.93,-0.39) | 10.05(8.24,13.40) | 8.97(7.88,10.04) | -0.63(-0.79,-0.46) | 1.87(1.52,2.58) | 1.56(1.38,1.76) | -0.89(-1.22,-0.55) | 54.53(44.31,73.90) | 43.71(38.32,49.53) | -1.03(-1.36,-0.70) |
| **Republic of South Sudan** | 2.07(1.44,2.78) | 1.64(1.07,2.37) | -0.99(-1.19,-0.80) | 8.00(5.68,10.68) | 6.64(4.46,9.44) | -0.83(-1.01,-0.65) | 1.98(1.40,2.66) | 1.53(1.00,2.20) | -1.06(-1.23,-0.89) | 54.02(37.23,73.37) | 41.26(26.62,60.72) | -1.12(-1.32,-0.91) |
| **Republic of Sudan** | 2.58(1.57,4.12) | 2.31(1.45,3.44) | -0.48(-0.55,-0.40) | 10.43(6.69,15.94) | 10.64(6.80,15.64) | -0.06(-0.15,0.02) | 2.40(1.48,3.87) | 1.84(1.17,2.71) | -0.95(-0.99,-0.91) | 62.49(38.40,97.36) | 45.94(28.28,68.29) | -1.09(-1.13,-1.05) |
| **Republic of Suriname** | 0.98(0.80,1.15) | 0.93(0.66,1.30) | 0.05(-0.16,0.26) | 4.08(3.40,4.82) | 4.13(2.97,5.65) | 0.24(0.05,0.42) | 0.89(0.73,1.05) | 0.78(0.55,1.09) | -0.20(-0.41,0.01) | 22.95(18.86,27.30) | 20.72(14.70,28.94) | -0.16(-0.36,0.05) |
| **Republic of Tajikistan** | 1.75(1.31,2.22) | 0.98(0.68,1.37) | -2.01(-2.23,-1.79) | 7.86(6.12,9.82) | 4.62(3.36,6.24) | -1.91(-2.07,-1.75) | 1.58(1.18,1.99) | 0.86(0.60,1.20) | -2.05(-2.31,-1.79) | 46.06(34.15,58.65) | 24.04(16.28,33.99) | -2.26(-2.46,-2.06) |
| **Republic of the Congo** | 2.15(1.38,2.81) | 1.71(1.29,2.26) | -0.94(-1.12,-0.75) | 7.90(5.14,10.31) | 6.99(5.29,9.09) | -0.54(-0.71,-0.37) | 2.09(1.37,2.69) | 1.58(1.19,2.07) | -1.11(-1.27,-0.95) | 56.20(35.49,74.20) | 40.66(29.95,54.07) | -1.27(-1.44,-1.10) |
| **Republic of the Gambia** | 0.53(0.40,0.68) | 0.51(0.38,0.66) | -0.24(-0.41,-0.07) | 2.17(1.68,2.80) | 2.14(1.63,2.73) | -0.19(-0.36,-0.01) | 0.49(0.38,0.63) | 0.46(0.34,0.59) | -0.35(-0.50,-0.20) | 13.41(10.17,17.55) | 12.26(9.06,15.91) | -0.44(-0.62,-0.27) |
| **Republic of the Marshall Islands** | 0.83(0.59,1.15) | 0.83(0.55,1.19) | 0.13(0.01,0.25) | 3.13(2.28,4.22) | 3.34(2.29,4.66) | 0.26(0.17,0.35) | 0.79(0.56,1.08) | 0.78(0.51,1.11) | 0.06(-0.08,0.21) | 19.32(13.62,26.70) | 18.20(11.85,26.12) | -0.13(-0.27,0.02) |
| **Republic of the Niger** | 0.88(0.64,1.16) | 0.97(0.66,1.36) | 0.74(0.57,0.90) | 3.40(2.53,4.38) | 3.76(2.61,5.23) | 0.69(0.53,0.85) | 0.87(0.64,1.14) | 0.96(0.66,1.33) | 0.71(0.56,0.87) | 22.57(16.56,29.80) | 23.00(15.52,32.65) | 0.46(0.30,0.62) |
| **Republic of the Philippines** | 1.10(0.92,1.35) | 1.04(0.84,1.25) | -0.25(-0.32,-0.18) | 4.96(4.19,6.04) | 4.84(3.96,5.78) | -0.24(-0.30,-0.17) | 0.95(0.79,1.16) | 0.83(0.68,1.00) | -0.39(-0.48,-0.30) | 24.31(20.30,29.64) | 21.43(17.18,25.84) | -0.48(-0.58,-0.38) |
| **Republic of the Union of Myanmar** | 1.72(1.12,2.35) | 1.07(0.76,1.51) | -1.74(-1.88,-1.59) | 6.60(4.28,8.94) | 4.77(3.41,6.64) | -1.20(-1.36,-1.04) | 1.64(1.08,2.21) | 0.91(0.65,1.28) | -2.11(-2.22,-2.00) | 42.76(27.02,58.98) | 22.31(15.71,31.62) | -2.31(-2.43,-2.20) |
| **Republic of Trinidad and Tobago** | 1.65(1.54,1.77) | 1.52(1.15,1.96) | -0.31(-0.45,-0.17) | 6.87(6.39,7.37) | 7.19(5.43,9.31) | 0.17(0.04,0.30) | 1.43(1.34,1.53) | 1.14(0.87,1.47) | -0.85(-1.00,-0.70) | 36.42(33.81,39.08) | 30.13(22.91,39.14) | -0.74(-0.90,-0.58) |
| **Republic of Tunisia** | 3.49(2.68,4.46) | 3.76(2.63,5.33) | 0.04(-0.02,0.11) | 16.39(12.72,20.88) | 20.80(14.72,29.18) | 0.61(0.55,0.68) | 2.72(2.11,3.42) | 2.02(1.44,2.83) | -1.15(-1.23,-1.08) | 67.34(51.58,86.12) | 51.00(35.44,72.13) | -1.09(-1.16,-1.02) |
| **Republic of Turkey** | 4.34(3.15,5.88) | 3.31(2.54,4.27) | -1.05(-1.25,-0.85) | 18.71(13.97,24.87) | 18.41(14.32,23.57) | -0.18(-0.33,-0.03) | 3.68(2.66,5.00) | 1.77(1.39,2.27) | -2.63(-2.85,-2.42) | 94.95(68.71,127.50) | 43.30(33.49,55.67) | -2.84(-3.04,-2.65) |
| **Republic of Uganda** | 2.33(1.76,2.96) | 1.91(1.35,2.65) | -1.33(-1.58,-1.07) | 8.85(6.70,11.29) | 8.03(5.71,11.09) | -0.90(-1.12,-0.69) | 2.22(1.69,2.82) | 1.74(1.25,2.42) | -1.46(-1.73,-1.19) | 59.89(44.58,76.97) | 46.99(32.35,66.35) | -1.53(-1.83,-1.23) |
| **Republic of Uzbekistan** | 2.46(2.15,2.80) | 0.78(0.62,0.98) | -3.39(-4.05,-2.73) | 11.21(9.87,12.69) | 3.92(3.18,4.79) | -3.13(-3.67,-2.59) | 2.10(1.86,2.38) | 0.65(0.52,0.80) | -3.52(-4.21,-2.83) | 63.01(54.78,72.37) | 17.79(14.03,22.45) | -3.83(-4.49,-3.16) |
| **Republic of Vanuatu** | 0.63(0.43,0.86) | 0.52(0.37,0.71) | -0.79(-0.88,-0.69) | 2.39(1.67,3.27) | 2.06(1.49,2.78) | -0.67(-0.75,-0.58) | 0.60(0.41,0.83) | 0.49(0.35,0.66) | -0.84(-0.95,-0.74) | 14.07(9.25,19.95) | 11.48(8.06,15.90) | -0.85(-0.95,-0.74) |
| **Republic of Yemen** | 2.92(1.83,4.13) | 2.83(1.75,4.10) | -0.14(-0.19,-0.09) | 11.93(7.74,16.86) | 12.45(8.02,17.61) | 0.11(0.06,0.16) | 2.67(1.67,3.80) | 2.39(1.49,3.44) | -0.43(-0.49,-0.38) | 72.21(44.78,102.70) | 60.21(37.12,87.82) | -0.69(-0.76,-0.63) |
| **Republic of Zambia** | 2.17(1.61,2.74) | 2.26(1.17,5.01) | -0.09(-0.23,0.06) | 8.30(6.21,10.41) | 9.34(4.89,20.56) | 0.30(0.14,0.46) | 2.10(1.57,2.65) | 2.03(1.08,4.42) | -0.32(-0.46,-0.18) | 56.97(42.48,72.05) | 57.41(28.38,130.81) | -0.18(-0.33,-0.04) |
| **Republic of Zimbabwe** | 2.26(1.75,2.84) | 2.43(1.85,3.21) | 0.25(-0.03,0.52) | 9.49(7.40,11.84) | 9.84(7.55,12.97) | 0.17(-0.00,0.34) | 2.06(1.60,2.54) | 2.22(1.73,2.90) | 0.39(0.13,0.66) | 55.30(42.13,69.78) | 63.81(47.84,85.88) | 0.59(0.27,0.91) |
| **Romania** | 4.16(3.63,4.68) | 5.82(4.92,6.78) | 1.02(0.82,1.22) | 20.61(18.25,23.08) | 33.31(28.40,38.50) | 1.59(1.40,1.77) | 3.19(2.81,3.57) | 3.05(2.59,3.56) | -0.35(-0.54,-0.16) | 99.26(87.57,111.09) | 89.82(75.76,104.84) | -0.56(-0.74,-0.37) |
| **Russian Federation** | 4.52(4.40,4.63) | 2.88(2.54,3.17) | -2.06(-2.35,-1.78) | 21.58(20.96,22.17) | 15.52(13.81,16.97) | -1.56(-1.86,-1.26) | 3.35(3.25,3.43) | 1.67(1.49,1.84) | -2.93(-3.19,-2.67) | 105.85(102.84,108.72) | 48.57(43.17,53.45) | -3.23(-3.49,-2.96) |
| **Saint Kitts and Nevis** | 1.75(1.58,1.92) | 2.01(1.64,2.41) | 0.87(0.61,1.13) | 6.97(6.32,7.69) | 8.96(7.37,10.81) | 1.29(1.03,1.56) | 1.60(1.45,1.74) | 1.56(1.28,1.85) | 0.31(0.05,0.56) | 41.87(37.58,45.95) | 39.40(32.00,47.74) | 0.20(-0.07,0.48) |
| **Saint Lucia** | 2.74(2.57,2.95) | 2.67(2.15,3.25) | -0.22(-0.40,-0.04) | 11.50(10.76,12.38) | 12.47(10.11,15.16) | 0.16(0.02,0.30) | 2.43(2.28,2.60) | 2.06(1.67,2.50) | -0.68(-0.91,-0.45) | 61.15(57.19,65.53) | 51.78(41.65,63.40) | -0.63(-0.82,-0.43) |
| **Saint Vincent and the Grenadines** | 2.70(2.46,2.97) | 3.28(2.87,3.81) | 0.62(0.44,0.81) | 11.46(10.42,12.60) | 14.71(12.93,17.06) | 0.81(0.63,0.98) | 2.37(2.14,2.60) | 2.67(2.33,3.06) | 0.39(0.20,0.59) | 60.57(54.60,66.92) | 68.31(59.24,79.00) | 0.41(0.21,0.61) |
| **Slovak Republic** | 5.57(4.22,7.35) | 3.56(2.52,4.93) | -1.44(-1.51,-1.37) | 27.50(21.08,36.16) | 20.12(14.48,27.45) | -1.01(-1.07,-0.94) | 3.91(2.99,5.05) | 2.02(1.46,2.67) | -2.17(-2.25,-2.10) | 120.37(90.28,158.14) | 58.24(40.33,79.34) | -2.42(-2.51,-2.33) |
| **Socialist Republic of Viet Nam** | 1.55(1.21,2.02) | 2.24(1.65,2.98) | 1.25(1.20,1.30) | 6.86(5.37,8.84) | 11.58(8.54,15.42) | 1.79(1.74,1.84) | 1.29(1.02,1.66) | 1.34(0.99,1.74) | 0.13(0.05,0.20) | 34.43(26.62,45.17) | 35.88(26.06,48.19) | 0.19(0.14,0.25) |
| **Solomon Islands** | 0.86(0.54,1.22) | 0.75(0.53,1.04) | -0.44(-0.52,-0.36) | 3.16(2.04,4.46) | 3.00(2.20,4.02) | -0.19(-0.29,-0.09) | 0.84(0.54,1.18) | 0.70(0.50,0.95) | -0.61(-0.67,-0.55) | 20.08(12.07,28.73) | 17.19(12.16,23.67) | -0.50(-0.58,-0.43) |
| **State of Eritrea** | 2.15(1.52,2.94) | 1.63(1.15,2.23) | -1.12(-1.22,-1.03) | 7.83(5.68,10.67) | 6.68(4.83,8.96) | -0.78(-0.88,-0.68) | 2.05(1.44,2.79) | 1.53(1.09,2.07) | -1.17(-1.26,-1.08) | 60.74(42.14,83.29) | 43.29(29.84,59.42) | -1.33(-1.43,-1.23) |
| **State of Israel** | 2.16(1.95,2.43) | 1.77(1.55,2.02) | -0.96(-1.20,-0.72) | 12.53(11.35,14.03) | 11.63(10.24,13.12) | -0.46(-0.69,-0.24) | 1.22(1.10,1.35) | 0.68(0.60,0.78) | -2.31(-2.53,-2.09) | 31.27(28.26,34.64) | 17.34(15.37,19.81) | -2.29(-2.50,-2.08) |
| **State of Kuwait** | 2.61(2.30,2.93) | 0.92(0.73,1.14) | -2.42(-3.05,-1.79) | 14.09(12.48,15.81) | 5.77(4.68,7.10) | -1.99(-2.56,-1.41) | 1.49(1.32,1.66) | 0.39(0.32,0.48) | -3.50(-4.12,-2.88) | 39.83(35.23,44.45) | 9.08(7.31,11.35) | -3.90(-4.51,-3.28) |
| **State of Libya** | 4.11(2.98,5.61) | 4.80(3.43,6.51) | 0.85(0.65,1.04) | 19.46(14.40,26.24) | 25.44(18.51,34.34) | 1.23(1.01,1.45) | 3.20(2.34,4.37) | 2.99(2.17,4.04) | 0.01(-0.15,0.16) | 83.72(60.89,115.58) | 78.25(56.08,105.94) | -0.02(-0.17,0.12) |
| **State of Qatar** | 3.71(2.77,4.89) | 2.99(2.07,4.20) | -0.65(-1.24,-0.06) | 16.95(13.03,21.75) | 17.88(12.44,24.76) | 0.38(-0.13,0.89) | 2.81(2.07,3.75) | 1.31(0.91,1.84) | -2.64(-3.33,-1.95) | 63.99(47.84,84.58) | 29.60(20.52,41.58) | -2.54(-3.17,-1.90) |
| **Sultanate of Oman** | 1.06(0.75,1.45) | 0.86(0.65,1.16) | -0.52(-0.69,-0.35) | 5.04(3.66,6.72) | 4.87(3.72,6.41) | -0.04(-0.22,0.14) | 0.81(0.58,1.11) | 0.44(0.34,0.58) | -1.74(-1.91,-1.56) | 21.71(15.10,30.17) | 11.03(8.29,14.74) | -1.97(-2.16,-1.78) |
| **Swiss Confederation** | 3.52(3.16,3.87) | 1.83(1.60,2.07) | -1.90(-2.03,-1.78) | 21.61(19.53,23.73) | 12.80(11.38,14.32) | -1.54(-1.67,-1.40) | 1.40(1.29,1.51) | 0.53(0.47,0.59) | -2.86(-3.07,-2.65) | 38.72(35.63,42.04) | 13.60(12.07,15.18) | -3.16(-3.36,-2.96) |
| **Syrian Arab Republic** | 1.77(1.35,2.34) | 1.88(1.39,2.62) | -0.08(-0.24,0.08) | 8.42(6.70,10.79) | 10.22(7.72,14.16) | 0.32(0.17,0.48) | 1.50(1.15,1.98) | 1.16(0.86,1.59) | -1.13(-1.30,-0.95) | 37.93(28.83,49.08) | 27.50(20.05,38.52) | -1.35(-1.55,-1.16) |
| **Taiwan (Province of China)** | 2.10(1.95,2.26) | 1.70(1.48,1.89) | -0.99(-1.23,-0.75) | 11.26(10.44,12.13) | 10.67(9.39,11.87) | -0.41(-0.66,-0.17) | 1.17(1.09,1.26) | 0.59(0.53,0.65) | -2.62(-2.86,-2.37) | 30.72(28.69,33.03) | 15.62(13.94,17.36) | -2.52(-2.74,-2.30) |
| **Togolese Republic** | 1.06(0.80,1.37) | 1.35(0.97,1.83) | 1.03(0.92,1.15) | 4.24(3.27,5.39) | 5.53(4.03,7.38) | 1.07(0.97,1.17) | 1.01(0.77,1.31) | 1.23(0.88,1.67) | 0.89(0.76,1.02) | 26.26(19.57,33.90) | 32.37(22.99,43.78) | 0.93(0.81,1.06) |
| **Tokelau** | 0.69(0.50,0.98) | 0.58(0.41,0.82) | -0.74(-0.84,-0.64) | 2.77(2.05,3.87) | 2.63(1.91,3.68) | -0.34(-0.42,-0.26) | 0.64(0.46,0.92) | 0.47(0.33,0.66) | -1.20(-1.33,-1.07) | 14.72(10.39,21.25) | 10.60(7.50,15.11) | -1.22(-1.33,-1.12) |
| **Turkmenistan** | 2.77(2.57,2.99) | 1.27(0.97,1.68) | -2.90(-3.14,-2.66) | 12.16(11.32,13.03) | 6.32(4.99,8.08) | -2.44(-2.64,-2.25) | 2.45(2.27,2.63) | 1.03(0.79,1.34) | -3.20(-3.47,-2.93) | 71.89(66.77,76.98) | 29.87(22.96,39.32) | -3.23(-3.47,-2.98) |
| **Tuvalu** | 0.71(0.55,0.91) | 0.67(0.51,0.86) | -0.23(-0.33,-0.13) | 2.74(2.15,3.43) | 2.89(2.26,3.70) | 0.12(0.04,0.20) | 0.68(0.52,0.86) | 0.58(0.45,0.74) | -0.52(-0.62,-0.41) | 16.95(12.88,21.75) | 14.13(10.72,18.50) | -0.60(-0.68,-0.53) |
| **Ukraine** | 4.97(4.42,5.58) | 2.88(1.87,4.10) | -2.34(-2.54,-2.13) | 24.99(22.36,27.98) | 15.78(10.74,21.78) | -1.99(-2.17,-1.80) | 3.43(3.08,3.82) | 1.79(1.16,2.52) | -2.74(-2.97,-2.51) | 111.42(99.51,124.87) | 55.06(35.34,78.63) | -2.99(-3.24,-2.74) |
| **Union of the Comoros** | 1.89(1.27,2.49) | 1.41(1.04,1.95) | -1.25(-1.44,-1.05) | 7.45(5.00,9.73) | 5.97(4.43,8.14) | -1.00(-1.18,-0.82) | 1.80(1.23,2.39) | 1.30(0.95,1.80) | -1.36(-1.54,-1.17) | 49.54(33.00,66.26) | 34.71(25.12,48.73) | -1.49(-1.72,-1.27) |
| **United Arab Emirates** | 2.09(1.29,2.95) | 1.57(1.15,2.01) | 0.10(-0.35,0.54) | 10.00(6.64,13.70) | 8.40(6.38,10.50) | 0.21(-0.16,0.59) | 1.72(1.09,2.39) | 1.08(0.80,1.37) | -0.31(-0.80,0.18) | 43.83(27.07,62.26) | 23.29(17.05,29.82) | -1.16(-1.61,-0.70) |
| **United Kingdom of Great Britain and Northern Ireland** | 3.09(3.01,3.16) | 2.55(2.43,2.63) | -0.66(-0.77,-0.55) | 19.18(18.55,19.87) | 17.24(16.53,17.84) | -0.35(-0.46,-0.24) | 1.17(1.14,1.19) | 0.71(0.67,0.74) | -1.75(-1.90,-1.61) | 30.48(29.71,31.29) | 17.87(17.12,18.67) | -1.84(-1.97,-1.72) |
| **United Mexican States** | 2.21(2.14,2.27) | 1.00(0.85,1.16) | -2.97(-3.15,-2.79) | 8.75(8.49,9.01) | 4.60(3.92,5.33) | -2.56(-2.77,-2.35) | 2.07(2.00,2.13) | 0.79(0.68,0.91) | -3.43(-3.58,-3.28) | 46.15(44.87,47.40) | 18.17(15.44,21.13) | -3.40(-3.56,-3.24) |
| **United Republic of Tanzania** | 1.98(1.42,2.70) | 1.36(0.96,2.02) | -1.55(-1.66,-1.43) | 7.84(5.68,10.72) | 5.82(4.16,8.67) | -1.23(-1.32,-1.14) | 1.87(1.34,2.53) | 1.23(0.87,1.81) | -1.65(-1.76,-1.54) | 51.23(36.36,71.51) | 33.26(22.88,51.28) | -1.71(-1.82,-1.59) |
| **United States of America** | 4.07(3.94,4.17) | 2.87(2.72,2.98) | -1.48(-1.61,-1.36) | 25.39(24.60,26.13) | 18.94(18.06,19.71) | -1.28(-1.42,-1.14) | 1.35(1.29,1.38) | 0.78(0.74,0.82) | -2.06(-2.14,-1.98) | 37.06(35.91,38.15) | 20.40(19.50,21.33) | -2.23(-2.30,-2.15) |
| **United States Virgin Islands** | 2.12(1.69,2.74) | 1.44(1.02,1.94) | -1.20(-1.42,-0.98) | 9.66(7.80,12.23) | 6.95(5.01,9.23) | -1.07(-1.30,-0.84) | 1.68(1.35,2.15) | 1.02(0.73,1.37) | -1.55(-1.78,-1.32) | 43.72(34.83,56.28) | 26.52(18.88,35.63) | -1.47(-1.67,-1.27) |

**Table S2: Decomposition analysis results.**

| **Burden indicator** | **location_name** | **sex_name** | **Overall_Difference** | **Aging** | **Population** | **Epidemiological_Change** | **Aging_Percentage** | **Population_Percentage** | **Epidemiological_Change_Percentage** |
| --- | --- | --- | --- | --- | --- | --- | --- | --- | --- |
| Incidence | High SDI | Both | 7252.76 | 8058.35 | 17059.71 | -17865.29 | 111.11 | 235.22 | -246.32 |
|  | Low SDI | Both | 4893.26 | 7219.01 | 3856.8 | -6182.55 | 147.53 | 78.82 | -126.35 |
|  | Global | Both | 75707.97 | 61774.17 | 63683.23 | -49749.43 | 81.6 | 84.12 | -65.71 |
|  | Middle SDI | Both | 32595.65 | 19873.83 | 15901.81 | -3179.99 | 60.97 | 48.79 | -9.76 |
|  | High-middle SDI | Both | 11157.71 | 12653.62 | 17986.63 | -19482.55 | 113.41 | 161.2 | -174.61 |
|  | Low-middle SDI | Both | 19734.37 | 12044.76 | 10203.6 | -2513.99 | 61.03 | 51.7 | -12.74 |
|  | High SDI | Male | 5051.69 | 9982.05 | 14558.99 | -19489.35 | 197.6 | 288.2 | -385.8 |
|  | Low SDI | Male | 3925.96 | 6464.36 | 3283.27 | -5821.67 | 164.66 | 83.63 | -148.29 |
|  | Global | Male | 61910.1 | 56887.33 | 54854.21 | -49831.45 | 91.89 | 88.6 | -80.49 |
|  | Middle SDI | Male | 28047.77 | 17304.54 | 13542.88 | -2799.65 | 61.7 | 48.29 | -9.98 |
|  | High-middle SDI | Male | 8301.41 | 13809.76 | 16266.59 | -21774.93 | 166.35 | 195.95 | -262.3 |
|  | Low-middle SDI | Male | 16522.15 | 9279.4 | 8626.64 | -1383.9 | 56.16 | 52.21 | -8.38 |
|  | High SDI | Female | 2201.08 | 828.12 | 2508.98 | -1136.02 | 37.62 | 113.99 | -51.61 |
|  | Low SDI | Female | 967.3 | 1099.81 | 624.18 | -756.68 | 113.7 | 64.53 | -78.23 |
|  | Global | Female | 13797.87 | 7825.64 | 8519.23 | -2547 | 56.72 | 61.74 | -18.46 |
|  | Middle SDI | Female | 4547.88 | 3531.93 | 2525.68 | -1509.73 | 77.66 | 55.54 | -33.2 |
|  | High-middle SDI | Female | 2856.29 | 1070.58 | 1686.71 | 99.01 | 37.48 | 59.05 | 3.47 |
|  | Low-middle SDI | Female | 3212.22 | 1857.32 | 1478.35 | -123.44 | 57.82 | 46.02 | -3.84 |
| Prevalence | Low SDI | Both | 23108.85 | 16044.89 | 14245.73 | -7181.77 | 69.43 | 61.65 | -31.08 |
|  | Low-middle SDI | Both | 96386.81 | 65692.57 | 47923.44 | -17229.21 | 68.16 | 49.72 | -17.88 |
|  | Global | Both | 475151.28 | 333967.9 | 362167.98 | -220984.6 | 70.29 | 76.22 | -46.51 |
|  | High-middle SDI | Both | 95395.64 | 101868.23 | 98338.9 | -104811.49 | 106.78 | 103.09 | -109.87 |
|  | High SDI | Both | 73723.88 | 84430.08 | 106255.69 | -116961.89 | 114.52 | 144.13 | -158.65 |
|  | Middle SDI | Both | 186028.43 | 100522.59 | 71809.51 | 13696.32 | 54.04 | 38.6 | 7.36 |
|  | Low SDI | Male | 18474.51 | 9922.23 | 11952.3 | -3400.01 | 53.71 | 64.7 | -18.4 |
|  | Low-middle SDI | Male | 80477.48 | 47461.19 | 40813.88 | -7797.59 | 58.97 | 50.71 | -9.69 |
|  | Global | Male | 390552.7 | 260782.07 | 319512.06 | -189741.43 | 66.77 | 81.81 | -48.58 |
|  | High-middle SDI | Male | 75989.79 | 102541.09 | 90368.34 | -116919.64 | 134.94 | 118.92 | -153.86 |
|  | High SDI | Male | 56255.01 | 92554.98 | 91336.64 | -127636.62 | 164.53 | 162.36 | -226.89 |
|  | Middle SDI | Male | 158929.8 | 82683.94 | 59927.7 | 16318.16 | 52.03 | 37.71 | 10.27 |
|  | Low SDI | Female | 4634.34 | 3141.21 | 2443.06 | -949.93 | 67.78 | 52.72 | -20.5 |
|  | Low-middle SDI | Female | 15909.33 | 9996.65 | 7220.93 | -1308.24 | 62.84 | 45.39 | -8.22 |
|  | Global | Female | 84598.58 | 36197.3 | 50572.69 | -2171.41 | 42.79 | 59.78 | -2.57 |
|  | High-middle SDI | Female | 19405.85 | 8585.65 | 9591.21 | 1228.99 | 44.24 | 49.42 | 6.33 |
|  | High SDI | Female | 17468.88 | 9906.07 | 16282.77 | -8719.97 | 56.71 | 93.21 | -49.92 |
|  | Middle SDI | Female | 27098.63 | 16026.26 | 11395.7 | -323.33 | 59.14 | 42.05 | -1.19 |
| Deaths | Global | Both | 31461.9 | 26131.61 | 55729.53 | -50399.24 | 83.06 | 177.13 | -160.19 |
|  | Low-middle SDI | Both | 14711.94 | 7531.55 | 12034.29 | -4853.9 | 51.19 | 81.8 | -32.99 |
|  | Low SDI | Both | 3966.59 | 2004.23 | 3749.61 | -1787.26 | 50.53 | 94.53 | -45.06 |
|  | High SDI | Both | -1823.93 | 1291.77 | 9497.11 | -12612.8 | -70.82 | -520.7 | 691.52 |
|  | Middle SDI | Both | 16080.08 | 12650.08 | 14915.84 | -11485.84 | 78.67 | 92.76 | -71.43 |
|  | High-middle SDI | Both | -1492.7 | 4312.26 | 15295.73 | -21100.69 | -288.89 | -1024.7 | 1413.59 |
|  | Global | Male | 25131.06 | 25424.44 | 48163.34 | -48456.72 | 101.17 | 191.65 | -192.82 |
|  | Low-middle SDI | Male | 12288.8 | 5532.24 | 10223.8 | -3467.25 | 45.02 | 83.2 | -28.21 |
|  | Low SDI | Male | 3189.07 | 1477.15 | 3113.51 | -1401.59 | 46.32 | 97.63 | -43.95 |
|  | High SDI | Male | -2047.87 | 2618.59 | 8232.72 | -12899.19 | -127.87 | -402.01 | 629.88 |
|  | Middle SDI | Male | 13903.53 | 10450.43 | 12453.76 | -9000.65 | 75.16 | 89.57 | -64.74 |
|  | High-middle SDI | Male | -2216.96 | 5590.76 | 13904.02 | -21711.74 | -252.18 | -627.17 | 979.35 |
|  | Global | Female | 6330.85 | 3185.64 | 7372.54 | -4227.34 | 50.32 | 116.45 | -66.77 |
|  | Low-middle SDI | Female | 2423.15 | 1178.92 | 1701.29 | -457.06 | 48.65 | 70.21 | -18.86 |
|  | Low SDI | Female | 777.52 | 364.2 | 607.76 | -194.44 | 46.84 | 78.17 | -25.01 |
|  | High SDI | Female | 223.95 | 61.63 | 1310.44 | -1148.12 | 27.52 | 585.16 | -512.68 |
|  | Middle SDI | Female | 2176.55 | 2052.23 | 2366.75 | -2242.44 | 94.29 | 108.74 | -103.03 |
|  | High-middle SDI | Female | 724.26 | 348.57 | 1407.98 | -1032.29 | 48.13 | 194.4 | -142.53 |
| DAlYs | Global | Both | 667466.55 | 1034308.24 | 1135982.7 | -1502824.4 | 154.96 | 170.19 | -225.15 |
|  | Middle SDI | Both | 386296.11 | 414898.5 | 304543.77 | -333146.15 | 107.4 | 78.84 | -86.24 |
|  | High-middle SDI | Both | -128187.2 | 199526.19 | 310298.68 | -638012.07 | -155.65 | -242.07 | 497.72 |
|  | Low SDI | Both | 112075.99 | 88915.36 | 81303.91 | -58143.28 | 79.33 | 72.54 | -51.88 |
|  | Low-middle SDI | Both | 397105.04 | 291240.97 | 256735.8 | -150871.74 | 73.34 | 64.65 | -37.99 |
|  | High SDI | Both | -99970.08 | 72857.75 | 177371.5 | -350199.32 | -72.88 | -177.42 | 350.3 |
|  | Global | Male | 513032.61 | 944710.15 | 982290.74 | -1413968.27 | 184.14 | 191.47 | -275.61 |
|  | Middle SDI | Male | 337356.25 | 342477 | 255109.55 | -260230.3 | 101.52 | 75.62 | -77.14 |
|  | High-middle SDI | Male | -140276.42 | 218394.61 | 283876.4 | -642547.43 | -155.69 | -202.37 | 458.06 |
|  | Low SDI | Male | 88834 | 68197.35 | 66750.83 | -46114.18 | 76.77 | 75.14 | -51.91 |
|  | Low-middle SDI | Male | 329134.63 | 225115.54 | 216350.89 | -112331.79 | 68.4 | 65.73 | -34.13 |
|  | High SDI | Male | -102058.55 | 92420.4 | 154492.79 | -348971.74 | -90.56 | -151.38 | 341.93 |
|  | Global | Female | 154433.93 | 123986.23 | 147372.32 | -116924.61 | 80.28 | 95.43 | -75.71 |
|  | Middle SDI | Female | 48939.86 | 63100.05 | 46926.74 | -61086.93 | 128.93 | 95.89 | -124.82 |
|  | High-middle SDI | Female | 12089.22 | 14435.49 | 26006.04 | -28352.32 | 119.41 | 215.12 | -234.53 |
|  | Low SDI | Female | 23241.99 | 16248.37 | 13863.76 | -6870.14 | 69.91 | 59.65 | -29.56 |
|  | Low-middle SDI | Female | 67970.4 | 44730.97 | 37794.23 | -14554.8 | 65.81 | 55.6 | -21.41 |
|  | High SDI | Female | 2088.47 | 6772.12 | 23244.8 | -27928.45 | 324.26 | 1113 | -1337.27 |

**Table S3. Predictive analysis of age - standardized LC until 2050 (ASR).**

| **Burden indicator** | **val** | **sd** | **Time** | **group** | **low_95** | **up_95** |
| --- | --- | --- | --- | --- | --- | --- |
| Incidence | 2.886256787 | 0.047931481 | 1990 | ASR | 2.792311085 | 2.980202489 |
|  | 2.792641854 | 0.038008976 | 1991 | ASR | 2.718144261 | 2.867139448 |
|  | 2.793630999 | 0.038000526 | 1992 | ASR | 2.719149968 | 2.868112031 |
|  | 2.805776602 | 0.037983045 | 1993 | ASR | 2.731329832 | 2.880223371 |
|  | 2.800020688 | 0.037639992 | 1994 | ASR | 2.726246304 | 2.873795072 |
|  | 2.789161101 | 0.037487747 | 1995 | ASR | 2.715685118 | 2.862637085 |
|  | 2.750131549 | 0.036949706 | 1996 | ASR | 2.677710126 | 2.822552973 |
|  | 2.699283388 | 0.036219913 | 1997 | ASR | 2.628292358 | 2.770274418 |
|  | 2.647702215 | 0.035556983 | 1998 | ASR | 2.578010528 | 2.717393902 |
|  | 2.590241187 | 0.034865292 | 1999 | ASR | 2.521905214 | 2.65857716 |
|  | 2.545968934 | 0.034417045 | 2000 | ASR | 2.478511526 | 2.613426341 |
|  | 2.494985419 | 0.033822631 | 2001 | ASR | 2.428693063 | 2.561277776 |
|  | 2.384080094 | 0.026538449 | 2002 | ASR | 2.332064734 | 2.436095455 |
|  | 2.353124246 | 0.026296866 | 2003 | ASR | 2.30158239 | 2.404666103 |
|  | 2.318006947 | 0.02605204 | 2004 | ASR | 2.266944948 | 2.369068946 |
|  | 2.246245756 | 0.018854442 | 2005 | ASR | 2.209291049 | 2.283200463 |
|  | 2.212517238 | 0.018531087 | 2006 | ASR | 2.176196308 | 2.248838169 |
|  | 2.187842821 | 0.018328317 | 2007 | ASR | 2.151919319 | 2.223766323 |
|  | 2.180280447 | 0.018179836 | 2008 | ASR | 2.144647969 | 2.215912926 |
|  | 2.150861406 | 0.017992705 | 2009 | ASR | 2.115595705 | 2.186127107 |
|  | 2.1386941 | 0.01779005 | 2010 | ASR | 2.103825602 | 2.173562599 |
|  | 2.13059326 | 0.017661553 | 2011 | ASR | 2.095976615 | 2.165209904 |
|  | 2.125926887 | 0.017514374 | 2012 | ASR | 2.091598713 | 2.160255061 |
|  | 2.100759321 | 0.017364003 | 2013 | ASR | 2.066725876 | 2.134792767 |
|  | 2.077363613 | 0.017217576 | 2014 | ASR | 2.043617164 | 2.111110062 |
|  | 2.073668095 | 0.017067188 | 2015 | ASR | 2.040216407 | 2.107119784 |
|  | 2.07001203 | 0.016982313 | 2016 | ASR | 2.036726695 | 2.103297364 |
|  | 2.050699877 | 0.016756641 | 2017 | ASR | 2.01785686 | 2.083542894 |
|  | 2.042217298 | 0.016590218 | 2018 | ASR | 2.00970047 | 2.074734126 |
|  | 2.033618094 | 0.016582582 | 2019 | ASR | 2.001116234 | 2.066119955 |
|  | 2.022963565 | 0.01647906 | 2020 | ASR | 1.990664609 | 2.055262522 |
|  | 2.012611541 | 0.016590431 | 2021 | ASR | 1.980094296 | 2.045128786 |
|  | 2.364850209 | 0.097630211 | 2022 | ASR | 2.173494996 | 2.556205421 |
|  | 2.329173665 | 0.110623724 | 2023 | ASR | 2.112351166 | 2.545996163 |
|  | 2.294546836 | 0.118108281 | 2024 | ASR | 2.063054605 | 2.526039067 |
|  | 2.261075329 | 0.122770278 | 2025 | ASR | 2.020445584 | 2.501705074 |
|  | 2.228953632 | 0.126034443 | 2026 | ASR | 1.981926123 | 2.475981141 |
|  | 2.197351132 | 0.128233479 | 2027 | ASR | 1.946013514 | 2.44868875 |
|  | 2.166954367 | 0.130065234 | 2028 | ASR | 1.912026508 | 2.421882226 |
|  | 2.137611629 | 0.131467528 | 2029 | ASR | 1.879935273 | 2.395287984 |
|  | 2.109808546 | 0.132890375 | 2030 | ASR | 1.84934341 | 2.370273682 |
|  | 2.083384623 | 0.134121182 | 2031 | ASR | 1.820507106 | 2.346262139 |
|  | 2.058016668 | 0.135365528 | 2032 | ASR | 1.792700232 | 2.323333103 |
|  | 2.033647449 | 0.136489314 | 2033 | ASR | 1.766128394 | 2.301166505 |
|  | 2.010432715 | 0.137608438 | 2034 | ASR | 1.740720177 | 2.280145253 |
|  | 1.988483516 | 0.138715743 | 2035 | ASR | 1.71660066 | 2.260366373 |
|  | 1.967906005 | 0.139758565 | 2036 | ASR | 1.693979217 | 2.241832793 |
|  | 1.948326953 | 0.140789469 | 2037 | ASR | 1.672379594 | 2.224274312 |
|  | 1.929425382 | 0.141656502 | 2038 | ASR | 1.651778637 | 2.207072127 |
|  | 1.911338891 | 0.142533084 | 2039 | ASR | 1.631974046 | 2.190703736 |
|  | 1.894003788 | 0.1432755 | 2040 | ASR | 1.613183808 | 2.174823767 |
|  | 1.87781876 | 0.144074151 | 2041 | ASR | 1.595433424 | 2.160204096 |
|  | 1.862326382 | 0.144780259 | 2042 | ASR | 1.578557073 | 2.14609569 |
|  | 1.847334518 | 0.145510986 | 2043 | ASR | 1.562132985 | 2.13253605 |
|  | 1.832658186 | 0.146177931 | 2044 | ASR | 1.54614944 | 2.119166931 |
|  | 1.818432248 | 0.14687929 | 2045 | ASR | 1.530548839 | 2.106315657 |
|  | 1.804849686 | 0.147622299 | 2046 | ASR | 1.51550998 | 2.094189392 |
|  | 1.791745621 | 0.148448424 | 2047 | ASR | 1.500786709 | 2.082704532 |
|  | 1.778820873 | 0.149367606 | 2048 | ASR | 1.486060365 | 2.071581382 |
|  | 1.765954507 | 0.150347383 | 2049 | ASR | 1.471273637 | 2.060635377 |
|  | 1.753235481 | 0.151442886 | 2050 | ASR | 1.456407424 | 2.050063538 |
| Prevalence | 22.42281028 | 8.45387E-07 | 1990 | ASR | 22.42280863 | 22.42281194 |
|  | 22.43130119 | 8.54135E-07 | 1991 | ASR | 22.43129952 | 22.43130287 |
|  | 22.43553204 | 8.66117E-07 | 1992 | ASR | 22.43553034 | 22.43553374 |
|  | 23.17275645 | 8.78592E-07 | 1993 | ASR | 23.17275473 | 23.17275817 |
|  | 23.05296729 | 8.89506E-07 | 1994 | ASR | 23.05296555 | 23.05296903 |
|  | 23.02120914 | 8.9834E-07 | 1995 | ASR | 23.02120738 | 23.0212109 |
|  | 21.79162339 | 9.02693E-07 | 1996 | ASR | 21.79162162 | 21.79162516 |
|  | 21.43746822 | 9.02222E-07 | 1997 | ASR | 21.43746645 | 21.43746999 |
|  | 21.09799456 | 9.00719E-07 | 1998 | ASR | 21.0979928 | 21.09799633 |
|  | 20.85635733 | 8.97549E-07 | 1999 | ASR | 20.85635557 | 20.85635909 |
|  | 20.5464508 | 8.9474E-07 | 2000 | ASR | 20.54644905 | 20.54645256 |
|  | 20.14929419 | 8.91328E-07 | 2001 | ASR | 20.14929244 | 20.14929593 |
|  | 19.79534674 | 8.90281E-07 | 2002 | ASR | 19.79534499 | 19.79534848 |
|  | 19.53756688 | 8.92368E-07 | 2003 | ASR | 19.53756513 | 19.53756862 |
|  | 19.30471406 | 8.96655E-07 | 2004 | ASR | 19.3047123 | 19.30471582 |
|  | 19.25059647 | 9.03283E-07 | 2005 | ASR | 19.2505947 | 19.25059824 |
|  | 19.08254401 | 9.08657E-07 | 2006 | ASR | 19.08254223 | 19.08254579 |
|  | 18.71246763 | 9.14762E-07 | 2007 | ASR | 18.71246584 | 18.71246942 |
|  | 16.5521797 | 9.21543E-07 | 2008 | ASR | 16.5521779 | 16.55218151 |
|  | 16.3577018 | 9.24102E-07 | 2009 | ASR | 16.35769999 | 16.35770362 |
|  | 16.49740255 | 9.24856E-07 | 2010 | ASR | 16.49740073 | 16.49740436 |
|  | 16.31186212 | 9.23847E-07 | 2011 | ASR | 16.31186031 | 16.31186393 |
|  | 16.99559474 | 9.18482E-07 | 2012 | ASR | 16.99559294 | 16.99559654 |
|  | 16.67735674 | 9.08593E-07 | 2013 | ASR | 16.67735496 | 16.67735852 |
|  | 16.4928815 | 8.94157E-07 | 2014 | ASR | 16.49287975 | 16.49288325 |
|  | 16.3499375 | 8.76114E-07 | 2015 | ASR | 16.34993578 | 16.34993922 |
|  | 16.31280751 | 8.56699E-07 | 2016 | ASR | 16.31280583 | 16.31280919 |
|  | 14.28893352 | 8.3349E-07 | 2017 | ASR | 14.28893189 | 14.28893515 |
|  | 13.67474827 | 8.10989E-07 | 2018 | ASR | 13.67474668 | 13.67474986 |
|  | 13.64350458 | 7.91742E-07 | 2019 | ASR | 13.64350303 | 13.64350613 |
|  | 13.79192436 | 7.7407E-07 | 2020 | ASR | 13.79192284 | 13.79192587 |
|  | 13.53261724 | 7.62969E-07 | 2021 | ASR | 13.53261575 | 13.53261874 |
|  | 5.66955934 | 6.61491E-07 | 2022 | ASR | 5.669558044 | 5.669560637 |
|  | 5.512429299 | 6.64522E-07 | 2023 | ASR | 5.512427997 | 5.512430602 |
|  | 4.517130301 | 0.008557776 | 2024 | ASR | 4.50035706 | 4.533903542 |
|  | 4.223289999 | 0.007649014 | 2025 | ASR | 4.208297931 | 4.238282066 |
|  | 22.13683075 | 0.007094653 | 2026 | ASR | 22.12292523 | 22.15073627 |
|  | 46.22040395 | 0.006797593 | 2027 | ASR | 46.20708067 | 46.23372723 |
|  | 59.72935189 | 0.023398689 | 2028 | ASR | 59.68349046 | 59.77521332 |
|  | 64.35597199 | 0.021333319 | 2029 | ASR | 64.31415868 | 64.39778529 |
|  | 57.38872272 | 0.018331085 | 2030 | ASR | 57.3527938 | 57.42465165 |
|  | 38.965261 | 0.014831725 | 2031 | ASR | 38.93619082 | 38.99433118 |
|  | 31.00620918 | 2.470038177 | 2032 | ASR | 26.16493435 | 35.84748401 |
|  | 24.13070589 | 3.995321229 | 2033 | ASR | 16.29987629 | 31.9615355 |
|  | 23.52060668 | 4.2378479 | 2034 | ASR | 15.21442479 | 31.82678856 |
|  | 17.62404091 | 4.810556527 | 2035 | ASR | 8.195350121 | 27.05273171 |
|  | 16.83437798 | 4.884047009 | 2036 | ASR | 7.26164584 | 26.40711012 |
|  | 14.22927774 | 5.112450915 | 2037 | ASR | 4.208873951 | 24.24968154 |
|  | 14.03206464 | 5.170712166 | 2038 | ASR | 3.897468797 | 24.16666049 |
|  | 13.98493215 | 5.227254404 | 2039 | ASR | 3.739513517 | 24.23035078 |
|  | 13.95352116 | 5.28979227 | 2040 | ASR | 3.58552831 | 24.32151401 |
|  | 13.91618676 | 5.350905024 | 2041 | ASR | 3.428412918 | 24.40396061 |
|  | 13.89497518 | 5.411922488 | 2042 | ASR | 3.287607104 | 24.50234326 |
|  | 13.88278578 | 5.475598043 | 2043 | ASR | 3.150613614 | 24.61495794 |
|  | 13.87461681 | 5.536235736 | 2044 | ASR | 3.02359477 | 24.72563885 |
|  | 13.86297465 | 5.590225907 | 2045 | ASR | 2.906131875 | 24.81981743 |
|  | 13.80310231 | 5.623979011 | 2046 | ASR | 2.78010345 | 24.82610117 |
|  | 13.77850983 | 5.664931003 | 2047 | ASR | 2.675245066 | 24.8817746 |
|  | 13.74699557 | 5.689039895 | 2048 | ASR | 2.596477372 | 24.89751376 |
|  | 13.72575498 | 5.714620957 | 2049 | ASR | 2.525097903 | 24.92641206 |
|  | 13.66996809 | 5.701343356 | 2050 | ASR | 2.495335109 | 24.84460107 |
| Deaths | 2.229247821 | 0.037749378 | 1990 | ASR | 2.155259041 | 2.303236601 |
|  | 2.214941417 | 0.036599451 | 1991 | ASR | 2.143206493 | 2.286676342 |
|  | 2.206821999 | 0.036434271 | 1992 | ASR | 2.135410827 | 2.27823317 |
|  | 2.205282869 | 0.036407784 | 1993 | ASR | 2.133923612 | 2.276642126 |
|  | 2.18999969 | 0.036193516 | 1994 | ASR | 2.119060398 | 2.260938981 |
|  | 2.160928428 | 0.035749942 | 1995 | ASR | 2.090858542 | 2.230998314 |
|  | 2.112589547 | 0.034988704 | 1996 | ASR | 2.044011687 | 2.181167407 |
|  | 2.064655391 | 0.034225389 | 1997 | ASR | 1.997573629 | 2.131737152 |
|  | 2.018398519 | 0.033487622 | 1998 | ASR | 1.95276278 | 2.084034258 |
|  | 1.969825188 | 0.032698849 | 1999 | ASR | 1.905735445 | 2.033914932 |
|  | 1.933146121 | 0.032110382 | 2000 | ASR | 1.870209772 | 1.996082471 |
|  | 1.890645767 | 0.031418743 | 2001 | ASR | 1.829065031 | 1.952226502 |
|  | 1.847709242 | 0.030699286 | 2002 | ASR | 1.787538641 | 1.907879843 |
|  | 1.811770774 | 0.030102064 | 2003 | ASR | 1.75277073 | 1.870770819 |
|  | 1.768747973 | 0.029388446 | 2004 | ASR | 1.711146619 | 1.826349328 |
|  | 1.734618139 | 0.02881727 | 2005 | ASR | 1.67813629 | 1.791099988 |
|  | 1.686837673 | 0.028015916 | 2006 | ASR | 1.631926478 | 1.741748869 |
|  | 1.656225978 | 0.027481438 | 2007 | ASR | 1.602362359 | 1.710089598 |
|  | 1.64091318 | 0.027197858 | 2008 | ASR | 1.587605378 | 1.694220981 |
|  | 1.609894921 | 0.026665316 | 2009 | ASR | 1.557630902 | 1.662158941 |
|  | 1.594043384 | 0.026387593 | 2010 | ASR | 1.542323701 | 1.645763067 |
|  | 1.575188472 | 0.026045371 | 2011 | ASR | 1.524139545 | 1.626237398 |
|  | 1.555363721 | 0.025667934 | 2012 | ASR | 1.50505457 | 1.605672872 |
|  | 1.529575938 | 0.025198936 | 2013 | ASR | 1.480186023 | 1.578965853 |
|  | 1.506971637 | 0.024788498 | 2014 | ASR | 1.45838618 | 1.555557094 |
|  | 1.49320362 | 0.024535757 | 2015 | ASR | 1.445113537 | 1.541293703 |
|  | 1.483054926 | 0.024331674 | 2016 | ASR | 1.435364846 | 1.530745007 |
|  | 1.464088111 | 0.023985993 | 2017 | ASR | 1.417075565 | 1.511100658 |
|  | 1.452561526 | 0.023758537 | 2018 | ASR | 1.405994794 | 1.499128259 |
|  | 1.440096323 | 0.023497374 | 2019 | ASR | 1.39404147 | 1.486151177 |
|  | 1.422984391 | 0.023183451 | 2020 | ASR | 1.377544827 | 1.468423955 |
|  | 1.40438088 | 0.023263392 | 2021 | ASR | 1.358784632 | 1.449977127 |
|  | 1.366170346 | 0.04032156 | 2022 | ASR | 1.287140088 | 1.445200604 |
|  | 1.338924476 | 0.045818618 | 2023 | ASR | 1.249119984 | 1.428728968 |
|  | 1.312315571 | 0.048867113 | 2024 | ASR | 1.216536029 | 1.408095114 |
|  | 1.286436881 | 0.050774428 | 2025 | ASR | 1.186919002 | 1.385954759 |
|  | 1.261290291 | 0.052171924 | 2026 | ASR | 1.159033319 | 1.363547262 |
|  | 1.236620441 | 0.053196607 | 2027 | ASR | 1.13235509 | 1.340885792 |
|  | 1.212776768 | 0.054127154 | 2028 | ASR | 1.106687546 | 1.31886599 |
|  | 1.189673174 | 0.054904717 | 2029 | ASR | 1.082059928 | 1.297286419 |
|  | 1.167542291 | 0.055709947 | 2030 | ASR | 1.058350795 | 1.276733788 |
|  | 1.14614843 | 0.056435507 | 2031 | ASR | 1.035534835 | 1.256762024 |
|  | 1.125390488 | 0.057172319 | 2032 | ASR | 1.013332742 | 1.237448234 |
|  | 1.105268954 | 0.057851862 | 2033 | ASR | 0.991879304 | 1.218658604 |
|  | 1.085872989 | 0.058514701 | 2034 | ASR | 0.971184175 | 1.200561802 |
|  | 1.067256317 | 0.059155543 | 2035 | ASR | 0.951311453 | 1.183201182 |
|  | 1.049360527 | 0.059753515 | 2036 | ASR | 0.932243637 | 1.166477416 |
|  | 1.032045582 | 0.06033516 | 2037 | ASR | 0.913788669 | 1.150302496 |
|  | 1.015228808 | 0.06084681 | 2038 | ASR | 0.89596906 | 1.134488557 |
|  | 0.998990581 | 0.061349817 | 2039 | ASR | 0.878744939 | 1.119236223 |
|  | 0.983295168 | 0.061797062 | 2040 | ASR | 0.862172927 | 1.104417409 |
|  | 0.968210689 | 0.06225156 | 2041 | ASR | 0.846197631 | 1.090223747 |
|  | 0.953537833 | 0.062668637 | 2042 | ASR | 0.830707305 | 1.076368361 |
|  | 0.939268269 | 0.063090204 | 2043 | ASR | 0.81561147 | 1.062925069 |
|  | 0.925373909 | 0.063492907 | 2044 | ASR | 0.800927812 | 1.049820007 |
|  | 0.911926537 | 0.063912314 | 2045 | ASR | 0.786658402 | 1.037194672 |
|  | 0.898941064 | 0.064350294 | 2046 | ASR | 0.772814488 | 1.02506764 |
|  | 0.886328932 | 0.064820877 | 2047 | ASR | 0.759280013 | 1.01337785 |
|  | 0.874009133 | 0.065327492 | 2048 | ASR | 0.745967249 | 1.002051017 |
|  | 0.861972468 | 0.065867746 | 2049 | ASR | 0.732871686 | 0.99107325 |
|  | 0.850268569 | 0.066463042 | 2050 | ASR | 0.720001007 | 0.980536132 |
| DALYs | 74.12591019 | 0.019252233 | 1990 | ASR | 74.08817581 | 74.16364457 |
|  | 73.54008117 | 0.018919009 | 1991 | ASR | 73.50299991 | 73.57716243 |
|  | 73.55533845 | 0.018679318 | 1992 | ASR | 73.51872698 | 73.59194991 |
|  | 67.42428228 | 0.018463034 | 1993 | ASR | 67.38809473 | 67.46046982 |
|  | 66.72728652 | 0.018273475 | 1994 | ASR | 66.69147051 | 66.76310253 |
|  | 65.61825191 | 0.017922957 | 1995 | ASR | 65.58312292 | 65.65338091 |
|  | 63.82485711 | 0.011042 | 1996 | ASR | 63.80321479 | 63.84649943 |
|  | 62.14188395 | 0.011045025 | 1997 | ASR | 62.1202357 | 62.1635322 |
|  | 63.03828629 | 0.011085539 | 1998 | ASR | 63.01655863 | 63.06001395 |
|  | 61.70891158 | 0.011097789 | 1999 | ASR | 61.68715991 | 61.73066325 |
|  | 60.36788794 | 0.01107173 | 2000 | ASR | 60.34618735 | 60.38958853 |
|  | 57.92724462 | 0.010923619 | 2001 | ASR | 57.90583432 | 57.94865491 |
|  | 56.43971264 | 0.010824333 | 2002 | ASR | 56.41849695 | 56.46092834 |
|  | 60.06538204 | 0.01075211 | 2003 | ASR | 60.0443079 | 60.08645617 |
|  | 58.53111485 | 0.010697699 | 2004 | ASR | 58.51014736 | 58.55208234 |
|  | 57.30440486 | 0.010644334 | 2005 | ASR | 57.28354196 | 57.32526775 |
|  | 53.09170048 | 0.010565841 | 2006 | ASR | 53.07099143 | 53.11240953 |
|  | 53.40134883 | 0.010602122 | 2007 | ASR | 53.38056867 | 53.42212899 |
|  | 52.75284668 | 0.0106836 | 2008 | ASR | 52.73190682 | 52.77378653 |
|  | 51.48388129 | 0.010684055 | 2009 | ASR | 51.46294054 | 51.50482204 |
|  | 50.84368078 | 0.010778505 | 2010 | ASR | 50.82255491 | 50.86480665 |
|  | 50.10645358 | 0.01077 | 2011 | ASR | 50.08534438 | 50.12756278 |
|  | 47.21574774 | 0.010774582 | 2012 | ASR | 47.19462956 | 47.23686592 |
|  | 46.21634596 | 0.010804233 | 2013 | ASR | 46.19516966 | 46.23752225 |
|  | 45.18644169 | 0.010749695 | 2014 | ASR | 45.16537229 | 45.2075111 |
|  | 45.19181514 | 0.010784313 | 2015 | ASR | 45.17067789 | 45.2129524 |
|  | 43.00534096 | 0.010906159 | 2016 | ASR | 42.98396489 | 43.02671704 |
|  | 42.35564469 | 0.010969204 | 2017 | ASR | 42.33414505 | 42.37714433 |
|  | 41.9149282 | 0.010960783 | 2018 | ASR | 41.89344506 | 41.93641133 |
|  | 43.29607214 | 0.011122453 | 2019 | ASR | 43.27427214 | 43.31787215 |
|  | 42.66587133 | 5.14587E-07 | 2020 | ASR | 42.66587033 | 42.66587234 |
|  | 40.43760903 | 5.1032E-07 | 2021 | ASR | 40.43760803 | 40.43761003 |
|  | 36.48888262 | 1.581204457 | 2022 | ASR | 33.38972189 | 39.58804336 |
|  | 35.83015434 | 1.785316733 | 2023 | ASR | 32.33093355 | 39.32937514 |
|  | 35.18806807 | 1.934039049 | 2024 | ASR | 31.39735154 | 38.97878461 |
|  | 34.56663275 | 2.048623528 | 2025 | ASR | 30.55133064 | 38.58193487 |
|  | 33.96912672 | 2.13662916 | 2026 | ASR | 29.78133357 | 38.15691987 |
|  | 33.39676612 | 2.207657491 | 2027 | ASR | 29.06975743 | 37.7237748 |
|  | 32.84611546 | 2.263969397 | 2028 | ASR | 28.40873544 | 37.28349548 |
|  | 32.31543407 | 2.310744806 | 2029 | ASR | 27.78637425 | 36.84449389 |
|  | 31.8027528 | 2.348816174 | 2030 | ASR | 27.1990731 | 36.4064325 |
|  | 31.31450823 | 2.381633028 | 2031 | ASR | 26.6465075 | 35.98250897 |
|  | 30.8479021 | 2.409489817 | 2032 | ASR | 26.12530206 | 35.57050214 |
|  | 30.39998305 | 2.434511542 | 2033 | ASR | 25.62834043 | 35.17162567 |
|  | 29.96766953 | 2.457084708 | 2034 | ASR | 25.1517835 | 34.78355556 |
|  | 29.55102875 | 2.478279431 | 2035 | ASR | 24.69360107 | 34.40845644 |
|  | 29.1549664 | 2.498028187 | 2036 | ASR | 24.25883115 | 34.05110165 |
|  | 28.77590165 | 2.516348318 | 2037 | ASR | 23.84385895 | 33.70794436 |
|  | 28.40870963 | 2.533486523 | 2038 | ASR | 23.44307604 | 33.37434321 |
|  | 28.05241363 | 2.55092655 | 2039 | ASR | 23.0525976 | 33.05222967 |
|  | 27.70730713 | 2.568707045 | 2040 | ASR | 22.67264132 | 32.74197294 |
|  | 27.37734754 | 2.586239268 | 2041 | ASR | 22.30831857 | 32.4463765 |
|  | 27.05649371 | 2.600720186 | 2042 | ASR | 21.95908214 | 32.15390527 |
|  | 26.73989 | 2.613160745 | 2043 | ASR | 21.61809494 | 31.86168506 |
|  | 26.42917883 | 2.626585392 | 2044 | ASR | 21.28107146 | 31.5772862 |
|  | 26.12653732 | 2.642504825 | 2045 | ASR | 20.94722786 | 31.30584678 |
|  | 25.83447366 | 2.65802268 | 2046 | ASR | 20.62474921 | 31.04419811 |
|  | 25.54359102 | 2.666992845 | 2047 | ASR | 20.31628505 | 30.770897 |
|  | 25.24862239 | 2.670409279 | 2048 | ASR | 20.0146202 | 30.48262457 |
|  | 24.95767729 | 2.678501886 | 2049 | ASR | 19.70781359 | 30.20754098 |
|  | 24.67237944 | 2.691683401 | 2050 | ASR | 19.39667998 | 29.94807891 |

**Table S4. Predictive analysis of LC until 2050 (number)**

| **Burden indicator** | **val** | **sd** | **Time** | **group** | **low_95** | **up_95** |
| --- | --- | --- | --- | --- | --- | --- |
| Incidence | 153931.7739 | 2589.102207 | 1990 | Number | 148857.1336 | 159006.4143 |
|  | 151248.844 | 2097.2952 | 1991 | Number | 147138.1454 | 155359.5426 |
|  | 153556.4905 | 2127.563208 | 1992 | Number | 149386.4666 | 157726.5144 |
|  | 156419.6475 | 2156.542896 | 1993 | Number | 152192.8234 | 160646.4716 |
|  | 158198.1912 | 2165.905163 | 1994 | Number | 153953.017 | 162443.3653 |
|  | 159637.3548 | 2184.931847 | 1995 | Number | 155354.8884 | 163919.8212 |
|  | 159447.5432 | 2181.606198 | 1996 | Number | 155171.595 | 163723.4913 |
|  | 158515.0937 | 2166.360491 | 1997 | Number | 154269.0271 | 162761.1603 |
|  | 157466.4445 | 2153.977939 | 1998 | Number | 153244.6477 | 161688.2412 |
|  | 155991.631 | 2138.878704 | 1999 | Number | 151799.4288 | 160183.8333 |
|  | 155257.5365 | 2137.845256 | 2000 | Number | 151067.3598 | 159447.7132 |
|  | 154080.0324 | 2127.657986 | 2001 | Number | 149909.8228 | 158250.2421 |
|  | 149114.5026 | 1706.164531 | 2002 | Number | 145770.4201 | 152458.585 |
|  | 149078.0994 | 1712.133208 | 2003 | Number | 145722.3183 | 152433.8805 |
|  | 145041.2724 | 1278.728418 | 2004 | Number | 142534.9647 | 147547.5801 |
|  | 146036.4256 | 1285.399292 | 2005 | Number | 143517.043 | 148555.8082 |
|  | 145754.2888 | 1280.488864 | 2006 | Number | 143244.5307 | 148264.047 |
|  | 146068.3874 | 1283.373205 | 2007 | Number | 143552.9759 | 148583.7988 |
|  | 147550.6024 | 1290.314054 | 2008 | Number | 145021.5868 | 150079.6179 |
|  | 147549.4127 | 1294.119182 | 2009 | Number | 145012.9391 | 150085.8863 |
|  | 148632.6815 | 1296.501199 | 2010 | Number | 146091.5392 | 151173.8239 |
|  | 149924.9005 | 1303.16552 | 2011 | Number | 147370.6961 | 152479.1049 |
|  | 151478.1942 | 1308.674096 | 2012 | Number | 148913.193 | 154043.1955 |
|  | 151573.0113 | 1313.387391 | 2013 | Number | 148998.772 | 154147.2506 |
|  | 151752.4126 | 1318.154488 | 2014 | Number | 149168.8298 | 154335.9954 |
|  | 153355.1512 | 1322.990059 | 2015 | Number | 150762.0907 | 155948.2117 |
|  | 154951.6914 | 1332.237397 | 2016 | Number | 152340.5061 | 157562.8767 |
|  | 155329.7559 | 1330.474572 | 2017 | Number | 152722.0257 | 157937.4861 |
|  | 156452.9411 | 1332.56236 | 2018 | Number | 153841.1189 | 159064.7634 |
|  | 157488.0315 | 1345.603728 | 2019 | Number | 154850.6482 | 160125.4148 |
|  | 158221.7488 | 1350.352219 | 2020 | Number | 155575.0585 | 160868.4392 |
|  | 158800.4419 | 1369.862515 | 2021 | Number | 156115.5114 | 161485.3724 |
|  | 190272.4799 | 7875.39598 | 2022 | Number | 174836.7038 | 205708.256 |
|  | 189204.958 | 9005.839644 | 2023 | Number | 171553.5123 | 206856.4037 |
|  | 188134.7909 | 9703.199586 | 2024 | Number | 169116.5197 | 207153.0621 |
|  | 187070.8323 | 10176.43368 | 2025 | Number | 167125.0223 | 207016.6423 |
|  | 186031.5177 | 10537.76428 | 2026 | Number | 165377.4997 | 206685.5357 |
|  | 184951.5124 | 10811.95523 | 2027 | Number | 163760.0801 | 206142.9446 |
|  | 183892.9768 | 11055.92427 | 2028 | Number | 162223.3653 | 205562.5884 |
|  | 182846.1524 | 11263.44778 | 2029 | Number | 160769.7948 | 204922.5101 |
|  | 181855.1062 | 11472.2897 | 2030 | Number | 159369.4184 | 204340.794 |
|  | 180910.5675 | 11663.95975 | 2031 | Number | 158049.2063 | 203771.9286 |
|  | 179988.2031 | 11855.99259 | 2032 | Number | 156750.4577 | 203225.9486 |
|  | 179085.7789 | 12036.50703 | 2033 | Number | 155494.2251 | 202677.3327 |
|  | 178220.7824 | 12215.54002 | 2034 | Number | 154278.324 | 202163.2409 |
|  | 177406.7239 | 12392.41236 | 2035 | Number | 153117.5957 | 201695.8521 |
|  | 176656.1874 | 12562.29414 | 2036 | Number | 152034.0909 | 201278.2839 |
|  | 175938.6001 | 12729.75264 | 2037 | Number | 150988.285 | 200888.9153 |
|  | 175227.0288 | 12880.87619 | 2038 | Number | 149980.5114 | 200473.5461 |
|  | 174535.6982 | 13031.17992 | 2039 | Number | 148994.5855 | 200076.8108 |
|  | 173861.1548 | 13167.44501 | 2040 | Number | 148052.9626 | 199669.347 |
|  | 173241.6008 | 13306.96555 | 2041 | Number | 147159.9484 | 199323.2533 |
|  | 172636.7773 | 13435.946 | 2042 | Number | 146302.3231 | 198971.2315 |
|  | 172029.467 | 13565.05276 | 2043 | Number | 145441.9636 | 198616.9704 |
|  | 171402.5952 | 13685.90139 | 2044 | Number | 144578.2284 | 198226.9619 |
|  | 170769.5626 | 13807.55806 | 2045 | Number | 143706.7488 | 197832.3764 |
|  | 170149.3219 | 13930.64997 | 2046 | Number | 142845.2479 | 197453.3958 |
|  | 169527.7098 | 14059.09146 | 2047 | Number | 141971.8906 | 197083.5291 |
|  | 168877.663 | 14193.86393 | 2048 | Number | 141057.6897 | 196697.6363 |
|  | 168188.2859 | 14331.8761 | 2049 | Number | 140097.8088 | 196278.7631 |
|  | 167468.4777 | 14478.34872 | 2050 | Number | 139090.9142 | 195846.0412 |
| Prevalence | 1194876.925 | 1093.104262 | 1990 | Number | 1192734.44 | 1197019.409 |
|  | 1213892.314 | 1101.767814 | 1991 | Number | 1211732.849 | 1216051.779 |
|  | 1270999.271 | 1127.386036 | 1992 | Number | 1268789.595 | 1273208.948 |
|  | 1233208.679 | 1110.499294 | 1993 | Number | 1231032.1 | 1235385.258 |
|  | 1244081.911 | 1115.3842 | 1994 | Number | 1241895.758 | 1246268.064 |
|  | 1304243.02 | 1142.034598 | 1995 | Number | 1302004.632 | 1306481.407 |
|  | 1206273.541 | 1098.30485 | 1996 | Number | 1204120.864 | 1208426.219 |
|  | 1202589.941 | 1096.62662 | 1997 | Number | 1200440.553 | 1204739.329 |
|  | 1198707.769 | 1094.855137 | 1998 | Number | 1196561.853 | 1200853.685 |
|  | 1189815.709 | 1090.78674 | 1999 | Number | 1187677.767 | 1191953.651 |
|  | 1251753.272 | 1118.817802 | 2000 | Number | 1249560.389 | 1253946.155 |
|  | 1243127.688 | 1114.956363 | 2001 | Number | 1240942.373 | 1245313.002 |
|  | 1236830.796 | 1112.128949 | 2002 | Number | 1234651.023 | 1239010.568 |
|  | 1236461.068 | 1111.962711 | 2003 | Number | 1234281.621 | 1238640.515 |
|  | 1237579.125 | 1112.465338 | 2004 | Number | 1235398.693 | 1239759.557 |
|  | 1250254.837 | 1118.147951 | 2005 | Number | 1248063.267 | 1252446.407 |
|  | 1255796.533 | 1120.62328 | 2006 | Number | 1253600.111 | 1257992.955 |
|  | 1261696.705 | 1123.252736 | 2007 | Number | 1259495.13 | 1263898.28 |
|  | 1119307.634 | 1057.973364 | 2008 | Number | 1117234.006 | 1121381.262 |
|  | 1121277.365 | 1058.903852 | 2009 | Number | 1119201.913 | 1123352.816 |
|  | 1134717.36 | 1065.231132 | 2010 | Number | 1132629.507 | 1136805.213 |
|  | 1146952.188 | 1070.958539 | 2011 | Number | 1144853.109 | 1149051.267 |
|  | 1152557.409 | 1073.572267 | 2012 | Number | 1150453.207 | 1154661.61 |
|  | 1191235.129 | 1091.437187 | 2013 | Number | 1189095.912 | 1193374.346 |
|  | 1192805.298 | 1092.156263 | 2014 | Number | 1190664.671 | 1194945.924 |
|  | 1208129.67 | 1099.149523 | 2015 | Number | 1205975.337 | 1210284.003 |
|  | 1220095.457 | 1104.579314 | 2016 | Number | 1217930.482 | 1222260.433 |
|  | 1096742.479 | 1047.254736 | 2017 | Number | 1094689.859 | 1098795.098 |
|  | 1104988.438 | 1051.184305 | 2018 | Number | 1102928.117 | 1107048.76 |
|  | 1056302.945 | 1027.765999 | 2019 | Number | 1054288.523 | 1058317.366 |
|  | 1058809.282 | 1028.984589 | 2020 | Number | 1056792.472 | 1060826.091 |
|  | 1067455.105 | 1033.177191 | 2021 | Number | 1065430.078 | 1069480.132 |
|  | 456930.5822 | 675.9664082 | 2022 | Number | 455605.6881 | 458255.4764 |
|  | 448485.4396 | 669.6905572 | 2023 | Number | 447172.8461 | 449798.033 |
|  | 371030.7308 | 929.7680782 | 2024 | Number | 369208.3854 | 372853.0763 |
|  | 350003.695 | 865.5496175 | 2025 | Number | 348307.2177 | 351700.1722 |
|  | 1842335.289 | 1480.287308 | 2026 | Number | 1839433.926 | 1845236.652 |
|  | 4361432.491 | 2165.160998 | 2027 | Number | 4357188.775 | 4365676.206 |
|  | 5054424.368 | 2999.15389 | 2028 | Number | 5048546.026 | 5060302.709 |
|  | 5491167.655 | 2969.040182 | 2029 | Number | 5485348.336 | 5496986.974 |
|  | 4936006.558 | 2725.037354 | 2030 | Number | 4930665.485 | 4941347.631 |
|  | 3377719.671 | 2242.996637 | 2031 | Number | 3373323.398 | 3382115.945 |
|  | 2703013.46 | 211793.3443 | 2032 | Number | 2287898.506 | 3118128.415 |
|  | 2115099.044 | 345391.5162 | 2033 | Number | 1438131.672 | 2792066.416 |
|  | 2075681.009 | 369247.6399 | 2034 | Number | 1351955.635 | 2799406.384 |
|  | 1563340.606 | 422478.6887 | 2035 | Number | 735282.3761 | 2391398.836 |
|  | 1502999.22 | 432132.8495 | 2036 | Number | 656018.8354 | 2349979.605 |
|  | 1277023.78 | 455488.2597 | 2037 | Number | 384266.7908 | 2169780.769 |
|  | 1266851.776 | 463643.2239 | 2038 | Number | 358111.0576 | 2175592.495 |
|  | 1269847.84 | 471576.4612 | 2039 | Number | 345557.9758 | 2194137.704 |
|  | 1273858.977 | 479920.6996 | 2040 | Number | 333214.4055 | 2214503.548 |
|  | 1277038.09 | 488087.9978 | 2041 | Number | 320385.614 | 2233690.565 |
|  | 1281352.976 | 496159.3755 | 2042 | Number | 308880.5997 | 2253825.352 |
|  | 1286136.815 | 504374.3365 | 2043 | Number | 297563.115 | 2274710.514 |
|  | 1291059.337 | 512294.3534 | 2044 | Number | 286962.4047 | 2295156.27 |
|  | 1295416.622 | 519557.0451 | 2045 | Number | 277084.8137 | 2313748.43 |
|  | 1295092.911 | 524957.1083 | 2046 | Number | 266176.9791 | 2324008.844 |
|  | 1297739.683 | 530927.9405 | 2047 | Number | 257120.9195 | 2338358.446 |
|  | 1299494.741 | 535289.1822 | 2048 | Number | 250327.9438 | 2348661.538 |
|  | 1301917.052 | 539688.7735 | 2049 | Number | 244127.0556 | 2359707.048 |
|  | 1300968.695 | 540469.4801 | 2050 | Number | 241648.5141 | 2360288.876 |
| Deaths | 118899.684 | 2042.723208 | 1990 | Number | 114895.9465 | 122903.4215 |
|  | 119972.6104 | 2012.446113 | 1991 | Number | 116028.216 | 123917.0047 |
|  | 121313.5879 | 2032.926947 | 1992 | Number | 117329.0511 | 125298.1247 |
|  | 122954.8456 | 2059.967687 | 1993 | Number | 118917.309 | 126992.3823 |
|  | 123745.0372 | 2075.13333 | 1994 | Number | 119677.7758 | 127812.2985 |
|  | 123692.8665 | 2076.351443 | 1995 | Number | 119623.2176 | 127762.5153 |
|  | 122496.2721 | 2058.751278 | 1996 | Number | 118461.1196 | 126531.4246 |
|  | 121258.7878 | 2040.02287 | 1997 | Number | 117260.343 | 125257.2326 |
|  | 120051.9656 | 2021.716181 | 1998 | Number | 116089.4019 | 124014.5293 |
|  | 118640.285 | 1999.307542 | 1999 | Number | 114721.6422 | 122558.9278 |
|  | 117898.2913 | 1988.214658 | 2000 | Number | 114001.3906 | 121795.192 |
|  | 116770.1186 | 1970.343453 | 2001 | Number | 112908.2455 | 120631.9918 |
|  | 115581.2159 | 1950.21822 | 2002 | Number | 111758.7882 | 119403.6436 |
|  | 114795.9566 | 1937.162695 | 2003 | Number | 110999.1177 | 118592.7954 |
|  | 113524.8215 | 1916.116044 | 2004 | Number | 109769.234 | 117280.4089 |
|  | 112790.9054 | 1903.65808 | 2005 | Number | 109059.7356 | 116522.0753 |
|  | 111141.006 | 1875.753597 | 2006 | Number | 107464.529 | 114817.4831 |
|  | 110592.586 | 1864.931572 | 2007 | Number | 106937.3201 | 114247.8519 |
|  | 111065.8626 | 1870.820887 | 2008 | Number | 107399.0537 | 114732.6715 |
|  | 110455.8888 | 1859.465909 | 2009 | Number | 106811.3356 | 114100.442 |
|  | 110798.066 | 1864.096954 | 2010 | Number | 107144.4359 | 114451.696 |
|  | 110859.3135 | 1863.026577 | 2011 | Number | 107207.7814 | 114510.8456 |
|  | 110840.3768 | 1859.233064 | 2012 | Number | 107196.28 | 114484.4736 |
|  | 110377.5405 | 1848.511157 | 2013 | Number | 106754.4586 | 114000.6224 |
|  | 110101.2177 | 1841.224149 | 2014 | Number | 106492.4183 | 113710.017 |
|  | 110443.4781 | 1844.943636 | 2015 | Number | 106827.3886 | 114059.5677 |
|  | 111030.5684 | 1851.843075 | 2016 | Number | 107400.956 | 114660.1808 |
|  | 110912.7961 | 1847.339592 | 2017 | Number | 107292.0106 | 114533.5817 |
|  | 111295.6576 | 1850.702337 | 2018 | Number | 107668.2811 | 114923.0342 |
|  | 111540.1905 | 1850.338848 | 2019 | Number | 107913.5264 | 115166.8547 |
|  | 111311.0143 | 1843.928131 | 2020 | Number | 107696.9152 | 114925.1134 |
|  | 110824.6569 | 1865.736632 | 2021 | Number | 107167.8131 | 114481.5007 |
|  | 109913.5337 | 3260.917845 | 2022 | Number | 103522.1347 | 116304.9326 |
|  | 108758.1288 | 3736.336317 | 2023 | Number | 101434.9096 | 116081.348 |
|  | 107593.5586 | 4019.900623 | 2024 | Number | 99714.55338 | 115472.5638 |
|  | 106428.0014 | 4213.260067 | 2025 | Number | 98170.01167 | 114685.9911 |
|  | 105263.3774 | 4366.178147 | 2026 | Number | 96705.66826 | 113821.0866 |
|  | 104081.1009 | 4488.941031 | 2027 | Number | 95282.77643 | 112879.4253 |
|  | 102913.7615 | 4604.309237 | 2028 | Number | 93889.31539 | 111938.2076 |
|  | 101756.4685 | 4706.993732 | 2029 | Number | 92530.76078 | 110982.1762 |
|  | 100631.1584 | 4812.140522 | 2030 | Number | 91199.36298 | 110062.9538 |
|  | 99520.55406 | 4910.463026 | 2031 | Number | 89896.04653 | 109145.0616 |
|  | 98418.29783 | 5009.699055 | 2032 | Number | 88599.28768 | 108237.308 |
|  | 97326.44855 | 5103.793561 | 2033 | Number | 87323.01317 | 107329.8839 |
|  | 96255.44633 | 5196.210898 | 2034 | Number | 86070.87297 | 106440.0197 |
|  | 95212.57102 | 5286.425119 | 2035 | Number | 84851.17778 | 105573.9643 |
|  | 94194.75198 | 5372.485458 | 2036 | Number | 83664.68049 | 104724.8235 |
|  | 93191.36171 | 5456.673193 | 2037 | Number | 82496.28225 | 103886.4412 |
|  | 92196.52616 | 5534.05071 | 2038 | Number | 81349.78677 | 103043.2655 |
|  | 91219.04338 | 5610.062197 | 2039 | Number | 80223.32147 | 102214.7653 |
|  | 90257.48479 | 5680.354195 | 2040 | Number | 79123.99057 | 101390.979 |
|  | 89319.45194 | 5750.607389 | 2041 | Number | 78048.26146 | 100590.6424 |
|  | 88387.97762 | 5816.658177 | 2042 | Number | 76987.32759 | 99788.62764 |
|  | 87463.08378 | 5882.29295 | 2043 | Number | 75933.7896 | 98992.37797 |
|  | 86542.83674 | 5945.26763 | 2044 | Number | 74890.11218 | 98195.56129 |
|  | 85634.99585 | 6008.853249 | 2045 | Number | 73857.64348 | 97412.34822 |
|  | 84741.98669 | 6073.197941 | 2046 | Number | 72838.51873 | 96645.45466 |
|  | 83856.68181 | 6139.616686 | 2047 | Number | 71823.03311 | 95890.33052 |
|  | 82972.57189 | 6208.440274 | 2048 | Number | 70804.02895 | 95141.11482 |
|  | 82089.65395 | 6279.432091 | 2049 | Number | 69781.96705 | 94397.34084 |
|  | 81213.45408 | 6354.614826 | 2050 | Number | 68758.40902 | 93668.49914 |
| DALYs | 3953596.91 | 2237.856251 | 1990 | Number | 3949210.712 | 3957983.109 |
|  | 3983308.739 | 2243.529106 | 1991 | Number | 3978911.422 | 3987706.056 |
|  | 4043489.697 | 2257.851171 | 1992 | Number | 4039064.309 | 4047915.085 |
|  | 3759219.434 | 2195.19589 | 1993 | Number | 3754916.851 | 3763522.018 |
|  | 3770398.045 | 2199.210563 | 1994 | Number | 3766087.593 | 3774708.498 |
|  | 3756028.92 | 2192.838997 | 1995 | Number | 3751730.956 | 3760326.885 |
|  | 3700816.883 | 2027.497985 | 1996 | Number | 3696842.987 | 3704790.779 |
|  | 3649640.3 | 2017.530579 | 1997 | Number | 3645685.94 | 3653594.66 |
|  | 3749442.99 | 2045.529704 | 1998 | Number | 3745433.751 | 3753452.228 |
|  | 3716656.127 | 2040.447011 | 1999 | Number | 3712656.851 | 3720655.403 |
|  | 3681703.498 | 2034.122141 | 2000 | Number | 3677716.619 | 3685690.378 |
|  | 3577704.161 | 2008.202388 | 2001 | Number | 3573768.084 | 3581640.238 |
|  | 3530517.932 | 1997.244727 | 2002 | Number | 3526603.332 | 3534432.531 |
|  | 3805814.226 | 2066.382894 | 2003 | Number | 3801764.115 | 3809864.336 |
|  | 3756744.581 | 2056.256059 | 2004 | Number | 3752714.319 | 3760774.843 |
|  | 3726131.743 | 2050.653041 | 2005 | Number | 3722112.463 | 3730151.023 |
|  | 3498063.327 | 1995.668608 | 2006 | Number | 3494151.816 | 3501974.837 |
|  | 3565813.687 | 2016.680218 | 2007 | Number | 3561860.994 | 3569766.38 |
|  | 3570597.454 | 2023.241296 | 2008 | Number | 3566631.901 | 3574563.006 |
|  | 3532341.019 | 2017.346805 | 2009 | Number | 3528387.019 | 3536295.019 |
|  | 3534020.185 | 2023.685307 | 2010 | Number | 3530053.762 | 3537986.609 |
|  | 3526414.233 | 2025.078175 | 2011 | Number | 3522445.079 | 3530383.386 |
|  | 3364750.765 | 1988.546561 | 2012 | Number | 3360853.214 | 3368648.316 |
|  | 3335072.468 | 1985.683141 | 2013 | Number | 3331180.529 | 3338964.407 |
|  | 3301377.498 | 1979.446575 | 2014 | Number | 3297497.783 | 3305257.213 |
|  | 3342572.427 | 1994.698305 | 2015 | Number | 3338662.818 | 3346482.035 |
|  | 3219643.026 | 1971.374707 | 2016 | Number | 3215779.132 | 3223506.921 |
|  | 3208675.044 | 1974.639084 | 2017 | Number | 3204804.751 | 3212545.336 |
|  | 3211533.153 | 1979.097695 | 2018 | Number | 3207654.121 | 3215412.184 |
|  | 3353423.002 | 2023.747614 | 2019 | Number | 3349456.457 | 3357389.547 |
|  | 3337479.626 | 1826.877015 | 2020 | Number | 3333898.947 | 3341060.305 |
|  | 3191074.595 | 1786.357914 | 2021 | Number | 3187573.334 | 3194575.857 |
|  | 2935667.605 | 127225.373 | 2022 | Number | 2686305.874 | 3185029.336 |
|  | 2910411.013 | 145027.7038 | 2023 | Number | 2626156.713 | 3194665.312 |
|  | 2884984.029 | 158576.2496 | 2024 | Number | 2574174.58 | 3195793.479 |
|  | 2859726.501 | 169492.778 | 2025 | Number | 2527520.656 | 3191932.345 |
|  | 2834958.006 | 178324.3948 | 2026 | Number | 2485442.192 | 3184473.82 |
|  | 2810864.245 | 185816.7371 | 2027 | Number | 2446663.44 | 3175065.05 |
|  | 2787254.325 | 192123.0766 | 2028 | Number | 2410693.095 | 3163815.556 |
|  | 2764040.177 | 197652.2249 | 2029 | Number | 2376641.816 | 3151438.538 |
|  | 2741098.013 | 202452.6206 | 2030 | Number | 2344290.876 | 3137905.149 |
|  | 2719052.026 | 206804.7796 | 2031 | Number | 2313714.658 | 3124389.394 |
|  | 2697728.52 | 210722.4939 | 2032 | Number | 2284712.432 | 3110744.608 |
|  | 2676925.263 | 214381.5423 | 2033 | Number | 2256737.44 | 3097113.086 |
|  | 2656435.363 | 217810.3789 | 2034 | Number | 2229527.02 | 3083343.705 |
|  | 2636320.234 | 221099.3886 | 2035 | Number | 2202965.432 | 3069675.036 |
|  | 2617065.116 | 224238.7274 | 2036 | Number | 2177557.211 | 3056573.022 |
|  | 2598398.274 | 227226.2304 | 2037 | Number | 2153034.862 | 3043761.686 |
|  | 2579895.605 | 230080.4967 | 2038 | Number | 2128937.831 | 3030853.378 |
|  | 2561499.963 | 232933.7002 | 2039 | Number | 2104949.91 | 3018050.015 |
|  | 2543276.864 | 235789.1584 | 2040 | Number | 2081130.114 | 3005423.614 |
|  | 2525617.313 | 238591.2501 | 2041 | Number | 2057978.462 | 2993256.163 |
|  | 2507995.674 | 241078.3816 | 2042 | Number | 2035482.046 | 2980509.302 |
|  | 2489973.648 | 243338.2562 | 2043 | Number | 2013030.666 | 2966916.63 |
|  | 2471710.176 | 245648.6011 | 2044 | Number | 1990238.918 | 2953181.434 |
|  | 2453427.798 | 248150.9077 | 2045 | Number | 1967052.019 | 2939803.577 |
|  | 2435381.707 | 250573.1467 | 2046 | Number | 1944258.34 | 2926505.075 |
|  | 2416710.894 | 252332.2966 | 2047 | Number | 1922139.593 | 2911282.196 |
|  | 2396935.063 | 253515.4947 | 2048 | Number | 1900044.694 | 2893825.433 |
|  | 2376835.883 | 255090.8723 | 2049 | Number | 1876857.773 | 2876813.992 |
|  | 2356583.822 | 257100.8866 | 2050 | Number | 1852666.084 | 2860501.559 |

**Table S5.The proportional distribution of the causes of LC within the global scope and five SDI regions.**

| **measure_name** | **location_name** | **rei_name** | **metric_name** | **val** | **upper** | **lower** |
| --- | --- | --- | --- | --- | --- | --- |
| Deaths | Global | High alcohol use | Percent | 12.32319354 | 17.66721057 | 6.812820182 |
| Deaths | Global | Smoking | Percent | 66.23023164 | 70.91433521 | 60.63230249 |
| Deaths | Global | Occupational exposure to sulfuric acid | Percent | 3.011393216 | 5.398674885 | 1.23580125 |
| Deaths | Global | Occupational risks | Percent | 5.957434807 | 8.658960199 | 3.747515428 |
| Deaths | Global | Occupational exposure to asbestos | Percent | 2.995000159 | 4.492367935 | 1.700752338 |
| Deaths | Global | Behavioral risks | Percent | 69.58563453 | 73.85732231 | 64.42890313 |
| Deaths | Global | Tobacco | Percent | 66.23023164 | 70.91433521 | 60.63230249 |
| Deaths | Global | Occupational carcinogens | Percent | 5.957434807 | 8.658960199 | 3.747515428 |
| Deaths | Global | Environmental/occupational risks | Percent | 5.957434807 | 8.658960199 | 3.747515428 |
| Deaths | High SDI | Smoking | Percent | 72.43772177 | 78.05186693 | 65.92253969 |
| Deaths | Middle SDI | Smoking | Percent | 64.55463964 | 69.41015023 | 58.81572781 |
| Deaths | Low SDI | Smoking | Percent | 48.57884496 | 53.54447753 | 42.7473706 |
| Deaths | High-middle SDI | Smoking | Percent | 75.76598307 | 79.904909 | 70.44395941 |
| Deaths | Low-middle SDI | Smoking | Percent | 63.6316296 | 68.40710531 | 58.14850292 |
| Deaths | High SDI | Occupational exposure to asbestos | Percent | 8.591713156 | 12.48748464 | 5.016770659 |
| Deaths | Middle SDI | Occupational exposure to asbestos | Percent | 1.605244509 | 2.466795217 | 0.886651786 |
| Deaths | Low SDI | Occupational exposure to asbestos | Percent | 1.178947037 | 2.069467886 | 0.563226117 |
| Deaths | High-middle SDI | Occupational exposure to asbestos | Percent | 3.431336524 | 5.15865888 | 1.92976828 |
| Deaths | High SDI | Behavioral risks | Percent | 77.50087217 | 82.47479796 | 71.57538547 |
| Deaths | Low-middle SDI | Occupational exposure to asbestos | Percent | 1.362242104 | 2.206451731 | 0.736264183 |
| Deaths | High-middle SDI | Behavioral risks | Percent | 79.25043068 | 83.02768386 | 74.47609732 |
| Deaths | High-middle SDI | High alcohol use | Percent | 17.37418538 | 24.46469631 | 9.467248359 |
| Deaths | High SDI | High alcohol use | Percent | 19.84328497 | 27.8788207 | 10.77992285 |
| Deaths | Middle SDI | Behavioral risks | Percent | 67.703433 | 72.3699136 | 62.56750861 |
| Deaths | Low-middle SDI | High alcohol use | Percent | 6.744190422 | 10.06562995 | 3.345388307 |
| Deaths | Low SDI | Behavioral risks | Percent | 51.90824556 | 56.60567422 | 46.52631964 |
| Deaths | Low-middle SDI | Behavioral risks | Percent | 65.93967627 | 70.45019964 | 60.56568523 |
| Deaths | Middle SDI | High alcohol use | Percent | 11.32370468 | 16.48192493 | 6.085107 |
| Deaths | Low SDI | High alcohol use | Percent | 6.497244219 | 9.904671538 | 3.088765602 |
| Deaths | High-middle SDI | Occupational risks | Percent | 6.050765209 | 8.657995463 | 3.727700373 |
| Deaths | High-middle SDI | Tobacco | Percent | 75.76598307 | 79.904909 | 70.44395941 |
| Deaths | Low SDI | Occupational risks | Percent | 3.743806298 | 6.150067369 | 2.152823766 |
| Deaths | Middle SDI | Occupational risks | Percent | 4.870686223 | 7.684108919 | 2.900380056 |
| Deaths | High SDI | Occupational risks | Percent | 10.74304126 | 14.80988109 | 6.515151784 |
| Deaths | Low-middle SDI | Occupational risks | Percent | 4.424505852 | 7.187741516 | 2.637064226 |
| Deaths | High-middle SDI | Occupational exposure to sulfuric acid | Percent | 2.671066272 | 4.846390375 | 1.08162035 |
| Deaths | Low-middle SDI | Tobacco | Percent | 63.6316296 | 68.40710531 | 58.14850292 |
| Deaths | High SDI | Tobacco | Percent | 72.43772177 | 78.05186693 | 65.92253969 |
| Deaths | Low-middle SDI | Occupational exposure to sulfuric acid | Percent | 3.090588741 | 5.693988175 | 1.285217144 |
| Deaths | Middle SDI | Tobacco | Percent | 64.55463964 | 69.41015023 | 58.81572781 |
| Deaths | Middle SDI | Occupational exposure to sulfuric acid | Percent | 3.298621884 | 6.132199821 | 1.375437438 |
| Deaths | Low SDI | Tobacco | Percent | 48.57884496 | 53.54447753 | 42.7473706 |
| Deaths | High SDI | Occupational exposure to sulfuric acid | Percent | 2.282494186 | 4.664797504 | 0.623118537 |
| Deaths | Low SDI | Occupational exposure to sulfuric acid | Percent | 2.586954669 | 4.844265652 | 1.061473133 |
| Deaths | High-middle SDI | Occupational carcinogens | Percent | 6.050765209 | 8.657995463 | 3.727700373 |
| Deaths | Low-middle SDI | Occupational carcinogens | Percent | 4.424505852 | 7.187741516 | 2.637064226 |
| Deaths | High SDI | Occupational carcinogens | Percent | 10.74304126 | 14.80988109 | 6.515151784 |
| Deaths | Middle SDI | Occupational carcinogens | Percent | 4.870686223 | 7.684108919 | 2.900380056 |
| Deaths | Low SDI | Occupational carcinogens | Percent | 3.743806298 | 6.150067369 | 2.152823766 |
| Deaths | High SDI | Environmental/occupational risks | Percent | 10.74304126 | 14.80988109 | 6.515151784 |
| Deaths | Low SDI | Environmental/occupational risks | Percent | 3.743806298 | 6.150067369 | 2.152823766 |
| Deaths | Middle SDI | Environmental/occupational risks | Percent | 4.870686223 | 7.684108919 | 2.900380056 |
| Deaths | High-middle SDI | Environmental/occupational risks | Percent | 6.050765209 | 8.657995463 | 3.727700373 |
| Deaths | Low-middle SDI | Environmental/occupational risks | Percent | 4.424505852 | 7.187741516 | 2.637064226 |
| DALYs | Global | Smoking | Percent | 65.28306759 | 69.66559385 | 59.99258611 |
| DALYs | Global | High alcohol use | Percent | 12.93083177 | 18.28163539 | 7.171178048 |
| DALYs | Global | Occupational exposure to asbestos | Percent | 2.170300921 | 3.285797911 | 1.220268866 |
| DALYs | Global | Tobacco | Percent | 65.28306759 | 69.66559385 | 59.99258611 |
| DALYs | Global | Behavioral risks | Percent | 68.87409862 | 73.01464829 | 63.72754592 |
| DALYs | Global | Occupational exposure to sulfuric acid | Percent | 3.606351026 | 6.432198653 | 1.480888386 |
| DALYs | Global | Occupational risks | Percent | 5.729014676 | 8.854718692 | 3.495053443 |
| DALYs | Global | Occupational carcinogens | Percent | 5.729014676 | 8.854718692 | 3.495053443 |
| DALYs | Global | Environmental/occupational risks | Percent | 5.729014676 | 8.854718692 | 3.495053443 |
| DALYs | High SDI | High alcohol use | Percent | 20.85901464 | 28.98286863 | 11.51092649 |
| DALYs | High-middle SDI | Tobacco | Percent | 76.75187525 | 80.60113311 | 71.67777913 |
| DALYs | High SDI | Tobacco | Percent | 73.77942224 | 78.91476245 | 67.71221582 |
| DALYs | Low-middle SDI | Tobacco | Percent | 61.88847055 | 66.57331602 | 56.52718053 |
| DALYs | High SDI | Occupational exposure to sulfuric acid | Percent | 2.719884668 | 5.544982113 | 0.74567297 |
| DALYs | High-middle SDI | High alcohol use | Percent | 18.5795346 | 25.95406021 | 10.40691656 |
| DALYs | Low SDI | Tobacco | Percent | 47.32500508 | 52.08921362 | 41.65467213 |
| DALYs | Low-middle SDI | High alcohol use | Percent | 7.358394833 | 10.93234278 | 3.700168761 |
| DALYs | Low SDI | High alcohol use | Percent | 7.019053737 | 10.57656903 | 3.350809781 |
| DALYs | Middle SDI | Tobacco | Percent | 63.51939291 | 68.3271446 | 57.92224446 |
| DALYs | High-middle SDI | Occupational exposure to sulfuric acid | Percent | 3.208090095 | 5.798636655 | 1.298445957 |
| DALYs | Middle SDI | High alcohol use | Percent | 12.37407214 | 17.75665603 | 6.666896774 |
| DALYs | High SDI | Smoking | Percent | 73.77942224 | 78.91476245 | 67.71221582 |
| DALYs | Low-middle SDI | Occupational exposure to sulfuric acid | Percent | 3.718944629 | 6.867337295 | 1.545770018 |
| DALYs | Low SDI | Occupational exposure to sulfuric acid | Percent | 3.169206144 | 5.944569457 | 1.303993429 |
| DALYs | Low-middle SDI | Smoking | Percent | 61.88847055 | 66.57331602 | 56.52718053 |
| DALYs | Low SDI | Smoking | Percent | 47.32500508 | 52.08921362 | 41.65467213 |
| DALYs | Middle SDI | Occupational exposure to sulfuric acid | Percent | 4.015071224 | 7.426182633 | 1.67917842 |
| DALYs | High-middle SDI | Smoking | Percent | 76.75187525 | 80.60113311 | 71.67777913 |
| DALYs | High SDI | Occupational risks | Percent | 9.050029616 | 12.66366927 | 5.374396347 |
| DALYs | Low-middle SDI | Occupational risks | Percent | 4.735042031 | 7.960384477 | 2.606830611 |
| DALYs | Low SDI | Occupational risks | Percent | 4.07963444 | 6.945833713 | 2.277176991 |
| DALYs | Middle SDI | Smoking | Percent | 63.51939291 | 68.3271446 | 57.92224446 |
| DALYs | Middle SDI | Occupational risks | Percent | 5.207392922 | 8.63237791 | 2.873271457 |
| DALYs | High-middle SDI | Occupational risks | Percent | 5.723319634 | 8.502630598 | 3.499446884 |
| DALYs | High SDI | Occupational exposure to asbestos | Percent | 6.454946532 | 9.516335096 | 3.695847339 |
| DALYs | Low-middle SDI | Occupational exposure to asbestos | Percent | 1.044888784 | 1.700298086 | 0.563390914 |
| DALYs | Low SDI | Occupational exposure to asbestos | Percent | 0.933045688 | 1.661442356 | 0.430814521 |
| DALYs | High-middle SDI | Occupational exposure to asbestos | Percent | 2.566461087 | 3.87785643 | 1.434393105 |
| DALYs | High-middle SDI | Occupational carcinogens | Percent | 5.723319634 | 8.502630598 | 3.499446884 |
| DALYs | Middle SDI | Occupational exposure to asbestos | Percent | 1.227929628 | 1.918606107 | 0.67436156 |
| DALYs | Low-middle SDI | Occupational carcinogens | Percent | 4.735042031 | 7.960384477 | 2.606830611 |
| DALYs | High SDI | Occupational carcinogens | Percent | 9.050029616 | 12.66366927 | 5.374396347 |
| DALYs | Middle SDI | Occupational carcinogens | Percent | 5.207392922 | 8.63237791 | 2.873271457 |
| DALYs | High SDI | Behavioral risks | Percent | 78.90085855 | 83.38269404 | 73.48623169 |
| DALYs | Low-middle SDI | Behavioral risks | Percent | 64.53180839 | 68.9895547 | 59.37161802 |
| DALYs | Low SDI | Occupational carcinogens | Percent | 4.07963444 | 6.945833713 | 2.277176991 |
| DALYs | Low SDI | Behavioral risks | Percent | 50.97535474 | 55.53057428 | 45.76629212 |
| DALYs | High SDI | Environmental/occupational risks | Percent | 9.050029616 | 12.66366927 | 5.374396347 |
| DALYs | High-middle SDI | Behavioral risks | Percent | 80.3524437 | 83.79966795 | 76.09320021 |
| DALYs | Middle SDI | Behavioral risks | Percent | 67.15450805 | 71.63069162 | 62.01900231 |
| DALYs | High-middle SDI | Environmental/occupational risks | Percent | 5.723319634 | 8.502630598 | 3.499446884 |
| DALYs | Low-middle SDI | Environmental/occupational risks | Percent | 4.735042031 | 7.960384477 | 2.606830611 |
| DALYs | Middle SDI | Environmental/occupational risks | Percent | 5.207392922 | 8.63237791 | 2.873271457 |
| DALYs | Low SDI | Environmental/occupational risks | Percent | 4.07963444 | 6.945833713 | 2.277176991 |

**Table S6.The proportional distribution of cause of LC among different age groups on a global scale.**

| **measure_name** | **age_name** | **rei_name** | **metric_name** | **val** | **upper** | **lower** |
| --- | --- | --- | --- | --- | --- | --- |
| Deaths | 75-79 years | High alcohol use | Percent | 10.66242439 | 15.60614062 | 5.540458372 |
| Deaths | 80-84 years | High alcohol use | Percent | 8.846763773 | 13.24817214 | 4.454615054 |
| Deaths | 85-89 years | High alcohol use | Percent | 9.577677186 | 14.20154965 | 4.666533983 |
| Deaths | 90-94 years | High alcohol use | Percent | 9.944058621 | 14.90732632 | 4.793960023 |
| Deaths | 75-79 years | Smoking | Percent | 66.84605267 | 72.40780216 | 60.58406196 |
| Deaths | 80-84 years | Smoking | Percent | 63.28896911 | 69.19017534 | 56.47323392 |
| Deaths | 85-89 years | Smoking | Percent | 62.08946227 | 68.71946712 | 55.04328082 |
| Deaths | 90-94 years | Smoking | Percent | 58.61505364 | 66.16075645 | 51.02577095 |
| Deaths | 95+ years | Smoking | Percent | 53.1668958 | 62.19487238 | 45.5063752 |
| Deaths | 95+ years | High alcohol use | Percent | 9.682688139 | 14.56279451 | 4.566220659 |
| Deaths | 20-24 years | Occupational risks | Percent | 0.000384384 | 0.001242778 | 3.46976E-05 |
| Deaths | 25-29 years | Occupational risks | Percent | 2.717619904 | 5.042530141 | 1.116525155 |
| Deaths | 30-34 years | Occupational risks | Percent | 2.927203524 | 5.356613447 | 1.219460627 |
| Deaths | 35-39 years | Occupational risks | Percent | 3.689056474 | 6.659278583 | 1.568503076 |
| Deaths | 40-44 years | Occupational risks | Percent | 5.02182462 | 8.906758436 | 2.241862907 |
| Deaths | 45-49 years | Occupational risks | Percent | 5.265545936 | 9.161842164 | 2.521407899 |
| Deaths | 50-54 years | Occupational risks | Percent | 5.962642365 | 10.15890058 | 2.994497991 |
| Deaths | 55-59 years | Occupational risks | Percent | 5.662495436 | 9.21393287 | 3.059842517 |
| Deaths | 60-64 years | Occupational risks | Percent | 5.819285202 | 9.309658717 | 3.294065165 |
| Deaths | 65-69 years | Occupational risks | Percent | 5.467981629 | 8.255771408 | 3.356044249 |
| Deaths | 55-59 years | Occupational exposure to sulfuric acid | Percent | 4.492847733 | 8.010255708 | 1.840740955 |
| Deaths | 60-64 years | Occupational exposure to sulfuric acid | Percent | 4.280160513 | 7.626492546 | 1.749584743 |
| Deaths | 65-69 years | Occupational exposure to sulfuric acid | Percent | 3.296811641 | 5.929079408 | 1.340659634 |
| Deaths | 70-74 years | Occupational exposure to sulfuric acid | Percent | 2.226979656 | 4.006767971 | 0.898477185 |
| Deaths | 75-79 years | Occupational exposure to sulfuric acid | Percent | 1.17085907 | 2.09669381 | 0.468624191 |
| Deaths | 80-84 years | Occupational exposure to sulfuric acid | Percent | 0.506675109 | 0.946370561 | 0.197905896 |
| Deaths | 85-89 years | Occupational exposure to sulfuric acid | Percent | 0.161634607 | 0.303439713 | 0.062214281 |
| Deaths | 90-94 years | Occupational exposure to sulfuric acid | Percent | 0.035442348 | 0.068196799 | 0.013060312 |
| Deaths | 95+ years | Occupational exposure to sulfuric acid | Percent | 0.007344649 | 0.014449247 | 0.002594215 |
| Deaths | 30-34 years | Tobacco | Percent | 29.48770771 | 33.26108024 | 25.03940032 |
| Deaths | 35-39 years | Tobacco | Percent | 40.58462353 | 44.66572643 | 35.89460131 |
| Deaths | 40-44 years | Tobacco | Percent | 49.80567728 | 54.23674351 | 44.9153587 |
| Deaths | 45-49 years | Tobacco | Percent | 57.58931423 | 62.00809604 | 52.19292016 |
| Deaths | 50-54 years | Tobacco | Percent | 64.03353426 | 68.54319243 | 58.78918016 |
| Deaths | 55-59 years | Tobacco | Percent | 69.14986995 | 73.57547098 | 63.60485689 |
| Deaths | 60-64 years | Tobacco | Percent | 70.42616715 | 75.12256247 | 65.0536025 |
| Deaths | 70-74 years | Occupational risks | Percent | 5.685715839 | 8.001593471 | 3.565609422 |
| Deaths | 75-79 years | Occupational risks | Percent | 6.188729181 | 8.723965359 | 3.880684244 |
| Deaths | 80-84 years | Occupational risks | Percent | 6.862133746 | 9.892442243 | 4.078135138 |
| Deaths | 85-89 years | Occupational risks | Percent | 7.973428482 | 11.41694353 | 4.697722889 |
| Deaths | 90-94 years | Occupational risks | Percent | 8.979361632 | 12.76150427 | 5.172927899 |
| Deaths | 20-24 years | High alcohol use | Percent | 6.941474274 | 10.89787943 | 3.605637991 |
| Deaths | 25-29 years | High alcohol use | Percent | 8.267175094 | 12.65110877 | 4.302816201 |
| Deaths | 30-34 years | High alcohol use | Percent | 10.51305491 | 15.21149913 | 5.451117488 |
| Deaths | 35-39 years | High alcohol use | Percent | 12.13309525 | 17.4581828 | 6.362023396 |
| Deaths | 40-44 years | High alcohol use | Percent | 13.24177882 | 18.84416774 | 7.083816129 |
| Deaths | 45-49 years | High alcohol use | Percent | 14.01872004 | 19.58434387 | 7.625232826 |
| Deaths | 50-54 years | High alcohol use | Percent | 14.07663965 | 19.90647302 | 7.546711238 |
| Deaths | 55-59 years | High alcohol use | Percent | 14.1312388 | 19.95722792 | 7.691633332 |
| Deaths | 60-64 years | High alcohol use | Percent | 13.86756679 | 19.80641742 | 7.577865494 |
| Deaths | 65-69 years | High alcohol use | Percent | 12.71275082 | 18.30829344 | 6.841146022 |
| Deaths | 70-74 years | High alcohol use | Percent | 11.88138249 | 17.06737628 | 6.263748419 |
| Deaths | 60-64 years | Occupational exposure to asbestos | Percent | 1.598812989 | 2.514667785 | 0.864657366 |
| Deaths | 65-69 years | Occupational exposure to asbestos | Percent | 2.233055673 | 3.432900127 | 1.238745576 |
| Deaths | 70-74 years | Occupational exposure to asbestos | Percent | 3.531590394 | 5.287841772 | 1.977770981 |
| Deaths | 75-79 years | Occupational exposure to asbestos | Percent | 5.073598638 | 7.579038726 | 2.917247968 |
| Deaths | 80-84 years | Occupational exposure to asbestos | Percent | 6.393761386 | 9.280789649 | 3.706902299 |
| Deaths | 85-89 years | Occupational exposure to asbestos | Percent | 7.825812653 | 11.27687695 | 4.563026502 |
| Deaths | 90-94 years | Occupational exposure to asbestos | Percent | 8.947146013 | 12.74037135 | 5.144328002 |
| Deaths | 95+ years | Occupational exposure to asbestos | Percent | 8.828643525 | 12.50266825 | 5.210607506 |
| Deaths | 20-24 years | Occupational exposure to sulfuric acid | Percent | 0 | 0 | 0 |
| Deaths | 25-29 years | Occupational exposure to sulfuric acid | Percent | 2.714459888 | 5.039266545 | 1.113009176 |
| Deaths | 30-34 years | Occupational exposure to sulfuric acid | Percent | 2.920036759 | 5.344384321 | 1.209393342 |
| Deaths | 35-39 years | Occupational exposure to sulfuric acid | Percent | 3.64936969 | 6.609399649 | 1.510860788 |
| Deaths | 40-44 years | Occupational exposure to sulfuric acid | Percent | 4.903085529 | 8.758715143 | 2.03076522 |
| Deaths | 45-49 years | Occupational exposure to sulfuric acid | Percent | 4.950451989 | 8.794318973 | 2.041232479 |
| Deaths | 50-54 years | Occupational exposure to sulfuric acid | Percent | 5.281973611 | 9.361389028 | 2.199768271 |
| Deaths | 40-44 years | Behavioral risks | Percent | 55.3900995 | 59.91513087 | 50.19824475 |
| Deaths | 45-49 years | Behavioral risks | Percent | 62.42367521 | 66.47956467 | 57.31715712 |
| Deaths | 50-54 years | Behavioral risks | Percent | 68.07981964 | 72.59416634 | 62.671853 |
| Deaths | 55-59 years | Behavioral risks | Percent | 72.57017682 | 76.61612804 | 67.87013514 |
| Deaths | 60-64 years | Behavioral risks | Percent | 73.6614573 | 77.86558974 | 68.87984695 |
| Deaths | 65-69 years | Behavioral risks | Percent | 73.53370359 | 77.75488222 | 67.93807236 |
| Deaths | 70-74 years | Behavioral risks | Percent | 72.70717366 | 77.28470572 | 67.35996914 |
| Deaths | 75-79 years | Behavioral risks | Percent | 69.77391493 | 74.8182999 | 63.47336825 |
| Deaths | 80-84 years | Behavioral risks | Percent | 66.11986033 | 71.69196269 | 59.53931445 |
| Deaths | 85-89 years | Behavioral risks | Percent | 65.35139324 | 71.49467544 | 58.53747587 |
| Deaths | 90-94 years | Behavioral risks | Percent | 62.4440434 | 69.18613116 | 55.07908662 |
| Deaths | 95+ years | Behavioral risks | Percent | 57.56610561 | 65.80841432 | 50.30533783 |
| Deaths | 65-69 years | Tobacco | Percent | 70.62147738 | 75.17722425 | 65.0470639 |
| Deaths | 70-74 years | Tobacco | Percent | 69.83295703 | 74.71299321 | 64.0858808 |
| Deaths | 75-79 years | Tobacco | Percent | 66.84605267 | 72.40780216 | 60.58406196 |
| Deaths | 80-84 years | Tobacco | Percent | 63.28896911 | 69.19017534 | 56.47323392 |
| Deaths | 85-89 years | Tobacco | Percent | 62.08946227 | 68.71946712 | 55.04328082 |
| Deaths | 90-94 years | Tobacco | Percent | 58.61505364 | 66.16075645 | 51.02577095 |
| Deaths | 95+ years | Occupational risks | Percent | 8.835393638 | 12.5111597 | 5.217043074 |
| Deaths | 20-24 years | Occupational carcinogens | Percent | 0.000384384 | 0.001242778 | 3.46976E-05 |
| Deaths | 25-29 years | Occupational carcinogens | Percent | 2.717619904 | 5.042530141 | 1.116525155 |
| Deaths | 30-34 years | Occupational carcinogens | Percent | 2.927203524 | 5.356613447 | 1.219460627 |
| Deaths | 35-39 years | Occupational carcinogens | Percent | 3.689056474 | 6.659278583 | 1.568503076 |
| Deaths | 40-44 years | Occupational carcinogens | Percent | 5.02182462 | 8.906758436 | 2.241862907 |
| Deaths | 45-49 years | Occupational carcinogens | Percent | 5.265545936 | 9.161842164 | 2.521407899 |
| Deaths | 50-54 years | Occupational carcinogens | Percent | 5.962642365 | 10.15890058 | 2.994497991 |
| Deaths | 55-59 years | Occupational carcinogens | Percent | 5.662495436 | 9.21393287 | 3.059842517 |
| Deaths | 60-64 years | Occupational carcinogens | Percent | 5.819285202 | 9.309658717 | 3.294065165 |
| Deaths | 20-24 years | Environmental/occupational risks | Percent | 0.000384384 | 0.001242778 | 3.46976E-05 |
| Deaths | 25-29 years | Environmental/occupational risks | Percent | 2.717619904 | 5.042530141 | 1.116525155 |
| Deaths | 30-34 years | Environmental/occupational risks | Percent | 2.927203524 | 5.356613447 | 1.219460627 |
| Deaths | 35-39 years | Environmental/occupational risks | Percent | 3.689056474 | 6.659278583 | 1.568503076 |
| Deaths | 40-44 years | Environmental/occupational risks | Percent | 5.02182462 | 8.906758436 | 2.241862907 |
| Deaths | 95+ years | Tobacco | Percent | 53.1668958 | 62.19487238 | 45.5063752 |
| Deaths | 30-34 years | Smoking | Percent | 29.48770771 | 33.26108024 | 25.03940032 |
| Deaths | 35-39 years | Smoking | Percent | 40.58462353 | 44.66572643 | 35.89460131 |
| Deaths | 40-44 years | Smoking | Percent | 49.80567728 | 54.23674351 | 44.9153587 |
| Deaths | 45-49 years | Smoking | Percent | 57.58931423 | 62.00809604 | 52.19292016 |
| Deaths | 50-54 years | Smoking | Percent | 64.03353426 | 68.54319243 | 58.78918016 |
| Deaths | 55-59 years | Smoking | Percent | 69.14986995 | 73.57547098 | 63.60485689 |
| Deaths | 60-64 years | Smoking | Percent | 70.42616715 | 75.12256247 | 65.0536025 |
| Deaths | 65-69 years | Smoking | Percent | 70.62147738 | 75.17722425 | 65.0470639 |
| Deaths | 70-74 years | Smoking | Percent | 69.83295703 | 74.71299321 | 64.0858808 |
| Deaths | 65-69 years | Occupational carcinogens | Percent | 5.467981629 | 8.255771408 | 3.356044249 |
| Deaths | 70-74 years | Occupational carcinogens | Percent | 5.685715839 | 8.001593471 | 3.565609422 |
| Deaths | 75-79 years | Occupational carcinogens | Percent | 6.188729181 | 8.723965359 | 3.880684244 |
| Deaths | 80-84 years | Occupational carcinogens | Percent | 6.862133746 | 9.892442243 | 4.078135138 |
| Deaths | 85-89 years | Occupational carcinogens | Percent | 7.973428482 | 11.41694353 | 4.697722889 |
| Deaths | 90-94 years | Occupational carcinogens | Percent | 8.979361632 | 12.76150427 | 5.172927899 |
| Deaths | 95+ years | Occupational carcinogens | Percent | 8.835393638 | 12.5111597 | 5.217043074 |
| Deaths | 20-24 years | Occupational exposure to asbestos | Percent | 0.000384384 | 0.001242778 | 3.46976E-05 |
| Deaths | 25-29 years | Occupational exposure to asbestos | Percent | 0.003263391 | 0.007508603 | 0.001100477 |
| Deaths | 30-34 years | Occupational exposure to asbestos | Percent | 0.007395739 | 0.01680432 | 0.002482456 |
| Deaths | 35-39 years | Occupational exposure to asbestos | Percent | 0.041228288 | 0.077035033 | 0.01908581 |
| Deaths | 40-44 years | Occupational exposure to asbestos | Percent | 0.124831693 | 0.215871685 | 0.060156639 |
| Deaths | 45-49 years | Occupational exposure to asbestos | Percent | 0.331489515 | 0.544859768 | 0.17222144 |
| Deaths | 50-54 years | Occupational exposure to asbestos | Percent | 0.716422352 | 1.140071565 | 0.386989311 |
| Deaths | 55-59 years | Occupational exposure to asbestos | Percent | 1.221428139 | 1.896833842 | 0.64266373 |
| Deaths | 45-49 years | Environmental/occupational risks | Percent | 5.265545936 | 9.161842164 | 2.521407899 |
| Deaths | 50-54 years | Environmental/occupational risks | Percent | 5.962642365 | 10.15890058 | 2.994497991 |
| Deaths | 55-59 years | Environmental/occupational risks | Percent | 5.662495436 | 9.21393287 | 3.059842517 |
| Deaths | 60-64 years | Environmental/occupational risks | Percent | 5.819285202 | 9.309658717 | 3.294065165 |
| Deaths | 65-69 years | Environmental/occupational risks | Percent | 5.467981629 | 8.255771408 | 3.356044249 |
| Deaths | 70-74 years | Environmental/occupational risks | Percent | 5.685715839 | 8.001593471 | 3.565609422 |
| Deaths | 75-79 years | Environmental/occupational risks | Percent | 6.188729181 | 8.723965359 | 3.880684244 |
| Deaths | 80-84 years | Environmental/occupational risks | Percent | 6.862133746 | 9.892442243 | 4.078135138 |
| Deaths | 85-89 years | Environmental/occupational risks | Percent | 7.973428482 | 11.41694353 | 4.697722889 |
| Deaths | 90-94 years | Environmental/occupational risks | Percent | 8.979361632 | 12.76150427 | 5.172927899 |
| Deaths | 95+ years | Environmental/occupational risks | Percent | 8.835393638 | 12.5111597 | 5.217043074 |
| Deaths | 20-24 years | Behavioral risks | Percent | 6.941474274 | 10.89787943 | 3.605637991 |
| Deaths | 25-29 years | Behavioral risks | Percent | 8.267175094 | 12.65110877 | 4.302816201 |
| Deaths | 30-34 years | Behavioral risks | Percent | 35.71691598 | 40.21042281 | 30.48924865 |
| Deaths | 35-39 years | Behavioral risks | Percent | 46.52893523 | 50.91776573 | 41.39303493 |
| DALYs | 80-84 years | Smoking | Percent | 63.29096854 | 69.22921366 | 56.4549348 |
| DALYs | 85-89 years | Smoking | Percent | 62.04315856 | 68.69645957 | 55.01704171 |
| DALYs | 90-94 years | Smoking | Percent | 58.53569399 | 66.11872203 | 50.94932008 |
| DALYs | 95+ years | Smoking | Percent | 53.15914327 | 62.22216599 | 45.49732451 |
| DALYs | 20-24 years | High alcohol use | Percent | 6.95546575 | 10.91513188 | 3.613298228 |
| DALYs | 25-29 years | High alcohol use | Percent | 8.28428347 | 12.67350751 | 4.313037653 |
| DALYs | 30-34 years | High alcohol use | Percent | 10.5381644 | 15.24033654 | 5.46875684 |
| DALYs | 35-39 years | High alcohol use | Percent | 12.16322238 | 17.48491935 | 6.381761587 |
| DALYs | 40-44 years | High alcohol use | Percent | 13.27708093 | 18.87823587 | 7.103820086 |
| DALYs | 45-49 years | High alcohol use | Percent | 14.06420078 | 19.63566984 | 7.65271963 |
| DALYs | 50-54 years | High alcohol use | Percent | 14.13478966 | 19.99416728 | 7.591644297 |
| DALYs | 55-59 years | High alcohol use | Percent | 14.20017385 | 20.03433256 | 7.739775798 |
| DALYs | 80-84 years | High alcohol use | Percent | 8.895236929 | 13.32694038 | 4.475992563 |
| DALYs | 85-89 years | High alcohol use | Percent | 9.628905061 | 14.30061634 | 4.691610126 |
| DALYs | 90-94 years | High alcohol use | Percent | 9.985085623 | 14.97809539 | 4.815348288 |
| DALYs | 95+ years | High alcohol use | Percent | 9.694170703 | 14.59741544 | 4.567274859 |
| DALYs | 20-24 years | Occupational risks | Percent | 0.000384649 | 0.001247692 | 3.4598E-05 |
| DALYs | 25-29 years | Occupational risks | Percent | 2.717587573 | 5.041038142 | 1.116446572 |
| DALYs | 30-34 years | Occupational risks | Percent | 2.927232531 | 5.355066132 | 1.21936806 |
| DALYs | 35-39 years | Occupational risks | Percent | 3.688326001 | 6.656264838 | 1.568608435 |
| DALYs | 40-44 years | Occupational risks | Percent | 5.018414835 | 8.897552396 | 2.239861916 |
| DALYs | 45-49 years | Occupational risks | Percent | 5.26411282 | 9.150611167 | 2.519093929 |
| DALYs | 50-54 years | Occupational risks | Percent | 5.960094599 | 10.14409841 | 2.992844937 |
| DALYs | 60-64 years | High alcohol use | Percent | 13.93984216 | 19.8801675 | 7.623983576 |
| DALYs | 65-69 years | High alcohol use | Percent | 12.78975229 | 18.44355286 | 6.880289145 |
| DALYs | 70-74 years | High alcohol use | Percent | 11.95045397 | 17.16815898 | 6.297966498 |
| DALYs | 75-79 years | High alcohol use | Percent | 10.72970482 | 15.67781831 | 5.570147704 |
| DALYs | 75-79 years | Occupational exposure to asbestos | Percent | 5.155697102 | 7.679629283 | 2.966143723 |
| DALYs | 80-84 years | Occupational exposure to asbestos | Percent | 6.475629677 | 9.405386792 | 3.759615438 |
| DALYs | 85-89 years | Occupational exposure to asbestos | Percent | 7.94115921 | 11.42040422 | 4.635288585 |
| DALYs | 90-94 years | Occupational exposure to asbestos | Percent | 9.04454299 | 12.88043838 | 5.20624014 |
| DALYs | 95+ years | Occupational exposure to asbestos | Percent | 8.828612554 | 12.50351547 | 5.213164087 |
| DALYs | 30-34 years | Tobacco | Percent | 29.55585952 | 33.32202796 | 25.11311697 |
| DALYs | 35-39 years | Tobacco | Percent | 40.66519007 | 44.76707915 | 35.98732114 |
| DALYs | 40-44 years | Tobacco | Percent | 49.88463909 | 54.32157901 | 45.02138784 |
| DALYs | 45-49 years | Tobacco | Percent | 57.67069362 | 62.07457015 | 52.27189943 |
| DALYs | 50-54 years | Tobacco | Percent | 64.13869635 | 68.65830327 | 58.89814336 |
| DALYs | 55-59 years | Tobacco | Percent | 69.24865086 | 73.67063215 | 63.69259329 |
| DALYs | 60-64 years | Tobacco | Percent | 70.51726726 | 75.20688663 | 65.14328915 |
| DALYs | 65-69 years | Tobacco | Percent | 70.71272378 | 75.23922722 | 65.13638365 |
| DALYs | 70-74 years | Tobacco | Percent | 69.89005763 | 74.76987484 | 64.08152238 |
| DALYs | 75-79 years | Tobacco | Percent | 66.90999388 | 72.45495487 | 60.61323804 |
| DALYs | 20-24 years | Occupational exposure to sulfuric acid | Percent | 0 | 0 | 0 |
| DALYs | 25-29 years | Occupational exposure to sulfuric acid | Percent | 2.714421273 | 5.037771835 | 1.112921084 |
| DALYs | 30-34 years | Occupational exposure to sulfuric acid | Percent | 2.920048813 | 5.342815063 | 1.209273752 |
| DALYs | 35-39 years | Occupational exposure to sulfuric acid | Percent | 3.648550501 | 6.606320248 | 1.510919453 |
| DALYs | 40-44 years | Occupational exposure to sulfuric acid | Percent | 4.899430903 | 8.749374184 | 2.028913944 |
| DALYs | 45-49 years | Occupational exposure to sulfuric acid | Percent | 4.948147061 | 8.788314982 | 2.040227467 |
| DALYs | 50-54 years | Occupational exposure to sulfuric acid | Percent | 5.276728834 | 9.351780637 | 2.197016945 |
| DALYs | 55-59 years | Occupational exposure to sulfuric acid | Percent | 4.491090936 | 8.01419542 | 1.839940624 |
| DALYs | 60-64 years | Occupational exposure to sulfuric acid | Percent | 4.276409854 | 7.608813185 | 1.749080808 |
| DALYs | 65-69 years | Occupational exposure to sulfuric acid | Percent | 3.296410902 | 5.926817983 | 1.338781572 |
| DALYs | 55-59 years | Behavioral risks | Percent | 72.67406555 | 76.71043301 | 67.98517886 |
| DALYs | 60-64 years | Behavioral risks | Percent | 73.76060551 | 77.96051917 | 68.96744059 |
| DALYs | 65-69 years | Behavioral risks | Percent | 73.63478627 | 77.84721678 | 68.04610365 |
| DALYs | 70-74 years | Behavioral risks | Percent | 72.77693299 | 77.34533633 | 67.4464825 |
| DALYs | 75-79 years | Behavioral risks | Percent | 69.85248166 | 74.88087451 | 63.51846366 |
| DALYs | 80-84 years | Behavioral risks | Percent | 66.13950107 | 71.72830901 | 59.55093695 |
| DALYs | 85-89 years | Behavioral risks | Percent | 65.32775352 | 71.48953225 | 58.49098108 |
| DALYs | 90-94 years | Behavioral risks | Percent | 62.3873527 | 69.13602687 | 55.03907277 |
| DALYs | 55-59 years | Occupational risks | Percent | 5.669403842 | 9.221208522 | 3.062311257 |
| DALYs | 60-64 years | Occupational risks | Percent | 5.835433897 | 9.323960416 | 3.306583299 |
| DALYs | 65-69 years | Occupational risks | Percent | 5.50514596 | 8.282370547 | 3.375598628 |
| DALYs | 70-74 years | Occupational risks | Percent | 5.74641393 | 8.08612585 | 3.620169332 |
| DALYs | 75-79 years | Occupational risks | Percent | 6.270731699 | 8.820939546 | 3.935003645 |
| DALYs | 70-74 years | Occupational exposure to sulfuric acid | Percent | 2.229879637 | 4.012751408 | 0.90099372 |
| DALYs | 75-79 years | Occupational exposure to sulfuric acid | Percent | 1.171656613 | 2.09766522 | 0.468720442 |
| DALYs | 80-84 years | Occupational exposure to sulfuric acid | Percent | 0.50844305 | 0.952579361 | 0.198245308 |
| DALYs | 85-89 years | Occupational exposure to sulfuric acid | Percent | 0.162456839 | 0.304156517 | 0.062361757 |
| DALYs | 90-94 years | Occupational exposure to sulfuric acid | Percent | 0.035648872 | 0.068916155 | 0.013032219 |
| DALYs | 95+ years | Occupational exposure to sulfuric acid | Percent | 0.007276282 | 0.014317067 | 0.002569169 |
| DALYs | 95+ years | Behavioral risks | Percent | 57.56695022 | 65.81586839 | 50.31031266 |
| DALYs | 80-84 years | Occupational risks | Percent | 6.945127905 | 9.988342244 | 4.136267998 |
| DALYs | 85-89 years | Occupational risks | Percent | 8.089328585 | 11.55113845 | 4.773242938 |
| DALYs | 90-94 years | Occupational risks | Percent | 9.076915076 | 12.90162378 | 5.234815016 |
| DALYs | 95+ years | Occupational risks | Percent | 8.835300727 | 12.51192039 | 5.219543643 |
| DALYs | 20-24 years | Occupational carcinogens | Percent | 0.000384649 | 0.001247692 | 3.4598E-05 |
| DALYs | 25-29 years | Occupational carcinogens | Percent | 2.717587573 | 5.041038142 | 1.116446572 |
| DALYs | 30-34 years | Occupational carcinogens | Percent | 2.927232531 | 5.355066132 | 1.21936806 |
| DALYs | 35-39 years | Occupational carcinogens | Percent | 3.688326001 | 6.656264838 | 1.568608435 |
| DALYs | 40-44 years | Occupational carcinogens | Percent | 5.018414835 | 8.897552396 | 2.239861916 |
| DALYs | 45-49 years | Occupational carcinogens | Percent | 5.26411282 | 9.150611167 | 2.519093929 |
| DALYs | 80-84 years | Tobacco | Percent | 63.29096854 | 69.22921366 | 56.4549348 |
| DALYs | 85-89 years | Tobacco | Percent | 62.04315856 | 68.69645957 | 55.01704171 |
| DALYs | 90-94 years | Tobacco | Percent | 58.53569399 | 66.11872203 | 50.94932008 |
| DALYs | 95+ years | Tobacco | Percent | 53.15914327 | 62.22216599 | 45.49732451 |
| DALYs | 30-34 years | Smoking | Percent | 29.55585952 | 33.32202796 | 25.11311697 |
| DALYs | 35-39 years | Smoking | Percent | 40.66519007 | 44.76707915 | 35.98732114 |
| DALYs | 40-44 years | Smoking | Percent | 49.88463909 | 54.32157901 | 45.02138784 |
| DALYs | 45-49 years | Smoking | Percent | 57.67069362 | 62.07457015 | 52.27189943 |
| DALYs | 50-54 years | Smoking | Percent | 64.13869635 | 68.65830327 | 58.89814336 |
| DALYs | 55-59 years | Smoking | Percent | 69.24865086 | 73.67063215 | 63.69259329 |
| DALYs | 60-64 years | Smoking | Percent | 70.51726726 | 75.20688663 | 65.14328915 |
| DALYs | 65-69 years | Smoking | Percent | 70.71272378 | 75.23922722 | 65.13638365 |
| DALYs | 70-74 years | Smoking | Percent | 69.89005763 | 74.76987484 | 64.08152238 |
| DALYs | 75-79 years | Smoking | Percent | 66.90999388 | 72.45495487 | 60.61323804 |
| DALYs | 50-54 years | Occupational carcinogens | Percent | 5.960094599 | 10.14409841 | 2.992844937 |
| DALYs | 55-59 years | Occupational carcinogens | Percent | 5.669403842 | 9.221208522 | 3.062311257 |
| DALYs | 60-64 years | Occupational carcinogens | Percent | 5.835433897 | 9.323960416 | 3.306583299 |
| DALYs | 65-69 years | Occupational carcinogens | Percent | 5.50514596 | 8.282370547 | 3.375598628 |
| DALYs | 70-74 years | Occupational carcinogens | Percent | 5.74641393 | 8.08612585 | 3.620169332 |
| DALYs | 75-79 years | Occupational carcinogens | Percent | 6.270731699 | 8.820939546 | 3.935003645 |
| DALYs | 80-84 years | Occupational carcinogens | Percent | 6.945127905 | 9.988342244 | 4.136267998 |
| DALYs | 85-89 years | Occupational carcinogens | Percent | 8.089328585 | 11.55113845 | 4.773242938 |
| DALYs | 90-94 years | Occupational carcinogens | Percent | 9.076915076 | 12.90162378 | 5.234815016 |
| DALYs | 95+ years | Occupational carcinogens | Percent | 8.835300727 | 12.51192039 | 5.219543643 |
| DALYs | 20-24 years | Occupational exposure to asbestos | Percent | 0.000384649 | 0.001247692 | 3.4598E-05 |
| DALYs | 25-29 years | Occupational exposure to asbestos | Percent | 0.003269915 | 0.007520275 | 0.001101959 |
| DALYs | 30-34 years | Occupational exposure to asbestos | Percent | 0.007413318 | 0.016837196 | 0.002487731 |
| DALYs | 35-39 years | Occupational exposure to asbestos | Percent | 0.041320206 | 0.077112567 | 0.019145985 |
| DALYs | 40-44 years | Occupational exposure to asbestos | Percent | 0.125081515 | 0.216026056 | 0.060314894 |
| DALYs | 20-24 years | Environmental/occupational risks | Percent | 0.000384649 | 0.001247692 | 3.4598E-05 |
| DALYs | 25-29 years | Environmental/occupational risks | Percent | 2.717587573 | 5.041038142 | 1.116446572 |
| DALYs | 30-34 years | Environmental/occupational risks | Percent | 2.927232531 | 5.355066132 | 1.21936806 |
| DALYs | 35-39 years | Environmental/occupational risks | Percent | 3.688326001 | 6.656264838 | 1.568608435 |
| DALYs | 40-44 years | Environmental/occupational risks | Percent | 5.018414835 | 8.897552396 | 2.239861916 |
| DALYs | 45-49 years | Environmental/occupational risks | Percent | 5.26411282 | 9.150611167 | 2.519093929 |
| DALYs | 50-54 years | Environmental/occupational risks | Percent | 5.960094599 | 10.14409841 | 2.992844937 |
| DALYs | 55-59 years | Environmental/occupational risks | Percent | 5.669403842 | 9.221208522 | 3.062311257 |
| DALYs | 45-49 years | Occupational exposure to asbestos | Percent | 0.332385418 | 0.546248837 | 0.172835236 |
| DALYs | 50-54 years | Occupational exposure to asbestos | Percent | 0.719165128 | 1.143713603 | 0.388609564 |
| DALYs | 55-59 years | Occupational exposure to asbestos | Percent | 1.230361552 | 1.909471234 | 0.647293594 |
| DALYs | 60-64 years | Occupational exposure to asbestos | Percent | 1.61936047 | 2.539862014 | 0.876617787 |
| DALYs | 65-69 years | Occupational exposure to asbestos | Percent | 2.271567083 | 3.48971904 | 1.259844539 |
| DALYs | 70-74 years | Occupational exposure to asbestos | Percent | 3.590696494 | 5.352660571 | 2.011943719 |
| DALYs | 60-64 years | Environmental/occupational risks | Percent | 5.835433897 | 9.323960416 | 3.306583299 |
| DALYs | 65-69 years | Environmental/occupational risks | Percent | 5.50514596 | 8.282370547 | 3.375598628 |
| DALYs | 70-74 years | Environmental/occupational risks | Percent | 5.74641393 | 8.08612585 | 3.620169332 |
| DALYs | 75-79 years | Environmental/occupational risks | Percent | 6.270731699 | 8.820939546 | 3.935003645 |
| DALYs | 80-84 years | Environmental/occupational risks | Percent | 6.945127905 | 9.988342244 | 4.136267998 |
| DALYs | 85-89 years | Environmental/occupational risks | Percent | 8.089328585 | 11.55113845 | 4.773242938 |
| DALYs | 90-94 years | Environmental/occupational risks | Percent | 9.076915076 | 12.90162378 | 5.234815016 |
| DALYs | 95+ years | Environmental/occupational risks | Percent | 8.835300727 | 12.51192039 | 5.219543643 |
| DALYs | 20-24 years | Behavioral risks | Percent | 6.95546575 | 10.91513188 | 3.613298228 |
| DALYs | 25-29 years | Behavioral risks | Percent | 8.28428347 | 12.67350751 | 4.313037653 |
| DALYs | 30-34 years | Behavioral risks | Percent | 35.79138779 | 40.26238431 | 30.56501469 |
| DALYs | 35-39 years | Behavioral risks | Percent | 46.61174585 | 51.0294261 | 41.47602664 |
| DALYs | 40-44 years | Behavioral risks | Percent | 55.47161968 | 59.98699245 | 50.29035223 |
| DALYs | 45-49 years | Behavioral risks | Percent | 62.50843378 | 66.55643839 | 57.38669313 |
| DALYs | 50-54 years | Behavioral risks | Percent | 68.1878313 | 72.70146995 | 62.78805526 |

| 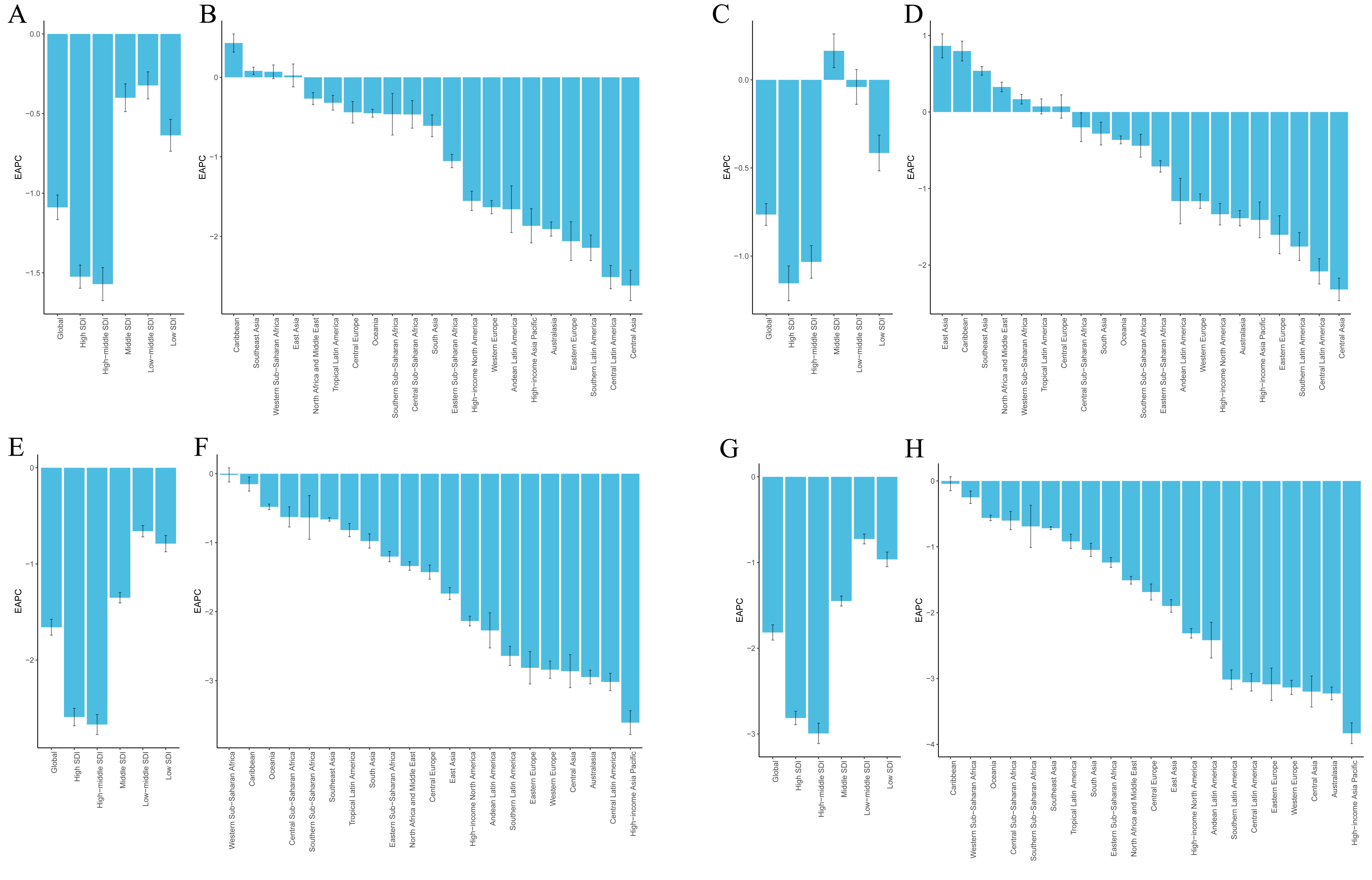 |
| --- |
| **Figure S1. Trends in EAPC across different SDI regions and 21 GBD regions from 1990 to 2021.** A.EAPC of incidence in the five SDI regions. B. EAPC of incidence in the 21 regions. C.EAPC of prevalence in the five SDI regions. D. EAPC of prevalence in the 21 regions. E. EAPC of deaths in the five SDI regions. F. EAPC of deaths in the 21 regions. G.EAPC of DALYs in the five SDI regions. H. EAPC of DALYs in the 21 regions. |

| 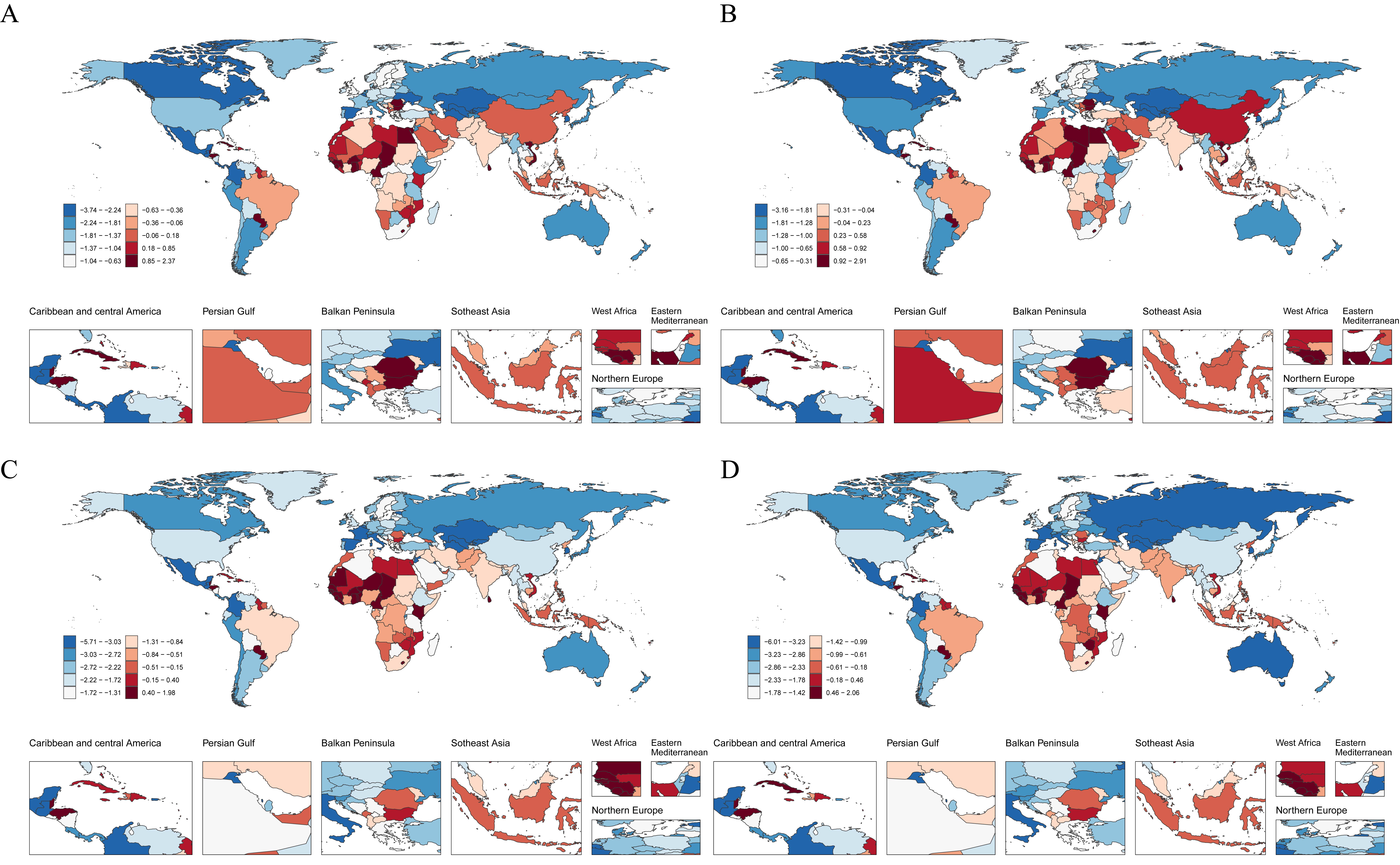 |
| --- |
| **Figure S2. The change trend of EAPCs in 204 countries and regions in 1990-2021.** (A) Age - standardized incidence. (B) Age - standardized prevalence . (C) Age - standardized deaths. (D) Age - standardized DALYs . |
| 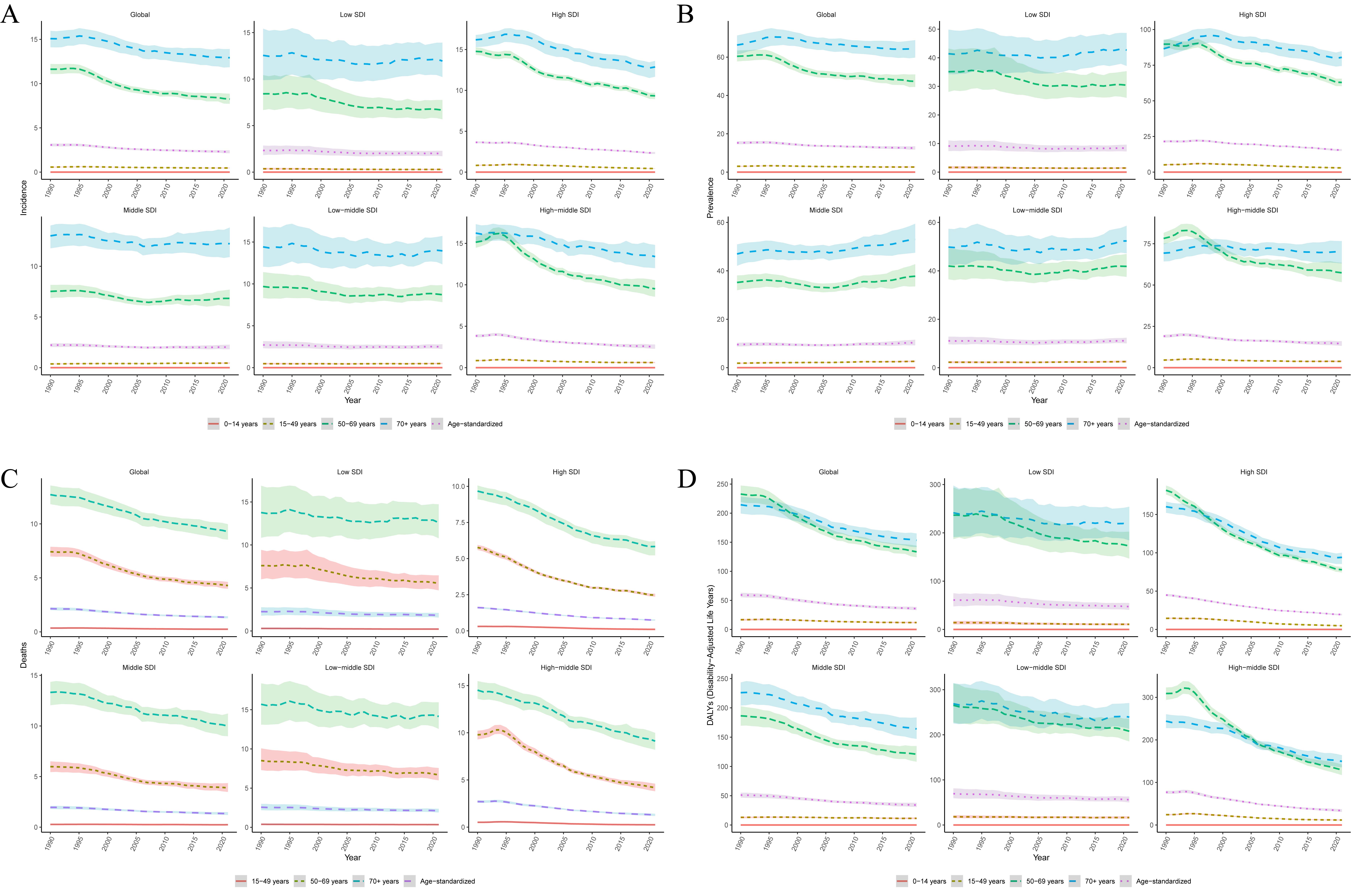 |
| **Figure S3. Analysis of the disease burden of LC by age - time from 1990 to 2021 .**(A) incidence. (B) prevalence . (C) deaths. (D) DALYs . |
| 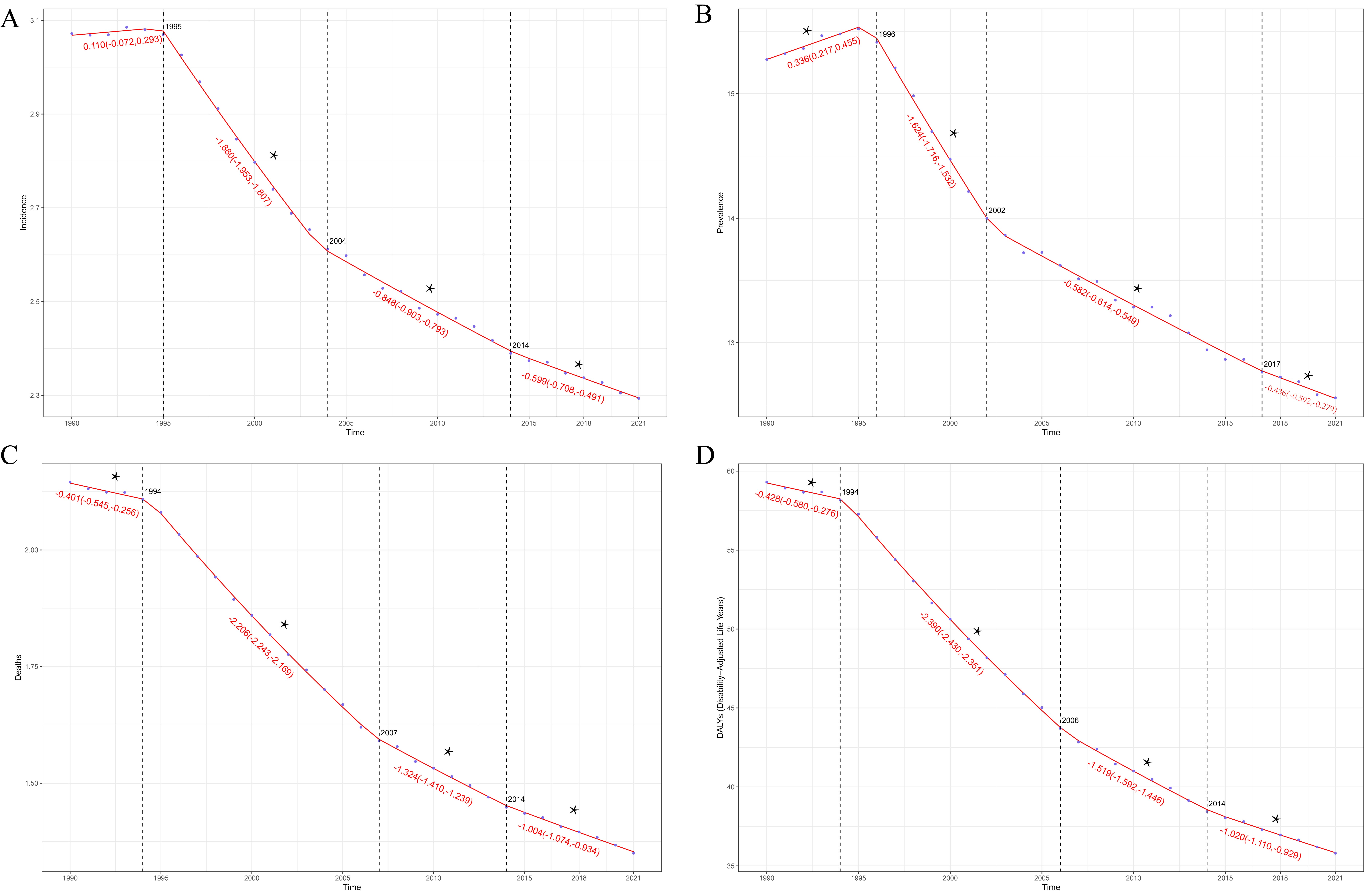 |
| **Figure S4. Joinpoint regression analysis results for LC.** (A) incidence. (B) prevalence . (C) deaths. (D) DALYs . |

| 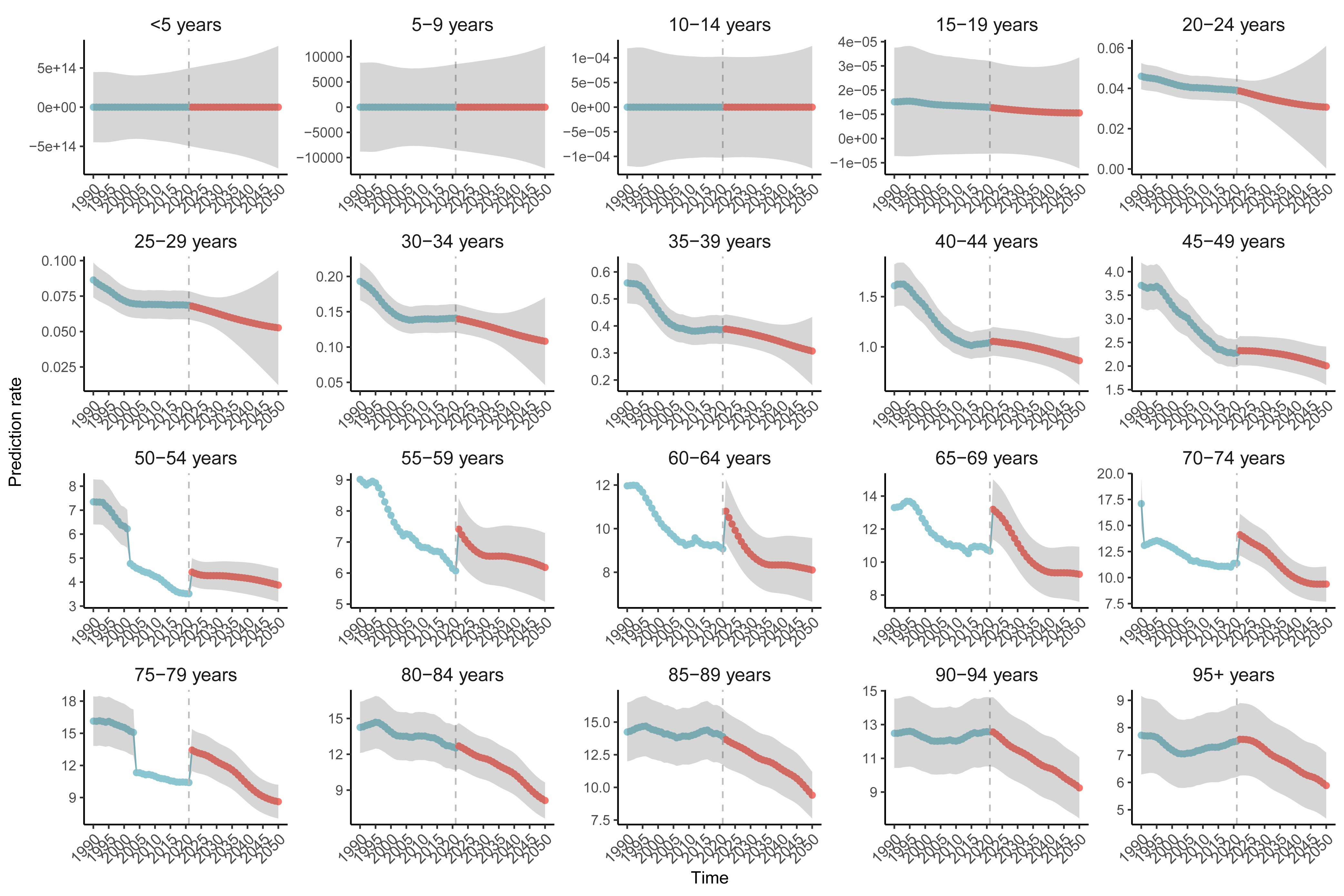 |
| --- |
| **Figure S5. Predicted LC incidence rates across different age groups** |

| 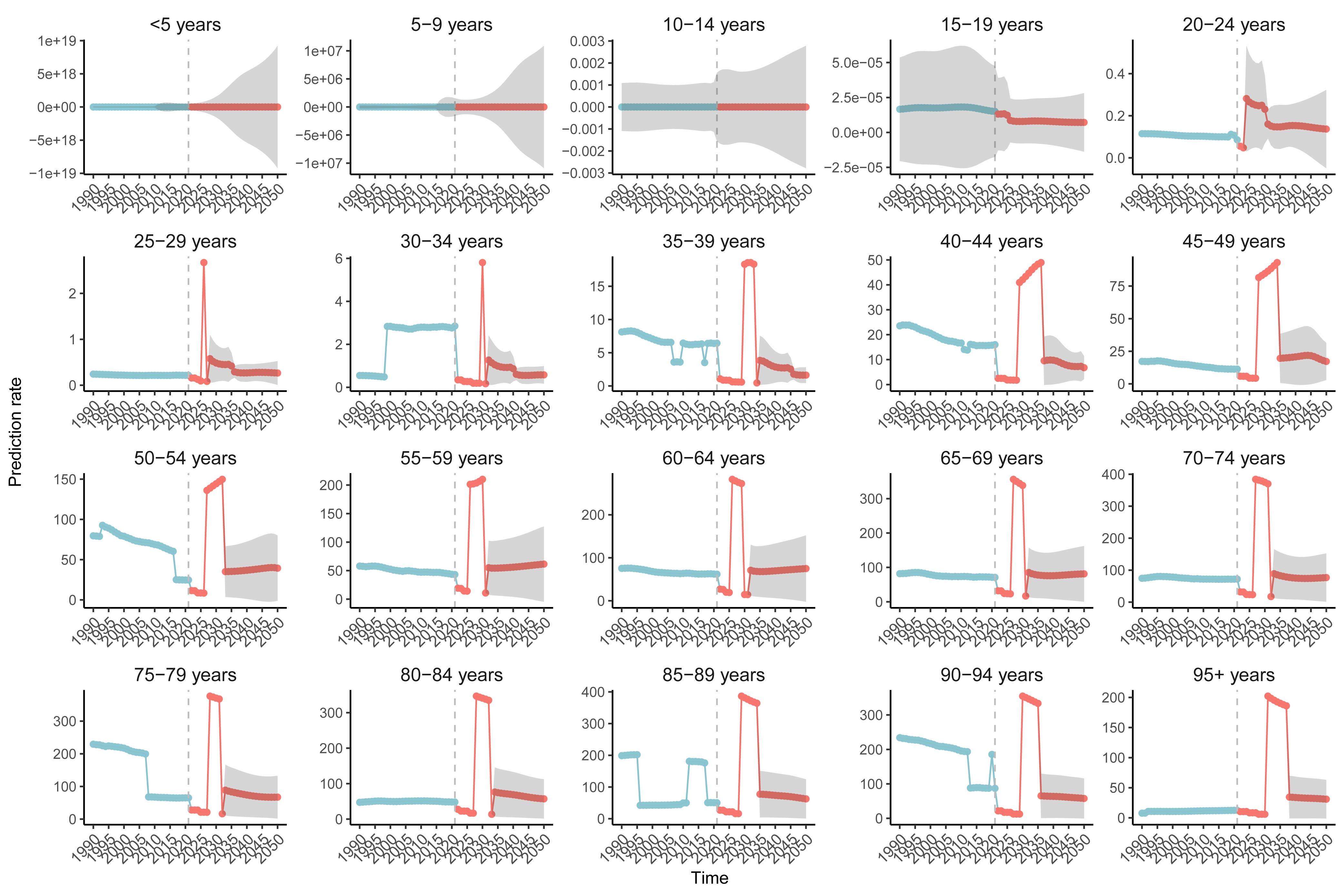 |
| --- |
| **Figure S6. Predicted LC prevalence rates across different age groups** |

| 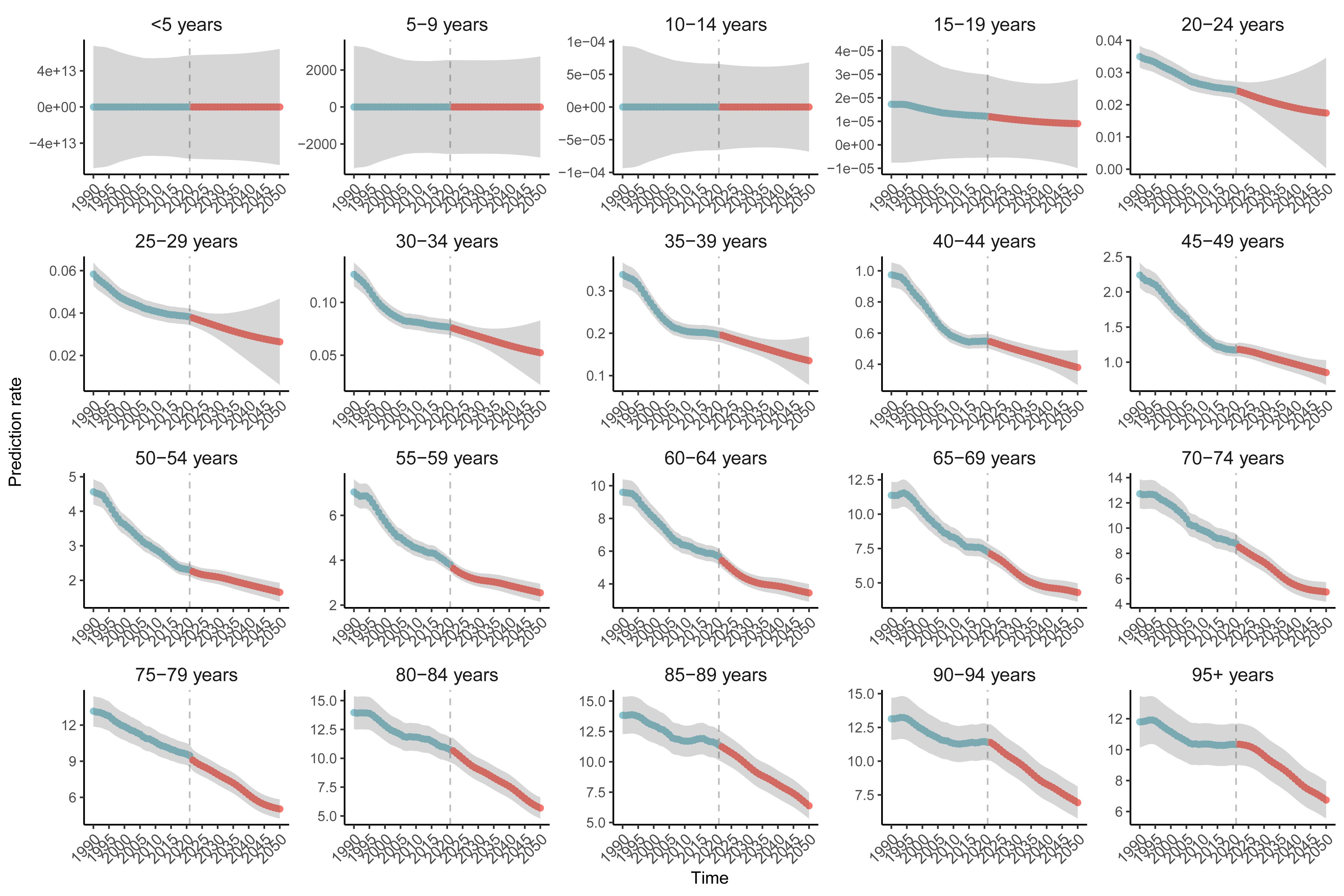 |
| --- |
| **Figure S7. Predicted LC deaths rates across different age groups** |

| 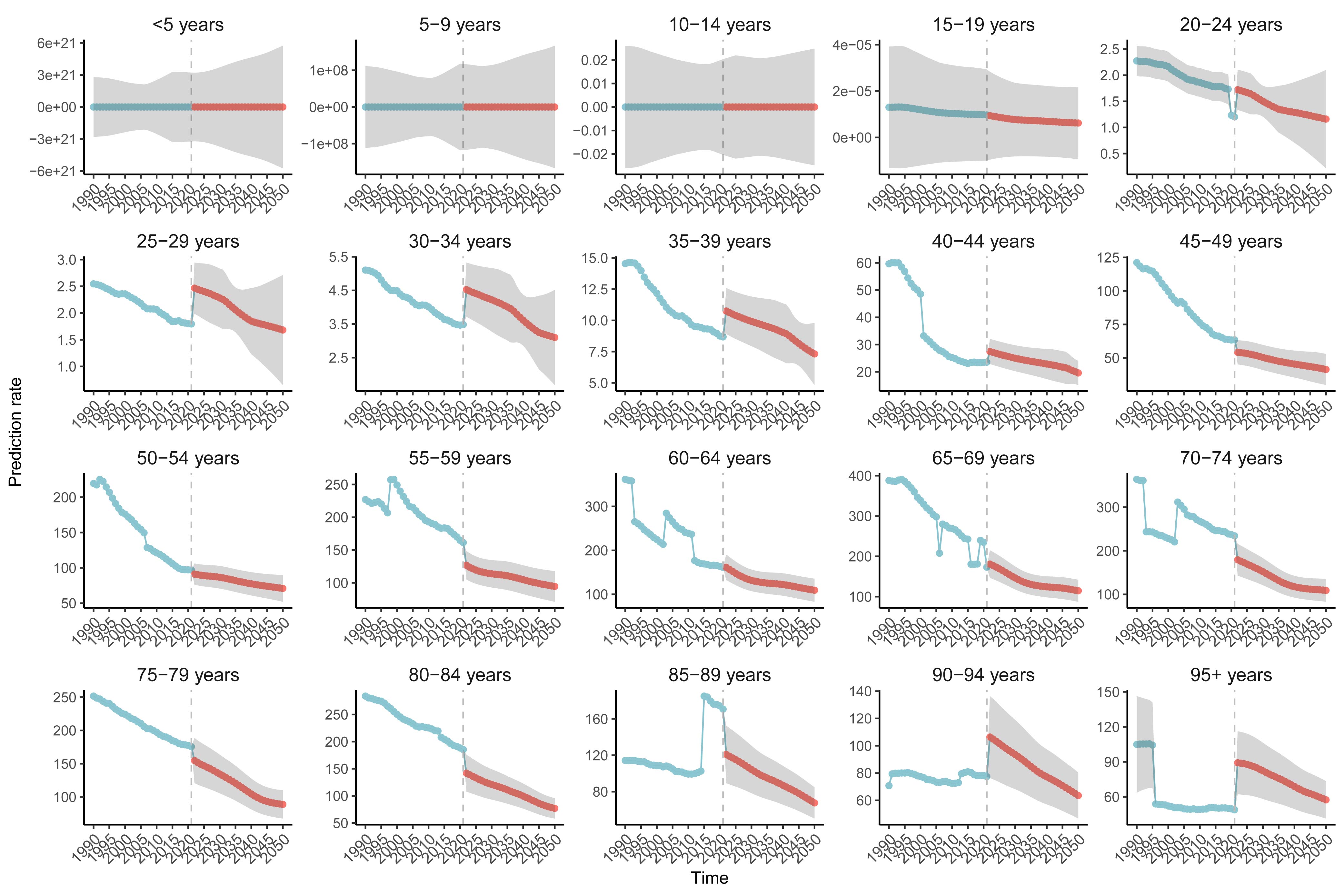 |
| --- |
| **Figure S8. Predicted LC DALYs rates across different age groups** |

| 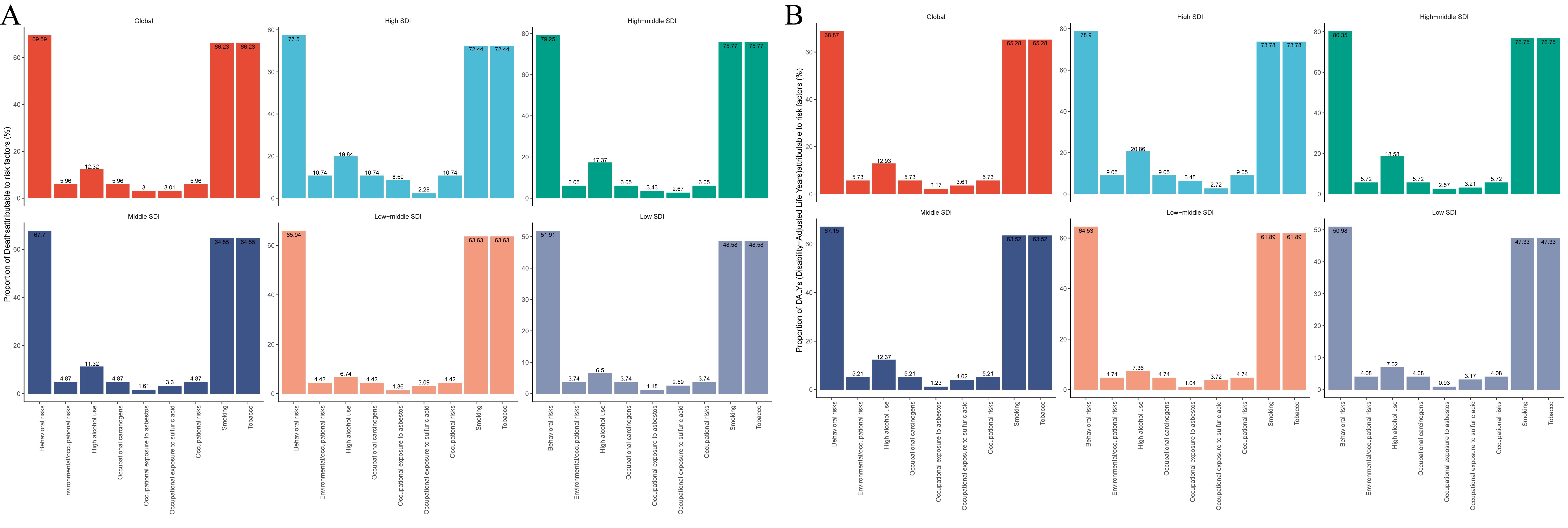 |
| --- |
| **Figure S9.The proportional distribution of the causes of LC within the global scope and five SDI regions.**  **.** |
